# Abstract

**DOI:** 10.1002/pul2.70062

**Published:** 2025-03-20

## 1. Development of Pulmonary Hypertension in Patients Under Mechanical Ventilation Assistance

Rojas A^1^, Vergottini J, Gobbi C, Vanoni S

^1^Universidad Nacional De Cordoba, Ciudad de Córdoba, Argentina

DEVELOPMENT OF PULMONARY HYPERTENSION IN PATIENTS UNDER MECHANICAL VENTILATION ASSISTANCE

**Introduction:** In patients under mechanical ventilatory support (MVA) the development of pulmonary hypertension (PHT) with mean pulmonary artery pressure (mPAP) > 20 mmHg and increased pulmonary vascular resistance (PVR) is a possibility.

**Objectives:** General: To determine mean pulmonary artery pressure values and other hemodynamic variables using the Swan Ganz catheter. Specific: a) to correlate D‐dimer levels and mean pulmonary artery pressure; b) to determine the relationship between mean pulmonary artery pressure, pulmonary vascular resistance and mortality.

**Material and Methods:** Observational, descriptive, prospective, longitudinal study. It included 14 patients of both sexes between 18 and 80 years old, who were admitted to the ICU of the Hospital Nacional de Clínicas with need of invasive mechanical ventilation and right catheterization between 2021 and 2023.

**Results:** Fourteen patients were included. Fifty‐seven percent were male. The mean age was 70.36 ± 2.35 years. The main etiologies were: community pneumonia (21%), septic shock (14.29%) and subarachnoid hemorrhage (14.29%). AVM parameters: PC 13.6 ± 0.9 cmH2O, FiO2 0.49 ± 0.05 mmHg, PEEP 6.3 ± 0.46 cmH2O. Hemodynamic measurements were PAPm 23.68 ± 0.89 mmHg, Wedge 14.19 ± 0.93 mmHg, PVR 1.49 ± 0.18 uW and transpulmonary gradient (TPG) 9.55 ± 0.72 mmHg. Some 92.86% had PAPm ≥ 20 mmHg. Wedge pressure (p < 0.00001) and GTP (p < 0.0001) were predictors of PHT. Significant differences were observed between D‐dimer and PAPm levels (p < 0.001). Among the deceased, PAPm and PVR increased.

**Conclusions:** PTH was observed with elevated PAPm, Wedge and GTP with increased levels of DD. Elevated PAPm and PVR were associated with higher mortality and longer stay in the ICU.

**Keywords:** Pulmonary hypertension; mechanical ventilation; intensive care unit.

## 2. Effects of Sea Level Descent on Pulmonary Hemodynamics and 6‐Minute Walk in Patients With Pulmonary Vascular Disease Residing at High Altitude in Quito (2840 M)

Ulrich S^1^, Hoyos R^2^, Furian M^3^, Schneider S^3^, Herzig J^3^, Saxer S^3^, Bauer M^3^, Mayer L^1^, Schwarz E^1^, Cajamarca E^2^, Mueller J^3^, Lichtblau M^1^

^1^University Hospital Zurich, Zurich, Switzerland, ^2^Carlos Andrade Marín Hospital, Ecuador, Ecuador, ^3^University of Zurich, Zurich, Switzerland

**Background:** Worldwide, > 220 Mio people live > 2000m, many of whom have preexisting chronic conditions, including pulmonary vascular diseases (PVD) defined as pulmonary arterial and chronic thromboembolic pulmonary hypertension (PAH/CTEPH). We investigated the effects of 2‐day sea level (SL) descent as a weekend getaway on pulmonary hemodynamics by echocardiography in PVD‐patients permanently living > 2500 m.

**Methods:** Patients with PVD, diagnosed as PAH or CTEPH by right heart catheterization, who reside in Quito at 2850 m, were transported by bus to SL and stayed there for two days. 6‐minute walk distance (6MWD) test, echocardiography and blood gases were performed at 2850 m and after the first (SL‐day1) and second night (SL‐day2) at SL and the systolic pulmonary artery pressure (sPAP, as tricuspid regurgitation pressure gradient), cardiac output (CO, by left ventricular outflow tract velocity time integral) and other parameters assessed.

**Results:** 28‐patients (24 female, 26 PAH, age 46 ± 12 yrs, PaO2 7.6 ± 1.5 kPa), treated with phosphodiesterase‐5‐inhibitors (N = 27) and endothelin‐receptor antagonists (N = 9) were included. The sPAP in Quito was 68 ± 24 mmHg and decreased to 57 ± 22 and 55 ± 21 mmHg SL‐day1 and SL‐day2 (mean change (95%CI)): ‐11 (‐15 to‐6) and ‐13 (‐17 to‐8), p < 0.001) with SL‐PaO2 10.5 ± 2.2kPA. CO was 3.9 ± 1.1 l/min and HR 74 ± 13 in Quito, CO and HR decreased by ‐0.7(‐1.0 to ‐0.5)l/min and ‐13(‐16 to‐9) until SL‐day2 and sPAP/CO was unchanged. 6MWD was 462 ± 99 m in Quito, increased +42 (21 to 64) at SL‐day1, but was similar to Quito SL‐day2. All patient felt well until return to Quito.

**Conclusion:** In this first study investigating prevalent patients with PAH or CTEPH, near their residential altitude at 2850 m and SL, we showed that decent to SL significantly lowered blood oxygenation, sPAP, and CO with an unchanged total pulmonary resistance, while the 6MWD was higher at SL‐day1 but equal to 2850 m at SL‐day 2. No re‐entry problems occurred upon return to 2850 m.

## 3. The Comprehensive Genetic Analysis of Pulmonary Arterial Hypertension in Multi‐Center Japanese Registry

Isobe S^1^, Tamura Y^1^

^1^International University of Health and Welfare, Tokyo, Japan

**Background:** BMPR2 is the most common risk gene associated with PAH. The genes related to BMP signaling including ACVRL1, ENG, GDF2, and SMAD9 were reported as pathogenic genes for PAH. The genes not directly influencing BMP signaling such as CAV1, KCNK3 and TBX4 have also been reported as pathogenic in PAH. However, there is a no comprehensive genetic analysis for these mutations in multi‐center registry in Japan.

**Methods:** Group 1 PAH patients were recruited from 12 hospitals in Japan. We performed genomic analysis targeting 14 genes (BMPR2, ACVRL1, ENG, SMAD9, CAV1, KCNK3, EIF2AK4, TBX4, ATP13A3, GDF2, SOX17, AQP1, ABCC8 and RNF213), known to be relevant to PAH, using a next‐generation sequencing. We focused on low‐frequency base substitutions and the presence of short insertions or deletions, analyzing variants not documented in ExAC, gnomAD, or ToMMo (for Japanese) databases. Variants with frequencies < 0.01% were extracted and those were compared to mutations reported in ClinVar and HGMD.

**Results:** Genetic testing was conducted on 135 PAH patients in this registry. Known mutations in BMPR2 were identified in16 patients and in ACVRL1 in 2 patients. The novel‐variant candidates were identified in SOX17 (n = 1), BMPR2 (n = 9), SMAD9 (n = 3), and ACVRL1 (n = 4), TBX4 (n = 1), GDF2 (n = 1) and ABCC8 (n = 1). The variant p.Arg4810Lys in RNF213, which was reported previously from Japan, was observed in 6 patients. No variants were detected in ATP13A3 and EIF2AK4 in this study. Seven patients have double variants in this study.

**Conclusion:** Our study identified the mutations previously reported in this Japanese registry. This study contributes to the understanding of the genetic landscape of PAH in Japan, offering potential insights for future diagnostic and therapeutic strategies.

## 4. Measurement of Functional Pulmonary Capillary Surface Area After Balloon Pulmonary Angioplasty for Chronic Thromboembolic Pulmonary Hypertension

Langleben D^1^, Orfanos S^2^, Abualsaud A^1^, Hirsch A^1^, Giovinazzo M^1^, Fox B^3^, Lesenko L^1^, Kaddis M^1^, Ramrup N^1^, Catravas J^4^

^1^Jewish General Hosp/McGill Univ., Montreal, Canada, ^2^Evangelismos Hosp./Univ. of Athens, Athens, Greece, ^3^Yitzchak Shamir Hospital/Tel Aviv University, Tzrifin, Israel, ^4^Old Dominion University, Norfolk, USA

**Background:** Chronic thromboembolic pulmonary hypertension (CTEPH) is caused by partial or complete occlusion of pulmonary arteries by organized thrombus, affecting perfusion of downstream pulmonary capillaries in regions fed by the occluded arteries. We have already demonstrated that functional capillary surface area (FCSA) is decreased in resting humans with CTEPH. Balloon pulmonary angioplasty (BPA) to re‐establish flow through the affected arteries has become an effective treatment option in appropriate patients. The amount of capillary perfusion that is regained during a session of BPA is unknown. We hypothesized that we could measure an increase in FCSA in patients undergoing BPA.

**Methods/Results:** Subjects are undergoing their first session of elective BPA. FCSA assessment involves measuring the first‐pass transpulmonary hydrolysis of the substrate ³H‐benzoyl‐Phe‐Ala‐Pro (BPAP) as it interacts with the endothelial ectoenzyme, angiotensin converting enzyme. At cardiac catheterization, trace quantities of BPAP are injected into the right atrium and transit the lung circulation, and then effluent systemic arterial blood is collected and processed. Radioactivity levels (parent and metabolite) are measured. The instantaneous % metabolism and hydrolysis are calculated, as well as FCSA. After completion of the BPA session, the extent of which is based on clinical needs, FCSA is re‐measured with another injection of BPAP. We have already performed measurements in 10 subjects, with measurable improvements in FCSA. In our study design, to detect a 33% average increase in FCSA in the cohort, with a power of 0.9, we estimate needing at least 27 subjects (NCT05719415).

**Conclusion:** We demonstrate the feasibility of detection of improvement of FCSA in CTEPH patients undergoing BPA. We will report the relationship between the degree of improvement in FCSA, and the number of arterial segments dilated, and the improvement in pulmonary hemodynamics.

## 5. Medication Adherence and Clinical Outcome in Patients With Pulmonary Vascular Diseases

Lichtblau M^1^, Reimann L, Preiss H, Müller J, Huber P, Mayer L, Langleben D, Ulrich S

^1^University Hospital Zürich, Zürich, Switzerland

**Introduction:** In pulmonary arterial hypertension (PAH) and distal chronic thromboembolic pulmonary hypertension (CTEPH), the consistent use of disease‐specific therapies is crucial. We aimed to investigate medication adherence to oral disease‐specific medication and the impact on clinical outcome among patients with PAH or CTEPH to identify potential patient‐related reasons for treatment incompliance.

**Study Design and Methods:** This prospective study focused on medication adherence using a multimeasure approach, including specialty pharmacy order data to calculate medication possession ratio (MPR) and self‐reporting via questionnaire among patients with PAH or CTEPH. Adherence rates of ≥ 80% were considered adherent. Simplified four‐strata risk categories according to the 2022 ERS/ESC PH‐guidelines were determined.

**Results:** We included 93 patients (66% females, 75% PAH, 25% CTEPH, 57 ± 17 years), all on PH‐targeted oral medication between 2013 and 2023. Overall, a number of 73 patients (78%) were classified adherent. The mean MPR was 98 ± 19% and the mean value of questionnaire responses was 89 ± 10%. At the end of the observation period, adherent patients improved their risk‐category, while non‐adherent patients did not. Predictors for adherence were older age and being classified in a higher risk category. Patients with adverse drug reactions were 75% more likely to be non‐adherent to medication (OR = 0.25; 95% CI = 0.08‐0.77).

**Conclusion:** In this collective, mean MPR and self‐reported adherence was overall high with 78% of patients classified as adherent. Adherent patients improved clinical outcomes contrary to non‐adherent patients. Insufficient adherence and potential contributing factors should be regularly considered, especially in patients without improvement after starting disease‐specific therapy.

## 6. A Single‐Cell Lung Atlas of Human Pulmonary Arterial Hypertension

Dai Z^1^, Zhao H, Yi D, Liu B, Hong J, Fallon M

^1^Washington University In St. Louis, Saint Louis, USA

Pulmonary arterial hypertension (PAH) is a disaster disease characterized by obliterative vascular remodeling and persistent increase of vascular resistance, leading to right heart failure and premature death. Understanding the cellular and molecular mechanisms will help develop novel therapeutic approaches for PAH patients.

We hypothesize that single‐cell RNA sequencing analysis can help understand the cellular and molecular mechanisms that drive the disease initiation and progression.10 healthy donors, 10 idiopathic PAH, 10 drug and toxin induced PAH, 10 systemic and pulmonary shunting induced PAH patients from both male and female, and different ethical groups and races, and ages were obtained from PHBI. Lung tissue specimens were processed for fixed scRNA‐seq. Our analysis reveals 317,414 cells, 33 clusters and 9 major cell types, including endothelial cells (ECs), fibroblast, smooth muscle cells (SMCs), pericytes, myeloid cells, epithelial cells. Cell proportion analysis demonstrated an increase in the proportions of venous ECs, fibroblasts, alveolar macrophages, B cells and a reduction in capillary ECs and pericytes in PAH lungs. KEGG Pathway analysis showed that upregulated pathways related Herpes simplex virus 1 infection, ECM‐receptor interaction, Complement and coagulation cascades, TGF‐beta signaling pathway, and downregulated pathways related to IL‐17 signaling, TNF signaling, MAPK signaling pathway were enriched in the PAH patients. Female PAH patients exhibit an increase of myofibroblasts and platelets proportion compared to male PAH patients. Pathway analysis showed that p53 pathway was upregulated, whereas Inflammatory Response, Hypoxia, Epithelial Mesenchymal Transition were downregulated in female patients. PAH patients older than 21 years exhibit an increase in the cell proportion of Platelet, SMCs, Arterial ECs and Venous ECs, and alveolar macrophages and a reduction of B cells and plasma cells.

Our integrated analysis of human PAH lung atlas dataset provides a comprehensive understanding of lung cell populations and molecular signature in PAH patients with different sex, age and subclasses.

## 7. Unilateral Chronic Thromboembolic Pulmonary Disease: Do They Benefit From Surgery? Case Series From Three Cteph Centers

Rashidi F^1^, Yıldızeli B^2^, Parvizi R^3^, Tas S^4^, Yıldızeli Ş^5^, Mutlu B^6^, Bilejani E^3^, Mahmoodian B^7^, Bakhshandeh H^8,9^, Mousavi‐Aghdas S^1^, Heresi G^10^

^1^Tuberculosis and Lung Disease Research Center, Tabriz University of Medical Sciences, Tabriz, Iran, Iran, ^2^Department of Thoracic Surgery, Marmara University School of Medicine, Istanbul, Istanbul, Turkey, ^3^Cardiovascular Research Center, Tabriz University of Medical Sciences, Tabriz, Iran, Tabriz, Iran, ^4^University of Health Sciences, Kartal Koşuyolu Teaching and Education Hospital, Department of Cardiovascular Surgery, Istanbul, Turkey, Istanbul, Turkey, ^5^Marmara University School of Medicine, Department of Pulmonology and Intensive Care, Istanbul, Turkey, Istanbul, Turkey, ^6^Marmara University School of Medicine, Department of Cardiology, Istanbul, Turkey, Istanbul, Turkey, ^7^Medical Radiation Sciences Research Team, Tabriz University of Medical Sciences, Tabriz, Iran, Tabriz, Iran, ^8^Rajaie Cardiovascular Medical and Research Center, Iran University of Medical Sciences, Tehran, Iran, Tehran, Iran, ^9^Student Research Committee, Iran University of Medical Sciences, Tehran, Iran, Tehran, Iran, ^10^Department of Pulmonary and Critical Care Medicine, Respiratory Institute, Cleveland Clinic, Cleveland, OH, USA, Cleveland, USA

**Background:** Unilateral chronic thromboembolism pulmonary hypertension (CTEPH) is exceedingly uncommon. There is limited information on the safety and efficacy of pulmonary endarterectomy (PEA) in this population. We investigated the effectiveness of PEA in these unique category.

**Methods:** This multi‐center study included patients with unilateral CTEPH from three referral centers in the United States, Turkey, and Iran. We evaluated the patients’ demographic information, 6‐minute walk test (6MWD), the New York Heart Association (NYHA) functional class, and hemodynamics.

**Result:** Out of 1,031 patients who had underwent PEA, 39 (3.7%) patients had pure unilateral involvement, of whom 28 (71.8%) were female. There was a significant improvement in the mean pulmonary artery pressure (mPAP, 26 mmHg vs. 21 mmHg, p = 0.011), and pulmonary vascular resistance (PVR, 202 vs. 136 dynes * sec1 * cm‐5, p = 0.014). Also, there was a significant improvement in NYHA functional class (p < 0.001) and 6MWD (360 vs. 409 meters, p < 0.001). In patients with normal hemodynamic parameters at rest (CTED, n = 9), there was no significant change in median 6MWD (448.5vs. 449 m, p = 0.208), mPAP (19 mmHg vs. 16.5 mmHg, p = 0.397), and PVR (129 vs. 84.5 dynes * sec1 * cm‐5, p = 0.128). The most common post‐operative complication was ipsilateral pleural effusion. Only one patient needed extracorporeal membrane oxygenation support. No patient died within the one‐year follow‐up.

**Conclusion:** PEA was a safe and effective method to improve the symptoms and hemodynamic parameters of patients with unilateral CTEPH. Considering PEA in symptomatic patients with unilateral chronic thromboembolic disease will be suitable.

## 8. The Role of Smyd2/Runx2 Axis in Right Ventricular Hypertrophy and Failure

Sapehia V^1^, Weismann N^1^, Seeger W^1,2^, Novoyatleva T^1^, Schermuly R^1^

^1^Cardio‐Pulmonary Institute (CPI), Department of Pulmonary Pharmacotherapy Universities of Giessen and Marburg‐Lung‐Center (UGMLC), Justus‐Liebig‐University Giessen, Aulweg 130, 35392, Giessen, Germany, Giessen, Deutschland, ^2^Cardio‐Pulmonary Institute (CPI), Department of Pulmonary Pharmacotherapy Universities of Giessen and Marburg‐Lung‐Center (UGMLC), Justus‐Liebig‐University Giessen, Aulweg 130, 35392, Giessen, Germany, Giessen, Deutschland, ^3^Cardio‐Pulmonary Institute (CPI), Department of Pulmonary Pharmacotherapy Universities of Giessen and Marburg‐Lung‐Center (UGMLC), Justus‐Liebig‐University Giessen, Aulweg 130, 35392, Giessen, Germany; Max Planck Institute (MPI) for Heart and Lung Research, Ludwigstrasse 43, 61231, Bad Nauheim, Germany, Giessen, Deutschland, ^4^Cardio‐Pulmonary Institute (CPI), Department of Pulmonary Pharmacotherapy Universities of Giessen and Marburg‐Lung‐Center (UGMLC), Justus‐Liebig‐University Giessen, Aulweg 130, 35392, Giessen, Germany, Giessen, Deutschland, ^5^Cardio‐Pulmonary Institute (CPI), Department of Pulmonary Pharmacotherapy Universities of Giessen and Marburg‐Lung‐Center (UGMLC), Justus‐Liebig‐University Giessen, Aulweg 130, 35392, Giessen, Germany, Giessen, Deutschland

Pulmonary hypertension (PH) is a life‐threatening disease, characterized by excessive pulmonary vascular remodeling, leading to elevated pulmonary arterial pressure and right ventricular hypertrophy (RVH).The RV is the major determinant of functional state and prognosis in PH.The mechanisms underpinning the development of RV failure are still unexplored. Recent studies demonstrated that an osteogenic transcription factor Runt‐related transcription factor‐2 (RUNX2) plays a pathogenic role in cardiac hypertrophy and failure, and contributes to the development of PH. In this study, we hypothesize the existence of the interplay of the methyl transferase SMYD2 (SET and MYND domain‐containing protein 2) and the RUNX2 in RVH. We found that Smyd2 negatively affects RUNX2 protein expression and it appeared as a key player in RUNX2 methylation, confirmed by in vitro methyl transferase assay. Here,we demonstrated that Smyd2 and RUNX2 interact physically and functionally.Co‐Immunoprecipitation studies revealed that endogenous and overexpressed Smyd2 protein physically interact with RUNX2 protein.Additionally, the impact of gene silencing and overexpression studies of RUNX2 and Smyd2 over one another was analyzed using primary cardiac fibroblasts (CFs) by employing adenovirus‐mediated and si‐RNA‐mediated overexpression and gene silencing approaches respectively.In loss‐of‐function studies RUNX2 silencing reduced CF proliferation and promoted the down regulation of mRNA expression of osteogenic genes as Col1α1, Col3α1, alkaline phosphatase (ALP) and cartilage oligomeric matrix protein (COMP).Notably, Smyd2 knockdown provided opposite effects in CFs.In our experiments, we observed the negative effects of Smyd2 ectopic expression on RUNX2 protein accumulation in CFs.Furthermore, Smyd2 significantly impeded the expression of osteogenic markers such as Col1α1, Col3α1, ALP and COMP both in human and rat CFs.Thus, the main conclusion of our study is that Smyd2 plays a negative role in osteogenesis by suppressing the osteogenic gene program and inhibiting the expression of RUNX2. Furthermore,we will investigate whether RUNX2 serves as a specific therapeutic target for RVH and heart failure.

## 9. Implication of The Protein ATP Citrate Lyase (ACLY) in Vascular Remodeling

Grobs Y^1^, Romanet C^1^, Lemay S^1^, Bourgeois A^1^, Theberge C^1^, El‐Kabbout R, Pelletier A, Voisine P^2^, Sauvaget M^1^, Martineau S, Breuils‐Bonnet S^1^, Potus F^1^, Provencher S^1^, Boucherat O^1^, Bonnet S

^1^IUCPQ ‐ Ulaval, Québec, Canada, ^2^Ottawa heart institute, Ottawa, Canada

Pulmonary arterial hypertension (PAH) is a progressive occlusive vascular disease (OVD) in which pulmonary artery (PA) smooth muscle cells (PASMC) are characterized by excessive proliferation and resistance to apoptosis, like in cancer cells. These features are shared with neointimal hyperplasia (NH) in coronary artery disease (CAD). ATP citrate lyase (ACLY), has recently emerged in cancer as a key player to sustain cellular proliferation and survival by favoring Warburg effect, chromatin acetylation, and lipid synthesis. However, its role in OVD remains unknown. Thus, we hypothesized that ACLY is upregulated in OVD and supports the proliferative phenotype of OVD‐SMCs. Increased expression and nuclear localization of ACLY were observed in 1) OVD‐SMCs from both PAH and CAD patients compared to controls and 2) in PAH and CAD rodent models (SugenHypoxia, SuHx and carotid injury, CI). Inhibition of ACLY decreased both proliferation and survival of OVD‐SMCs and reduced expression of markers associated with glycolysis and lipids synthesis. ACLY's inhibition also enhanced mitochondrial respiration and lowered cholesterol. Further analysis showed that ACLY promotes nuclear acetyl‐CoA production, leading to histone acetylation and GCN5‐mediated transcriptional activation of genes involved in cell cycle progression. In vivo, pharmacological inhibition of ACLY significantly improved pulmonary hemodynamics in SuHx rats and NH in CI rats. Smooth muscle specific Acly K.O mice were resistant to both PAH and NH. Ex vivo, inhibition of ACLY attenuated vascular remodeling in human PCLS and CoA rings exposed to a growth factors. In conclusion, we showed that inhibition of ACLY may represent a novel and attractive therapeutic avenue for OVD.

## 10. Embracing Heterogeneity In Healthcare: A Pathway to Equitable Health Services in Ghana

Salifu M^1^

^1^Rescue Missions International, Accra, Ghana

Healthcare systems in Ghana, like many low‐ and middle‐income countries, face significant challenges in delivering equitable and efficient services to a diverse population. This paper explores the concept of embracing heterogeneity within the Ghanaian healthcare landscape as a key strategy for addressing disparities in health outcomes and service delivery. Ghana's population is marked by diverse socio‐economic, cultural, and geographical characteristics that contribute to uneven access to healthcare. Additionally, the mix of public, private, and informal healthcare providers further complicates the delivery of standardized care. By embracing heterogeneity—recognizing and strategically addressing the varied needs of different communities—Ghana can enhance the adaptability and inclusiveness of its healthcare system.

This study examines the benefits of heterogeneous healthcare approaches, such as decentralization of services, integration of traditional medicine, and the adoption of digital health innovations tailored to local contexts. Moreover, it highlights the role of targeted policies in promoting healthcare equity, especially for marginalized groups such as rural populations, women, and people with disabilities. The paper also considers the importance of collaborative efforts between governmental bodies, local communities, and international organizations to create sustainable and context‐sensitive healthcare solutions. By shifting from a one‐size‐fits‐all model to a more inclusive and responsive approach, Ghana has the potential to create a healthcare system that better meets the needs of its diverse population, ultimately improving health outcomes and achieving universal health coverage. The paper concludes by advocating for policy reforms that prioritize heterogeneity as a critical component of healthcare planning and delivery.

## 11. Delineating Pulmonary Artery Smooth Muscle Cell State Dynamics in Health and Disease

Crnkovic S^1,2^, Thekkekara Puthenparampil H^2^, Mulch S^3^, Biasin V^2^, Wilhelm J^1^, Bartkuhn M^1^, Bonyadi Rad E^2^, Wawrzen A^2^, Matzer I^2^, Mitra A^3^, Leib R^3^, Nagy B^2^, Sahu‐Osen A^2^, Valzano F^2^, Bordag N^2^, Evermann M^4^, Hoetzenecker K^4^, Olschewski A^2^, Ljubojevic‐Holzer S^2^, Wygrecka M^1^, Stenmark K^5^, Marsh L^2^, de Jesus Perez V^3^, Kwapiszewska G^1,2^

^1^Justus Liebig University Giessen, Giessen, Germany, ^2^Medical University of Graz, Graz, Austria, ^3^Stanford University School of Medicine, Stanford, United States of America, ^4^Medical University of Vienna, Vienna, Austria, ^5^University of Colorado, Aurora, United States of America

Phenotypically altered vascular smooth muscle cells are a defining pathogenic feature in pulmonary vascular remodeling. This phenotypic change is marked by the downregulation of canonical, cell type defining markers, such as contractile machinery components, alongside an upregulation of extracellular matrix proteins. We have recently revealed a new layer of biological complexity using single cell resolution approach, which indicated a functional sub‐specialization within the pulmonary artery smooth muscle cell (PASMC) population.

This study aimed to link PASMC heterogeneity with functional impairment and phenotypic alterations observed in pulmonary vascular remodeling. We employed orthogonal sequencing and deep cellular phenotyping approach to characterize freshly isolated and early passaged PASMCs from both healthy individuals and patients with idiopathic pulmonary arterial hypertension (IPAH). These analyses were further supported by proteomics profiling.

Our findings indicate that while IPAH PASMCs retain key cell‐type‐defining characteristics, they adopt a distinct functional state. A hallmark of IPAH PASMCs, both in situ and in early passage cultures, includes lower proliferation rate, reduced expression of contractile proteins, and metabolic reprogramming—such as the hexosamine biosynthetic pathway, which supports increased demand for glycosaminoglycan and proteoglycan production. Lower mitochondrial content displaying hyperpolarized state and heightened reactive oxygen species (ROS) production was an additional prominent disturbance in diseased PASMC. Analysis of intercellular communication revealed that IPAH PASMCs exhibit increased susceptibility to external cues, especially extracellular matrix components and soluble factors. Using a co‐culture system with adventitial fibroblasts from matched donors, we identified several factors that can partially induce PASMC phenotypic transitions.

These findings provide fundamental insights into the cellular dynamics underlying pulmonary vascular disease and offer a deeper understanding of the pathogenesis of pulmonary arterial hypertension.

## 13. Smaller Right Ventricular Volumes Correlate With Elevated Afterload in Unaffected Carriers of a Pathogenic Bmpr2 Variant

Toth E^1,2^, Celant L^1,2^, Meijboom L^2,3^, Vonk Noordegraaf A^1,2^, de Man F^2,4^, Bogaard H^1,2^

^1^Department of Pulmonary Medicine Amsterdam UMC, Amsterdam, the Netherlands, ^2^Pulmonary Hypertension and Thrombosis, Amsterdam Cardiovascular Sciences, Amsterdam, the Netherlands, ^3^Department of Radiology and Nuclear Medicine Amsterdam UMC, Amsterdam, the Netherlands, ^4^PHEniX Laboratory, Department of Pulmonary Medicine Amsterdam UMC, Amsterdam, the Netherlands

**Introduction:** Mutations in the BMPR2 gene are a significant genetic risk factor for hereditary pulmonary arterial hypertension (PAH). Studies have shown that patients with BMPR2‐associated PAH experience more severe right ventricular (RV) impairment compared to those with idiopathic PAH, despite having similar afterload. This suggests that BMPR2 might directly influence cardiac structure and function. Recent findings from novel transgenic BMPR2 rat models have revealed intrinsic RV dysfunction that is independent of pulmonary hypertension (PH), prompting the question of whether similar differences might be observed in healthy human BMPR2 carriers.

**Method:** 29 carriers of a pathogenic BMPR2 variant (mean age 43 ± 15 years, 58% female) and 20 healthy controls (mean age 43 ± 18 years, 45% female) underwent cardiac magnetic resonance imaging (cMRI) as part of a multimodal, ongoing, prospective screening program. 22 carriers of a pathogenic BMPR2 variant additionally underwent right heart catheterization. Images were analyzed to assess cardiac volumetrics and mass to compare with healthy controls. Using the single beat method, pressure‐volume (PV) loops were constructed to assess load independent intrinsic RV function, with cardiac volumes derived from CMRI and pressures measured during RHC at the same day.

**Results:** Carriers of a pathogenic BMPR2 variant exhibited smaller indexed right ventricular end‐diastolic (RVEDVi)(79.54 □ 17.55 vs. 62.59 □ 15.34; p = 0.001) and end‐systolic (RVESVi) (34.16 □ 10.51 vs. 27.06 □ 8.34; p = 0.014) volumes than control subjects. Lower RVEDVi (R = ‐0.69; p = 0.0122 and RVESVi (R = ‐0.63; p = 0.028) correlated with elevated PV‐loop derived afterload (Ea) in carriers of a pathogenic BMPR2 variant.

**Conclusion:** Carriers of a pathogenic BMPR2 variant exhibit reduced cardiac volumes, which paradoxically correlate with increased afterload. These differences may stem from the role of BMPR2 in embryonic cardiac development, where deficiencies could lead to alterations in both the pulmonary vascular bed and cardiac structure.

## 14. Ct‐Based Quantification of Pulmonary Vascular Volumes Demonstrates Greater Vascular Heterogeneity and Central Distribution in Chronic Thromboembolic Pulmonary Hypertension Compared With Pulmonary Arterial Hypertension and Control Groups

Synn A^1^, Nardelli P^2^, Vegas Sanchez‐Ferrero G^2^, Ross J^2^, San José Estépar R^2^, Waxman A^2^, Washko G^2^, San José Estépar R^2^, Heresi G^3^, Rahaghi F^2^

^1^Beth Israel Deaconess Medical Center, Boston, United States, ^2^Brigham and Women's Hospital, Boston, USA, ^3^Cleveland Clinic, Cleveland, USA

**Background:** Despite availability of effective therapies, chronic thromboembolic pulmonary hypertension (CTEPH) remains difficult to diagnose, leading to undertreatment and excess morbidity. Image‐based vascular biomarkers may complement existing tools to expedite evaluation of CTEPH. In this study, we sought to determine if measures of vascular heterogeneity and central redistribution on chest CT differed between CTEPH, pulmonary arterial hypertension (PAH), and control groups.

**Methods:** We included 108 patients who underwent invasive right heart catheterization and chest CT between 2011‐2018. Automated image analysis was used to generate three‐dimensional reconstructions of the pulmonary vasculature, from which we calculated the volumes of all arteries, all veins, and small arteries and veins (area< 5mm2). Vascular heterogeneity was assessed by partitioning each lung into isovolumetric segments and calculating coefficients of variation (CV) across segments. Central redistribution was assessed by measuring vascular volumes in the central and peripheral lung zones (innermost/outermost fifth, respectively) and calculating central‐to‐peripheral volume ratios. We constructed multivariable linear regression models (adjusting for age/sex) to compare metrics of vascular heterogeneity and redistribution between CTEPH and control/PAH groups.

**Results:** Of 108 patients, 21 had CTEPH, 47 had PAH, and 40 were controls. We found consistently higher CVs (greater vascular heterogeneity) in the CTEPH group vs. controls. For example, for small arterial volume, CV was 0.09 units higher (95%CI 0.04—0.14, p = 0.0004) in the CTEPH group. Similarly, we found small vessel CVs were higher in CTEPH vs. PAH. We also found higher central‐to‐peripheral volume ratios (i.e. greater central redistribution) in the CTEPH group. For small arterial volume, central‐to‐peripheral volume ratio was 1.52 units higher in CTEPH vs. controls (95%CI 0.78—2.26, p = 0.0001). Similar results were seen between CTEPH and PAH groups.

**Conclusion:** Volumetric measures of pulmonary vascular heterogeneity and central distribution can be quantified using CT techniques and may contribute to an image‐based signature of CTEPH.

## 15. Acute Pulmonary Vasoreactivity in a Patient With Pulmonary Arterial Hypertension and a Heterozygous Variant of Unknown Significance in ABCC8

Gédéon T^1^, Lesenko L^1^, Langleben D^1^

^1^Jewish General Hospital/McGill University, Montreal, Canada

Acute pulmonary vasoreactivity testing is a cornerstone initial evaluation for patients with seemingly idiopathic pulmonary arterial hypertension (IPAH). Acute vasoresponders are directed towards high dose calcium channel blocker therapy rather than conventional PAH therapies. The available evidence suggests that their vascular abnormality is one of vascular tone rather than cell‐proliferation as in other types of PAH. True vasoresponders carry an excellent prognosis, as long as they remain on therapy. There are no specific genetic markers to identify responders. We describe a physically active 27‐year‐old female who presented with 4 episodes of exertional syncope and dyspnea. Standard evaluation for pulmonary hypertension supported the diagnosis of IPAH. At catheterization, her mean pulmonary artery pressure (mPAP) was 43 mm Hg, pulmonary artery wedge pressure (PAWP) 8 mm Hg and cardiac output (CO) 4.40 L/min. Acute vasoreactivity testing using inhaled nitric oxide led to a decrease in mPAP to 25 mm Hg, PAWP 10 mmHg and CO 4.97 L/min. She was initiated on high dose calcium channel blockade and is responding well. Genetic testing revealed a heterozygous variant of unknown significance (VUS) in the gene ATP‐binding cassette subfamily member 8 (ABCC8 c.2158c>t P.Ser720Phe). This variant is predicted to be potentially pathologic via loss‐of‐function. ABCC8 encodes sulfonylurea receptor‐1 (SUR‐1), an ATP‐sensitive potassium channel regulatory subunit Kir6.2 expressed in the pulmonary vasculature. Heterozygous ABCC8 variants have been associated with phenotypic IPAH. Given that ABCC8 variants affect a vascular ion channel, the finding of vasoresponsiveness might be expected. Nonetheless, none of the other vasoresponsive patients in our clinic have ABCC8 variants. To our knowledge, this is the first case of vasoreactive PAH described with a heterozygous mutation in ABCC8. Although coincidental occurrence cannot be excluded, further research should explore the potential mechanisms of acute vasodilator responsiveness in loss‐of‐function potassium channel mutations (SUR1).

## 16. A Follow‐Up Study: Delta‐Like Ligand 4 Inhibitors Induce Pulmonary Hypertension – 13 Randomized Clinical Trials

Puerta C^1^, Besse C^1^, Winicki N^1^, Thistlethwaite P^1^

^1^University Of California, San Diego. Division of Cardiothoracic Surgery, San Diego, United States

**Background:** Inhibition of Delta‐Like Ligand 4 (DLL‐4) within the pulmonary vasculature allows for unopposed JAG‐1‐NOTCH3 signaling, leading to pulmonary vascular smooth muscle cell (vSMC) proliferation and development of pulmonary arterial hypertension (PAH). New monoclonal antibodies against tumor angiogenesis targeting DLL‐4 are being tested in clinical trials.

**Methods:** PubMed and ClinicalTrials.gov were queried for studies on DLL‐4 inhibition. Adverse events were categorized according to National Cancer Institute Common Terminology Criteria for Adverse Events. Pulmonary hypertension (PH) grades were defined as ranging from mild dyspnea (Grade 1) to life‐threatening pulmonary compromise (Grade 4) or death (Grade 5). PH degree was determined by the clinical trial investigators using echocardiography and cardiac catheterization.

**Results:** Thirteen clinical trials (phase 1, 1a, 1b, and 2) between 2011‐2023 investigating DLL‐4 or DLL‐4/VEGF inhibitors were included. Demcizumab was the most common monoclonal antibody used for treatment, followed by Navicixizumab, Delpacimab, ABL001, and Enoticumab. In total, 672 patients (median age 60, 52.5% female) underwent treatment. Seventy‐four new PH cases were reported (mean incidence of 11.7%[ ± 5.8%]). Four PH patients manifested left heart failure. PH grades 1‐2 represented 81% (n = 60) of cases, and 19% (n = 14) were grades 3‐5. PH incidence was higher in bispecific DLL‐4‐VEGF inhibitor trials versus sole DLL‐4 inhibitor trials (16.2% vs 7.9%). Phase 1 and 2 trials displayed similar PH incidences (11.4% vs 13.0%). After drug cessation, five studies reported PH resolution while two reported clinical improvement of PH.

**Conclusions:** PH was a side effect of DLL‐4 inhibition treatment for malignancies. These results follow our previous research demonstrating that JAG‐1‐NOTCH3 signaling induces pulmonary vSMC proliferation and PAH. The fact that individuals showed resolution of PH after drug cessation supports that DLL‐4 inhibitors are the causal agent. Additional studies are needed to optimize oncological benefits while decreasing the risk of PH development in patients receiving these drugs.

## 17. Validation of Emphasis‐10 as a Digital Patient‐Reported Outcome Measure in Pulmonary Arterial Hypertension

Varian F^1^, Newman J^3^, Hitchcock F^2^, Burney R^1^, Rawlings G^4^, Harrington J^2^, Goh Z^1^, Ablott J^2^, Zafar H^1^, Armstrong I^2^, Toshner M^3^, Rothman A^1^

^1^University Of Sheffield, Sheffield, United Kingdon, ^2^Sheffield Teaching Hospitals NHS Foundation Trust, Sheffield, United Kingdom, ^3^Royal Papworth Hospital, Cambridge, United Kingdom, ^4^Clinical and Applied Psychology Unit, University of Sheffield, Sheffield, United Kingdom

**Introduction:** Digital capture of health‐related quality of life (HRQoL) using patient‐reported outcome measures (PROMs) is recommended by international stakeholders and regulatory bodies to improve clinical efficiency, data capture and equitable care. EmPHasis‐10 is a pulmonary hypertension PROM validated in three continents for use in clinical practice and trials, however it is currently validated in paper format only. This limits potential use in decentralised clinical trial designs and clinical decision‐making.

**Aim:** To validate paper EmPHasis‐10 in digital format

**Methods:** Patients were consented into Cohort‐Digital (IRAS 123349, REC 13/EE/0203), a UK multi‐centre prospective observational study. PROM completion was anchored to patient‐reported stability using the single‐item subject global assessment. EmPHasis‐10 was delivered on paper and digitally via the Atom5TM app in its original format. Sample size is in line with international psychometric property guidelines. Bland‐Altman and Spearman's were performed for validation.

**Results:** Of the N = 51 patients, median age was 53 years (IQR 41‐62 y), 71% were female, 81% white and median time since diagnosis was 5 years (IQR 1‐11.5 y). 82% of patients were defined as low/intermediate‐low risk by COMPERA 2.0 risk score and WHO FC I/II/III/IV was 7%/41%/50%/2% respectively. Median time between digital and paper completion was +1 day (IQR, 0 to 5). All patients reported stable HRQoL at the time of completion. Mean scores were equal at 21/50 (±14) with SEM 2. Scores were consistent between paper and digital formats (Spearman's r = 0.98, p < 0.0001, Cronbach's alpha 0.99). Bland Altman bias 0.22 (SD 2.7) with 95% Limits of Agreement (‐4.9‐5.4) below the reported minimal clinically important difference threshold (Δ6).

**Conclusion:** Digital EmPHasis‐10 shows strong criterion validity with the paper format. Both formats are valid and can be selected in accordance with patient preference. Future analysis will evaluate longitudinal adherence, cross‐cultural validity and responsiveness to treatment change in clinical trials.

## 18. Utilizing Novel 18F‐Fluoroglutamine PET Imaging in Patients With Pulmonary Arterial Hypertension

Al Aaraj Y^1^, Lopresti B^1^, Kelly N^1^, Sun W^1^, Tavakoli S^1^, Ouerfelli O^2^, Lewis J^2^, Tomassoli I^1^, Chan S^1^

^1^University Of Pittsburgh School Of Medicine, Pittsburgh, United States, ^2^Memorial Sloan Kettering Cancer Center, New York, United States

**Background:** Pulmonary arterial hypertension (PAH) is mortal disease affecting lung blood vessels. PAH diagnosis is often delayed, and a need exists to develop non‐invasive diagnostic imaging tools, particularly those detecting early disease. In PAH, we found early activation of vascular glutaminase (GLS1), an enzyme that converts glutamine to glutamate (glutaminolysis) and drives vascular stiffness and proliferation. Glutaminolysis also promotes right ventricular remodeling in PAH. In rodent models of PAH, glutamine uptake was increased in diseased pulmonary vessels and right ventricle, as quantified by positron emission tomography (PET) imaging of a 18F‐(2S,4 R)‐4‐fluoroglutamine (18F‐FGln) tracer.

**Objective:** We sought to perform the first‐in‐human PET imaging of 18F‐FGln in PAH to compare glutamine uptake to that of controls.

**Methods and Results:** In a single‐center pilot study at the University of Pittsburgh Medical Center (UPMC), we recruited male and female subjects of 18‐70 years of age who carry a confirmed diagnosis of idiopathic PAH by right heart catheterization, Functional Class II‐III and on standard‐of‐care vasodilator therapy. In comparison, non‐diseased healthy control subjects were recruited without known cardiopulmonary disease. ECG‐gated PET/CT images were acquired and reconstructed after IV administration of 18F‐FGln. By interim analysis of cardiac tissue uptake of 9 subjects (4 controls and 5 idiopathic PAH patients), a significant increase of relative 18F‐FGln uptake in PAH patients was observed within the right ventricle (RV) free wall to that of the interventricular septum (standardized uptake value of RV/septum ratio).

**Significance:** Based on these interim findings, we are poised to complete recruitment and define the potential of this molecule as a novel clinical diagnostic tracer for PAH. These results could advance clinical management of PAH patients and guide clinical trial design to track efficacy of drugs for PAH that target vascular metabolism or proliferation.

## 19. Pulmonary Perfusion Patterns and Right Ventricular Function in Pulmonary Arterial Hypertension Associated With Congenital Heart Disease: Insights From Dual‐Energy CT Imaging

Sivasubramanian R^1^, Kidambi B^1^, Kadiyani L^1^, Singh S^1^, Gupta S^1^, Kumar S^1^, Ohja V^1^, Pandey N^1^, Jagia P^1^

^1^AIIMS New Delhi, New Delhi, India

**Introduction:** Pulmonary arterial hypertension associated with congenital heart disease (PAH‐CHD) is a severe complication of long‐standing congenital heart defects. This study aimed to investigate pulmonary perfusion patterns and right ventricular (RV) function in PAH‐CHD patients using Dual‐Energy CT (DECT) imaging and echocardiography, exploring the relationship between perfusion patterns and clinical parameters.

**Materials and Methods:** Twenty‐five patients with simple congenital heart defects (VSD 64%, ASD 32%, PDA 4%) underwent DECT scans, echocardiography, and six‐minute walk tests (6MWT). DECT perfusion patterns were categorized as normal (NL), diffuse heterogeneously decreased (DH), or peripheral wedging (PW). Echocardiographic parameters included Tricuspid Annular Plane Systolic Excursion (TAPSE) and Global Longitudinal Strain (GLS). Correlations between perfusion patterns, echocardiographic findings, and clinical parameters were analyzed.

**Results:** DECT revealed three main perfusion patterns: DH (48%), NL (32%), and PW (20%). Patients with DH patterns demonstrated worse RV function, with lower TAPSE (mean 20.5 mm) and GLS (‐15.6%) compared to those with NL patterns (TAPSE 23.1 mm, GLS ‐17.2%). DH pattern patients also showed reduced exercise capacity with lower 6MWT distances (365 meters) compared to NL pattern patients (440 meters). Hemoglobin levels were highest in DH pattern patients, suggesting a compensatory response to chronic hypoxia, though this did not translate to improved functional outcomes.

**Conclusions:** DECT and echocardiography provide a comprehensive, non‐invasive assessment of disease progression and RV function in PAH‐CHD patients. The study demonstrates significant correlations between perfusion patterns and clinical parameters, suggesting DECT's potential as a "one‐stop shop" for diagnosis, risk stratification, and monitoring of PAH‐CHD. However, limitations, including small sample size and radiation exposure concerns, warrant further research involving a larger cohort of patients.

## 20. Outcome in Patients With Left to Right Shunts and Pulmonary Arterial Hypertension Undergoing Operability Assessment by Acute Vasodilator Testing With Oxygen

Sivasubramanian R^1^, Kadiyani L^1^, Saxena A^1^, Kothari S^1^, Gupta S^1^, Narang R^1^

^1^AIIMS New Delhi, New Delhi, India

**Background:** Pulmonary artery hypertension associated with simple left to right intracardiac shunts adversely affects the outcome of an otherwise curable lesion. We planned to assess the ability of acute vasodilator testing (AVT) with oxygen to predict the outcome in these cases that are considered borderline operable on clinical evaluation.

**Material and Methods:** A retrospective study included all patients (157 patients) who underwent operability assessment between January 1, 2016, and December 2021. Patients with associated left heart disease or residual post‐operative lesions were excluded. The postoperative outcome was correlated with preoperative variables in patients undergoing surgery.

**Results:** Most patients had a post‐tricuspid shunt lesion (132/157). Out of the total 157 patients, 60 patients underwent surgical correction. The outcomes of right heart failure or death were present in six post‐operative patients and three patients on medical follow‐up. On multivariate analysis, baseline PVRI and PVR/SVR ratio reduction demonstrated an association with the outcome, although it did not achieve a significant proportion. Multiple logistic regression analysis showed the age of more than two years, absence of cardiomegaly, bidirectional or predominant right to left shunting across the defect, and absence of LV volume overload (post tricuspid) on echocardiography predicted an inoperable outcome on AVT. We propose a score based on the above variables for predicting an inoperable outcome on AVT with nearly eighty percent specificity and sixty percent sensitivity.

**Conclusions:** The study highlights the importance of clinical and noninvasive investigations in the comprehensive evaluation of patients with left‐to‐right intracardiac shunts and severe pulmonary arterial hypertension. Larger studies should explore the identification of scores discussed above as predictors of inoperable outcomes on vasoreactivity.

## 21. Efficacy of Intravenous Iron Therapy Compared to Usual Treatment in Iron Deficient Adult Cyanotic Congenital Heart Disease Patients for Improvement in Clinical Outcomes at Three Months – A Randomized Controlled Study

Sivasubramanian R^1^, Singh S^1^, Faisal N^1^, Kadiyani L^1^, Handa A^1^, Gupta S^1^

^1^AIIMS New Delhi, New Delhi, India

**Background:** Intravenous iron therapy is established in the treatment of heart failure. However, its utility is not proven in patients with cyanotic congenital heart disease (CCHD). We aimed to compare the effectiveness of intravenous iron with usual care on a 6‐minute walk distance after 3 months of treatment in adults with CCHD.

**Materials and Methods:** In this prospective randomized study, 27 patients with uncorrected or palliated CCHD with iron deficiency were enrolled (the most common diagnosis is Eisenmenger syndrome). Patients were randomized into two groups: 13 patients were given IV iron therapy, and 14 patients in the second group were given the usual care with oral iron therapy.

**Results:** The mean age of enrolled patients is 23.3 ± 5.6 years. The mean hemoglobin of patients is 16.7 ± 2.0 g/dl. The mean 6 MW distance in the IV iron arm and usual treatment arm is 401 ± 35.2 and 408.6 ± 46.5 meters, respectively, at baseline, which improved to 425 ± 28.7 and 421.5 ± 43.2 meters, respectively, at 3 months follow‐up. The difference of change in 6 MW distance between the two arms is non‐significant (p = 0.157).

The change in WHO functional class at follow‐up was similar in both arms (p = 0.082). There was a significant improvement in WHOQOL‐BREF Score Domains 2, 3, and 4 (which constitute psychological health, social relationships, and environment), and there was a trend towards improvement in Domain 1 score (which constitutes physical health) in the IV iron arm compared to usual treatment arm.

**Conclusions:** This study has demonstrated no improvement in clinical outcomes of 6 MW distance, arterial hemoglobin saturation, and WHO functional class. However, IV iron significantly improved patients' quality of life. Intravenous iron is safe and without any significant side effects in adult patients with CCHD.

## 22. Metoprolol Therapy in Patients With Eisenmenger Syndrome (Mines) Study. A Single‐Center, Double‐Blinded, Randomized, Placebo‐Controlled Trial

Sivasubramanian R^1^, Pius A^1^, Yadav S^1^, Gupta S^1^, Kalaivani M^1^, Seth S^1^, Narang R^1^

^1^AIIMS New Delhi, New Delhi, India

**Background:** Betablockers may prevent sudden cardiac death and worsening heart failure in Eisenmenger syndrome (ES). Observational studies have shown betablockers to be safe and effective in ES. We aimed to study the safety and efficacy of metoprolol succinate therapy in ES.

**Methods:** In this double‐blind, placebo‐controlled study, we randomly assigned 60 patients with ES to receive a placebo or 25 mg of metoprolol succinate OD for 2 weeks, followed by 25 mg BD for 14 weeks. The primary outcome was the mean difference in the 6‐minute walk test (6MWT) distance. Secondary outcomes were clinical composite outcome (all‐cause death, heart failure hospitalization, worsening of heart failure, change in WHO functional class, and new onset syncope), change in resting saturation, TAPSE, and NT Pro BNP level at 16 weeks.

**Results:** At 16 weeks, the mean difference in 6MWT distance between the treatment group and placebo group as per protocol analysis was 6 meters (95% CI ‐ 7.43 to 19.49; P 0.37), and as per intention to treat analysis was ‐1.1 meters. Clinical composite outcome occurred more in the treatment group 5(17%) than in the placebo group 1(3%) (P 0.197). This was primarily driven by worsening of heart failure in the treatment group. Compared to the baseline, the metoprolol group had a 37% increase in NT pro‐BNP level, while the placebo group had a 13% reduction in NT pro‐BNP level. There was no difference in TAPSE 0.05 (95% CI ‐0.62 to 0.72; P 0.88) or resting saturation ‐0.06 (95% CI ‐0.88 to 0.75; P 0.87) between the two groups at 16 weeks.

**Conclusion:** Metoprolol succinate therapy did not improve the 6‐minute walk distance at 16 weeks among patients with Eisenmenger syndrome compared to placebo. However, it was associated with increased clinical composite outcomes, primarily driven by worsening heart failure.

## 23. Hemoptysis in Eisenmenger Syndrome: Identifying and Treating a Cause Reduces Recurrences

Sivasubramanian R^1^, Kadiyani L^1^, Gupta S^1^, Joseph J^1^, Jagia P^1^, Kumar S^1^, Kothari S^1^, Saxena A^1^, Sharma S^1^

^1^AIIMS New Delhi, New Delhi, India

**Background:** Hemoptysis is a common cause of morbidity and mortality in Eisenmenger syndrome (ES). However, there is a paucity of studies systematically evaluating ES patients with hemoptysis for a potentially treatable underlying cause. We prospectively analyzed the clinical predictors and causes of hemoptysis in a cohort of patients with ES using computerized tomographic pulmonary angiography (CTPA).

**Methods:** We prospectively studied 95 ES patients who presented consecutively to our center. Patients with hemoptysis underwent a CTPA within two weeks of the index bleeding to search for potentially treatable causes.

**Results:** Hemoptysis was present in 38 (40%) patients at presentation or on follow‐up. There were no significant differences between patients with and without hemoptysis regarding age, functional class, baseline oxygen saturation, or complexity of congenital heart disease. Patients with hemoptysis had significantly lower 6‐minute walk distances than those without hemoptysis (356 m versus 435 m; p = 0.001). CECT revealed a treatable cause of hemoptysis in nearly half the (18 patients) with hemoptysis. The causes included aortopulmonary collaterals (29%), bronchial collaterals (2.6%), pulmonary artery thrombosis (5.2%), pulmonary tuberculosis (7.8%), and pulmonary artery pseudoaneurysm (2.6%). Appropriate treatment of such etiologies reduced hemoptysis recurrences (OR 0.46; 95% CI 0.28 – 0.64) over follow‐up.

**Conclusion:** Hemoptysis is frequent in Eisenmenger syndrome patients, and a CECT can identify a treatable lesion in about half of them. Etiology‐specific treatment of hemoptysis results in fewer recurrences.

## 24. Postoperative Residual PAH in a Child With ASD ‐ A Diagnostic Odyssey

Sivasubramanian R^1^, Kadiyani L^1^, Krishnaswamy S^1^, Arumugam P^1^, Arava S^1^, Jagia P^1^

^1^AIIMS New Delhi, New Delhi, India

This is a case report of a 9‐year‐old child with recurrent episodes of hemoptysis who was initially diagnosed with an atrial septal defect (ASD). She underwent surgical patch closure of ASD. Despite surgical intervention, the symptoms persisted, prompting further investigation, which revealed partial anomalous pulmonary venous return and residual pulmonary hypertension. The child was referred to our center for further management. The chest X‐ray showed multiple ill‐defined reticular opacities in bilateral lung fields, relatively sparing upper lobe. High‐resolution computed tomography (HRCT) of the chest revealed diffuse ground glass opacities and septal lines, raising suspicion for pulmonary capillary hemangiomatosis (PCH) and pulmonary veno‐occlusive disease (PVOD). Subsequent lung biopsy confirmed the diagnosis of PVOD, a rare and often misdiagnosed cause of pulmonary hypertension in children.

**Conclusions:** PCH‐PVOD is one of the rarer entities, especially in children, that results in PAH, which is often misdiagnosed as idiopathic PAH. It is difficult to differentiate between PCH and PVOD as they both have similar clinical, radiological, and histological findings and can challenge clinicians' diagnostic skills. The occurrence of PCH‐PVOD in the setting of congenital heart disease led to a diagnostic challenge.

## 25. 17β‐Estradiol Enhances Right Ventricle Angiogenesis and Endothelial Cell Fatty Acid Oxidation in PAH in a Carnitine Palmitoyltransferase 1‐Dependent Manner

Bousseau S^1^, Walts A^1^, Frump A^2^, Nemkov T^3^, D'Alessandro A^3^, Provencher S^4^, Breuils‐Bonnet S^4^, Pullamsetti S^5^, Bonnet S^4^, Suresh K^6^, Lahm T^1,3,7^

^1^National Jewish Health, Denver, USA, ^2^Indiana University School of Medicine, Indianapolis, USA, ^3^University of Colorado Anschutz Medical Campus, Aurora, USA, ^4^Laval University, Québec city, Canada, ^5^Max Planck Institute for Heart and Lung Research, Bad Nauheim, Germany, ^6^Johns Hopkins University School of Medicine, Baltimore, USA, ^7^Rocky Mountain Regional VA Medical Center, Aurora, USA

**Rationale:** Lack of coordinated angiogenesis as well as reductions in fatty acid oxidation (FAO) and mitochondrial metabolism in the right ventricle (RV) promote RV failure development in PAH. 17b‐estradiol (E2), the predominant female sex steroid, improves RV function in PAH, but the mechanisms are incompletely understood. Hypothesis: E2 exerts pro‐angiogenic effects in the RV by promoting FAO in RV endothelial cells (RVECs).

**Methods:** Published human RV RNA‐sequencing data were interrogated for angiogenesis and FAO regulators. Ovariectomized female sugen/hypoxia (SuHx)‐PH rats were treated with E2 (75 mcg/kg/d). RV capillary density was quantified via the capillary/cardiomyocyte ratio, accompanied by RNA‐sequencing with a focus on angiogenesis and FAO regulator transcripts. In vitro, we assessed effects of E2 (0.1‐100 nM; 24 h) on processes that regulate angiogenesis including migration (scratch assay), proliferation (CCK8 assay), tube formation (matrigel assay), and angiogenic marker expression (Western‐blot). We measured endothelial metabolites, focusing on FAO (mass spectrometry, Western‐blot, lipid droplets, FAO activity) and mitochondrial function (MitoSox, mitotracker, Western‐blot). P < 0.05 was considered statistically significant.

**Results:** Angiogenesis and FAO regulator transcripts were reduced in human RV failure. In SuHx‐RVs, E2 prevented capillary rarefaction and increased transcripts of pro‐angiogenic and FAO mediators. In RVECs from PAH patients (PAH‐RVECs), E2, via estrogen receptor‐alpha (ERa), stimulated tube formation. E2, via ERa, increased PAH‐RVEC abundance of carnitine palmitoyltransferase 1 (CPT1), a master FAO regulator. In PAH‐RVECs, E2 prevented PAH‐induced decreases in FAO metabolites, reduced intracellular lipid accumulation, and increased FAO activity. Stimulatory effects of E2 on PAH‐RVEC tube formation and angiogenic mediator expression were CPT1‐dependent. E2 also reduced mitochondrial ROS production and expanded the mitochondrial network in vitro.

**Conclusion:** E2 stimulates angiogenesis and increases FAO in vivo and in vitro. E2 increases angiogenesis in PAH‐RVECs in an ERa‐ and CPT1‐dependent manner. Harnessing the RVEC E2‐ERa‐CPT1 axis may be an innovative approach to improve RV function in PAH.

## 26. Long‐Covid in Latin America: Insights From the First Regional Study Using Invasive Cardiopulmonary Exercise Testing

Duarte A^1^, Oliveira F^1^, Costa G^1^, Lafetá M^1^, Sperandio P^1^, Moraes M^1^, Menezes T^1^, Santos J^1^, Silva B^1^, Ferreira E^1^, Oliveira R^1^

^1^Federal University of São Paulo, Brazil, São Paulo, Brazil

**Introduction:** More than half a billion people have been infected with SARS‐Cov‐2 worldwide. Up to 20% of survivors have symptoms beyond 12 weeks (i.e. Long‐COVID), including exercise intolerance and fatigue, which impose significant social and economic burdens. One possible mechanism of Long‐COVID is right ventricular (RV) preload dysfunction, characterized by low right atrial pressure (RAP) during exercise. The invasive cardiopulmonary exercise test (iCPET) is the gold standard method for identifying RV preload dysfunction. Objective: Investigate the mechanisms underlying exercise intolerance in Long‐COVID through iCPET.

**Methods:** As part of a larger trial, this prospective substudy included 20 patients with Long‐COVID who had systematically undergone a maximal incremental iCPET at the Federal University of Sao Paulo, Brazil. RV pre‐load dysfunction was diagnosed by a peak RAP ≤ 7 mmHg. Patients also completed three questionnaires: ME/CFS, SAS, and Nijmegen, to evaluate fatigue, dysautonomia, and hyperventilation‐related symptoms.

**Results:** 75% were women, mean age was 48 ± 12 years, and BMI was 28.6 ± 5.3 kg/m2. Dyspnea (mMRC≥ 1) was present in all patients, and fatigue was the second most common symptom. The ME/CFS questionnaire was positive in 14 cases (70%). All patients presented at least one disautonomic symptom and 16 patients (80%) met the hyperventilation criteria through SAS and Nijmegen questionnaires, respectively. At peak exercise, iCPET revealed a low peak RAP (2.5 [0‐5] mmHg), associated with both reduced peak O2 consumption (VO2) (77.5 [55.5‐88.5] % pred) and systemic O2 extraction (EO2) (0.60 ± 0.08). No signs of pulmonary vascular dysfunction were observed.

**Conclusions:** For the first time in Latin America, we describe the occurrence of RV preload dysfunction in patients with Long‐COVID. This condition is associated with a reduced peak VO2 and EO2, and patients’ symptoms. Our ongoing clinical trial will further explore the mechanisms of exercise intolerance in Long‐COVID and its potential treatment approaches (https://ensaiosclinicos.gov.br/rg/RBR-7kyk2wy).

## 27. Impaired Pulmonary and Systemic Vascular Distensibility in Connective Tissue Disease‐Associated Pulmonary Hypertension: Evidence of Systemic Vascular Dysfunction

Menezes T^1^, Lee M^2^, Santos J^1^, Duarte A^1^, Oliveira F^1^, Costa G^1^, Sperandio P^1^, Ota‐Arakaki J^1^, Ferreira E^1^, Singh I^3^, Graham B^2^, Oliveira R^1^

^1^Federal University of São Paulo, Brazil, São Paulo, Brazil, ^2^University of California San Francisco, San Francisco, USA, ^3^Yale School of Medicine, New Haven, USA

**Introduction:** Pulmonary hypertension (PH) is a frequent and severe complication of connective tissue disease (CTD). Exercise intolerance is a cardinal symptom in CTD‐PH and, considering the systemic nature of the disease, might have multifactorial causes. Vascular distensibility (α) is an early marker of vascular dysfunction. While there is evidence that pulmonary α is impaired in PH, little is known about systemic α.

**Objective:** In this study we aim to evaluate the systemic α as an index of systemic vascular dysfunction in relation to pulmonary α in a cohort of CTD‐PH patients.

**Methods:** Systemic and pulmonary α (%/mmHg) were determined from multipoint direct systemic and pulmonary pressure‐flow plots, respectively, during exercise right heart catheterization (RHC). Consecutive patients with confirmed or suspected CTD‐PH referred for exercise RHC at the Federal University of Sao Paulo, Brazil were prospectively recruited.

**Results:** 62 patients were evaluated. 98% (n = 61) of the patients had an abnormal hemodynamic response to exercise and 79% (n = 49) had resting PH. As expected, pulmonary α was reduced in 97% (n = 59) of the patients (median [IQR] = 0.42 [0.24‐0.76] %/mmHg). Interestingly, systemic α was reduced in 86% (n = 53) of the patients (median [IQR] = 0.18 [0.13‐0.22] %/mmHg). Both pulmonary (R2 0.2; 95% CI 0.2‐0.7; p < 0.001) and systemic (R2 0.2; 95% CI 0.2‐0.6; p = 0.002) α had a weak correlation with stroke volume index, and therefore, were not solely flow dependent.

**Conclusion:** In CTD‐PH both pulmonary and systemic α are impaired, suggesting systemic, and not only pulmonary, vascular dysfunction. Systemic α is not entirely explained by blood flow during exercise, indicating that intrinsic mechanisms of systemic vascular disease are present in this population. Therapeutic interventions in CTD‐PH should consider the systemic nature of the vascular dysfunction.

## 28. Peripheral Oxygen Extraction and its Impact on Exercise Tolerance in Connective Tissue Disease‐Associated PH

Oliveira F^1^, Menezes T^1^, Lee M^2^, Santos J^1^, Duarte A^1^, Costa G^1^, Sperandio P^1^, Ota‐Arakaki J^1^, Ferreira E^1^, Graham B^2^, Oliveira R^1^

^1^Federal University of São Paulo, Brazil, São Paulo, Brazil, ^2^University of California San Francisco, San Francisco, USA

**Introduction:** Connective tissue diseases (CTD) are a leading cause of pulmonary hypertension (PH), with elevated morbimortality. Exercise intolerance is a major symptom in CTD‐PH; however, assessing exercise intolerance is challenging and must consider both oxygen delivery and peripheral oxygen extraction (EO2). A better understanding of EO2 could lead to new therapeutic targets to improve CTD‐PH outcomes.

**Objective:** To evaluate the impact of EO2 on exercise tolerance in CTD‐PH patients.

**Methods:** CTD patients referred to exercise right heart catheterization with arterial line and a contemporary cardiopulmonary exercise test (CPET) were prospectively recruited at the Federal University of Sao Paulo, Brazil. EO2 was calculated based on the ratio between the difference in arterial and mixed‐venous oxygen content and the arterial oxygen content at peak exercise.

**Results:** 51 patients were evaluated. 41 (80.4%) had resting PH. EO2 was reduced (< 0.8) in all patients. Two groups emerged: 37 patients (27 resting PH) with a peak EO2 < 0.8 and concordant peak mixed‐venous partial O2 pressure (PvO2) ≥ 27 mmHg, and 14 patients (all resting PH) with peak EO2 < 0.8 and paradoxal PvO2 < 27 mmHg. Patients with PvO2 < 27 mmHg had worse cardiac function compared to patients with PvO2 ≥ 27 mmHg, suggesting a compensatory mechanism for maintaining oxygen consumption (VO2). Cardiac output was on average 3.4 L/min lower (p = 0.007) and stroke volume index was on average 11.8 L/beat lower (p = 0.019) in the group with PvO2 < 27 mmHg. Nonetheless, despite these differences in central cardiac function, NYHA functional class was similar and peak oxygen consumption (VO2) was on average impaired both in those with PvO2 ≥ 27 mmHg and PvO2 < 27 mmHg (73 ± 29 vs. 63 ± 25% pred., p > 0.05).

**Conclusion:** EO2 is impaired in CTD‐PH and impact exercise performance regardless of cardiac function. Therapeutic interventions towards improving exercise tolerance in CTD‐PH should target the peripheral oxygen extraction in addition to the pulmonary vasculature.

## 29. Peak Oxygen Consumption Trajectory in Schistosomiasis‐Associated PAH and Idiopathic PAH: Insights From a Brazilian PAH Referral Center

Santos J^1^, Menezes T^1^, Duarte A^1^, Oliveira F^1^, Costa G^1^, Sperandio P^1^, Ota‐Arakaki J^1^, Ferreira E^1^, Oliveira R^1^

^1^Federal University of São Paulo, Brazil, Sao Paulo, Brazil

**Introduction:** Schistosomiasis (Sch) is a highly prevalent disease worldwide, affecting more than 200 million people. Of these, 5‐10% may present schistosomiasis‐associated pulmonary arterial hypertension (Sch‐PAH), making this one of the main causes of PAH around the globe. Exercise intolerance is a main feature of PAH, negatively impacting oxygen consumption (peak VO2). Despite the well‐established prognostic value of peak VO2 in other PAH etiologies, little is known about its trajectory over time in Sch‐PAH.

**Objectives:** To understand peak VO2 trajectories during follow‐up in Sch‐PAH in relation to idiopathic PAH (IPAH).

**Methods:** This was a retrospective, cross‐sectional, single‐center study. Sch‐PAH and IPAH patients who performed a cardiopulmonary exercise testing (CPET) at baseline and during clinical follow‐up were analyzed.

**Results:** 92 patients were recruited, 75 IPAH and 17 Sch‐PAH. Mean follow time was 6 ± 4 years. 75 (81,5%) were women, and the mean age was 43 ± 14 years. Sch‐PAH showed better peak VO2 at baseline than IPAH (63 ± 19 vs. 51 ± 15% pred., p = 0.006). However, delta peak VO2 between the first and last CPET was similar between Sch‐PAH and IPAH (3 ± 14 vs. 8 ± 16% pred., p = 0.19), even after correcting for time of follow‐up (0 ± 5 vs. 2 ± 5% pred. per year, p = 0.1). Other CPET markers of pulmonary vascular disease such as O2 pulse and VE/VCO2 slope showed similar behavior over time between groups.

**Conclusions:** Sch‐PAH and IPAH have similar peak VO2 trajectories over time, despite the better peak VO2 of Sch‐PAH at baseline. These results challenge the concept that Sch‐PAH has a more benign disease course compared to IPAH. Further studies are necessary to understand the mechanisms of disease progression in Sch‐PAH.

## 30. Evaluating Quality of Life in South American PH Patients With Emphasis‐10

Costa G^1^, Menezes T^1^, Santos J^1^, Duarte A^1^, Oliveira F^1^, Sperandio P^1^, Ota‐Arakaki J^1^, Ferreira E^1^, Oliveira R^1^

^1^Federal University of São Paulo, Brazil, São Paulo, Brazil

**Introduction:** Pulmonary hypertension (PH) has a profound impact on patients' quality of life (QoL), especially among patients with connective tissue disease‐associated PH (CTD‐PH). EmPHasis‐10 is a practical and useful instrument to assess QoL in PH and, to the best of our knowledge, there has been no study using EmPHasis‐10 in South American PH patients.

**Objective:** In this study, we aimed to evaluate the performance of EmPHasis‐10 in measuring QoL in CTD‐PH patients from a South American PH center and to evaluate its correlation with hemodynamic parameters assessed by right heart catheterization (RHC).

**Method:** This was a prospective, cross‐sectional study evaluating CTD‐PH patients referred to RHC at the Federal University of São Paulo, Brazil. All patients answered the EmPhasis‐10 questionnaire immediately before undergoing RHC.

**Results:** 46 CTD‐PH patients were evaluated. The EmPhasis‐10 score positively correlated with the NYHA functional class (Spearman's R 0.6; p < 0.001) and the right atrium pressure (RAP) (Spearman's R 0.3; p < 0.048). There was a trend towards a negative correlation between the EmPHasis‐10 score and stroke volume index (SVi) (Spearman's R ‐0.29; p = 0.05) and pulmonary arterial compliance (PAC) (Spearman's R ‐0.28; p = 0.059).

**Conclusion:** The EmPhasis‐10 score is directly associated with a higher functional class in patients with CTD‐PH from South America. Additionally, EmPHasis‐10 correlates with the hemodynamic severity assessed by RHC, including markers of reduced pulmonary vascular reserve and reduced cardiac function. To the best of our knowledge, this is the first study evaluating the applicability of the EmPHasis‐10 questionnaire in a South American PH cohort.

## 31. Global Treatment Disparities Within High and Middle‐High Income PH Centers: A Cluster Analysis by the PVRI IDDI Access to Care Workstream

Rischard F^1^, Bernardo R, Balasubramanian V, Drumm S, Kantorovich A, Osborn K, Prisco S, Raj J, Sahay S, Liu Y, Golden G

^1^University Of Arizona, Tucson, United States

World Bank low‐income status is used by stakeholders to allocate resources globally, but little is known about resource disparities in high and middle‐high countries. The goal of the PVRI Access to Care Workstream is to assess resource disparities limiting access to care. We used an agnostic approach to uncover PH center disparities among high and middle‐high income centers.

A 36‐question survey was distributed globally by PVRI to PH specialists at high and middle‐high income centers. A distance‐based agglomerative hierarchical cluster analysis was done using survey answers examined for completeness, collinearity, and usefulness based on current guidelines. Optimal number of clusters was determined using the Bayesian Information Criterion. Inter‐cluster differences of candidate variables were determined by ANOVA.

Among the 137 high and middle‐high income centers, 8 were excluded due to missing data. 13 variables were used to define 3 clusters‐ cluster 1 (28/22%), cluster 2 (37/21%), and cluster 3 (74/57%). Clusters were strongly defined by parenteral prostenoids (0%, 70%, and 93%, P = 0.02), riociguat (7%, 67%, 97%, P = 0.0001), center patients > 50 (0%, 79%, 100%, P = 0.0001), proportion of Asian patients (64%, 4%, 13%, P = 0.0001), CTEPH patients > 20 (18%, 0%, 78%, P = 0.0001), and specialty of treating physician (79% cardiology, 40% pediatric cardiology/30% pulmonary, 65% pulmonary, P = 0.03), respectively. Cluster 1 was in East Asia and Pacific regions and had less access to v/q scans (28% vs. > 80%), and health insurance (57% vs > 77%). Cluster 2 was in Latin American/Caribbean (63%) and had less clinical trials (2 versus 6 cluster 3, P = 0.0001). Cluster 3 was European (27%), Latin American (27%), and North American (32%), often White, and 80% insured. Greater than 90% all centers had access to echocardiography and catheterization.

Cluster analysis by high and middle‐high income PH centers revealed large disparities in treatment availability based on region and racial/ethnic composition. Diagnostics were much less affected.

## 32. The Prevalence of Pulmonary Hypertension in Sickle Cell Disease in Sub‐Saharan Africa: A Systematic Review and Meta‐Analysis

Santi A^1^, Wang L^2^, Ngah V^1^, Robbins E^3^, Guoqing D^2^, Maron B^1,4^, Zeder K^1,5^

^1^University of Maryland‐Institute for Health Computing, Bethesda, USA, ^2^Department of Biostatistics and Bioinformatics, George Washington University, Washington DC, USA, ^3^Division of Pulmonary and Critical Care Medicine, University of Maryland School of Medicine, Baltimore, USA, ^4^Department of Medicine, University of Maryland School of Medicine, Baltimore, USA, ^5^Division of Pulmonology, Medical University of Graz, Austria

**Background:** Sickle cell disease (SCD) is a common monogenic disorder and affects populations in sub‐Saharan Africa (SSA) disproportionately. In SCD, hemolytic anemia and episodic vaso‐occlusion lead to pulmonary hypertension (PH), which is an independent predictor of increased morbidity and early mortality. Despite the clinical relevance of PH in SCD patients, the prevalence of PH‐SCD in SSA is not known.

**Aim:** To determine the prevalence of PH‐SCD in SSA.

**Methods:** This is a systematic review following PRISMA and GATHER guidelines searching PubMed (Medline), Embase, Web of Science, and LILACS for original studies for the prevalence of PH‐SCD in 15 pre‐selected SSA countries based on population size and income‐level. Reports published between 1973 (PH first defined) and September 31st, 2023 were eligible for inclusion. Studies without a clear PH definition were excluded. A meta‐analysis was performed using a random‐effects model.

**Results:** In total, we included N = 24 studies published between 2007 and 2023 that comprised 4,397 patients (range of mean age: 7‐28 yr). All studies originated from low‐middle income countries and 83% (N = 20) were from West Africa. The frequency of single‐center, hospital‐based, and prospective study design was 71% (N = 17), 92% (N = 22), and 92% (N = 22), respectively. In all studies, echocardiography was used to diagnose PH. The estimated mean PH prevalence was 15.4% (95%CI: 11.2‐20.1%) when using a systolic pulmonary arterial pressure threshold of 30 mmHg (N = 21 studies) and 8.7% (95%CI: 4.7‐13.8%) when using 35 mmHg (N = 13 studies). Of all studies, 70.8% (N = 17), 16.7% (N = 4) and N = 3 (12.5%) had low, intermediate, and high risk of bias, respectively.

**Conclusion:** Our data demonstrate that PH is common in SCD, and that PH‐SCD is highly prevalent in SSA. These findings warrant prospective studies on the contribution of PH to adverse outcomes in SCD within SSA and suggest public health measures focused on screening at‐risk patients should be considered.

## 33. The Economic Impact of Pulmonary Arterial Hypertension: A Systematic Literature Review

Ramani G^1^, Bali V^2^, Black H^2^, Redwood B^3^, Zile I^3^, Humphries A^3^, Lautsch D^2^

^1^Division of Cardiovascular Medicine, Department of Medicine, University of Maryland School of Medicine, Baltimore, United States, ^2^Merck & Co., Inc., Rahway, United States, ^3^Adelphi Values PROVE, Bollington, United Kingdom

**Objective:** To systematically review published economic literature in pulmonary arterial hypertension (PAH)

**Methods:** An updated review was conducted through Embase, MEDLINE, EconLit, Cochrane Library, and Centre for Reviews and Dissemination for publications (01‐JAN‐2012 – 01‐SEP‐2023) and select conferences for abstracts (01‐JAN‐2020 – 01‐SEP‐2023). Prespecified PICOTS criteria included: adult PAH patients, PAH‐pharmacologic interventions, economic outcomes including direct/indirect costs, resource use, utility estimates, and varied study designs.

**Results:** Within 148 studies total were: 12 economic evaluations, 9 utility studies, and 58 studies reporting healthcare cost and resource utilization (HCRU). Most new therapies were not cost‐effective compared to standard of care or did not present full cost‐effectiveness with ICER. In Canada, Selexipag compared to best supportive care yielded ICERs between $187,418 and $486,421 per QALY, and Macitentan via cost‐minimization analysis, cost $3,873 and $25,878 more annually than Ambrisentan and Bosentan per patient, respectively. In the UK, the mean (SD) time trade‐off utility for oral prostacyclin analog treatments was 0.85 (0.16), and mean disutilities associated with inhaled, subcutaneous and IV administration compared to oral were −0.109 (0.20), −0.262 (0.28), and −0.307 (0.29), respectively. In HCRU studies, PAH patients reported higher economic burden than controls. One US study reported a total average medical cost of $53,923 over 6 months for patients initiated on PAH‐specific therapies.

**Conclusions:** Available literature suggests substantial economic burden associated with PAH, with higher HCRU reported across studies and newer therapies lacking cost‐effectiveness when compared to standard of care.

## 34. Multicenter Prospective Cross‐Sectional Study of Schistosomiasis‐Associated Pulmonary Arterial Hypertension in Brazil

Oliveira R^2^, Correa R^3^, Loureiro C^4^, Sperandio P^2^, Farnese C^3^, Lima R^5^, Lucena J^2^, Siqueira G^2^, Mancuso F^2^, Filho J^2^, Ota‐Arakaki J^2^, Duani H^3^, Souza L^3^, Goes C^3^, Mickael C^6^, Hilton J^1^, Graham B^1^

^1^UCSF, San Francisco, USA, ^2^Federal University of Sao Paulo, Brazil, ^3^Federal University of Minas Gerais, Belo Horizonte, Brazil, ^4^Santa Casa da Bahia, Salvador, Brazil, ^5^Hospital Julia Kubitschek, Belo Horizonte, Brazil, ^6^University of Colorado, Aurora, USA

**Background:** Schistosomiasis (Sch) is a major cause of pulmonary arterial hypertension (PAH) worldwide. Sch‐associated hepatosplenic disease (SchHSD) is likely a disease precursor for schistosomiasis PAH (SchPAH) development. We hypothesized that a cross‐sectional study of subjects with SchPAH versus SchHSD without PH would provide insights into mechanisms of SchPAH pathogenesis.

**Methods:** Right heart catheterization (RHC)‐proven SchPAH subjects and at‐risk SchHSD subjects are being prospectively enrolled at 4 PH expert centers in Brazil. All data are recorded in a REDCap database based at UNIFESP, and blood and urine specimens are banked in a biorepository at UFMG. SchHSD subjects underwent PH screening via echocardiography (echo). Sch infection was screened by urine antigen assay. Study procedures were approved by Institutional Ethical Committees of Brazilian participating centers and UCSF.

**Results:** Among enrollees by month 16 of 36, 45% of 44 SchHSD versus 65% of 23 SchPAH subjects were female. 65% of SchHSD and 50% of SchPAH were functional class 1, and SchPAH median 6MWD was 465 m. 24 SchHSD and all SchPAH subjects underwent echo's; no pericardial effusions were identified. Among SchHSD subjects, the median TRJV was 2.4 m/sec; 4% had TRJV > 2.8 m/sec and 25% had TRJV > 2.5 m/sec. Among SchPAH subjects, median hemodynamic values are RAP = 11 mmHg, mPAP =56 mmHg (range 34‐82), PAWP = 11 mmHg, PVR = 1256 dyn‐sec/cm5, CI = 2.2 L/min/m2, and SvO2 = 63%. One patient had a positive Sch urine antigen test.

**Conclusions:** Interim results show that SchHSD subjects are majority male whereas SchPAH subjects are majority female, potentially due to exposure risk factors for SchHSD versus PAH‐driving biology for PAH. 4% of SchHSD subjects have concern for PH by strict echo guidelines (2.8 m/sec), but prevalence could be 25% using a lower threshold aligned to newer RHC criteria. RHC‐proven SchPAH subjects have significant PAH, with low CI and SvO2. Active schistosomiasis infection is rare in these cohorts.

## 35. Cluster Analysis Identifies Computed Tomography Imaging‐Based Phenotypes of Pulmonary Hypertension Associated With Chronic Obstructive Pulmonary Disease (COPD‐PH)

Johnson S^1^, Pistenmaa C, Vanderpool R, Choi B, Nardelli P, San Estepar R, Washko G, Rahaghi F

^1^Brigham And Women's Hospital, Boston, USA, ^2^Ohio State University, Columbus,

**Background:** Recent PH consensus statements emphasize the importance of chest computed tomography (CT) for PH phenotyping. This is particularly relevant for COPD‐PH where there are no approved therapies as CT analytics may inform phenotypes to guide clinical trial enrollment. We hypothesized that CT measures of emphysema and small vessel volume, a metric correlated with pulmonary artery pressure, would identify imaging‐based COPD‐PH subgroups with differential clinical outcomes and protein expression.

**Methods:** We identified smokers from the COPDGene study with a pulmonary artery to aorta ratio > 1, a non‐invasive marker of PH, and spirometrically confirmed COPD. We applied k‐means cluster analysis to four select variables ‐ FEV1, diffusion capacity, arterial small vascular volume (< 5mm2) to total arterial vascular volume and %emphysema – and performed survival analysis to describe outcomes by cluster. T‐testing evaluated select protein expression by cluster.

**Results:** Three clusters of subjects with presumed COPD‐PH were identified. Cluster 1 (nC1 = 135) had minimal emphysema (4[10]) but loss of small arterial volume or ‘pruning’ (0.45 (0.07)). Cluster 2 (nC2 = 62) had a similar degree of pruning (0.44 (0.08)) but substantial emphysema (45 (14)). Cluster 3 (nC3 = 100) had minimal emphysema and pruning (57(7) and 1[3], respectively). After adjusting for age, compared to reference C2, C0 and C1 experienced a significantly decreased risk of death (HRC0 0.12 (95% CI 0.05, 0.25; p < 0.01); HRC1 0.49 (95% CI 0.32, 0.77, p < 0.01)). PTPRD expression was higher in C2 (vasculopathy in the presence of emphysema) as compared to C1 (vasculopathy in the absence of emphysema) (p < 0.05). PRDX4 expression was higher in C1 as compared to C2 (p = 0.02).

**Conclusion:** Cluster analysis deconstructs CT‐defined COPD‐PH heterogeneity to identify imaging sub‐phenotypes with differential pruning and emphysema as well as all‐cause mortality and COPD‐ and PH‐centric protein expression. In turn, CT is poised to advance COPD‐PH sub‐phenotyping and ongoing efforts to identify effective treatments.

## 36. Progressive Development of Pulmonary Hypertension During Infancy After Antenatal Inflammation in Experimental BPD

Dias Maia P^1,2^, Seedorf G^2^, Gonzalez T^2^, Bye E^2^, Frank B^3^, Mandell E^1,2^, Abman S^2,4^

^1^Section of Neonatology, Department of Pediatrics, University of Colorado Anschutz School of Medicine and Children's Hospital Colorado, Aurora, United States, ^2^Pediatric Heart Lung Center, Department of Pediatrics, University of Colorado Anschutz School of Medicine and Children's Hospital Colorado, Aurora, United States, ^3^Section Cardiology, Department of Pediatrics, University of Colorado Anschutz School of Medicine and Children's Hospital Colorado, Aurora, United States, ^4^Section of Pulmonary Medicine, Department of Pediatrics, University of Colorado Anschutz School of Medicine and Children's Hospital Colorado, Aurora, United States

Antenatal inflammation due to chorioamnionitis is strongly associated with high risk for development of bronchopulmonary dysplasia (BPD) and late pulmonary hypertension (PH) after preterm birth. However, the underlying mechanisms and progressive changes in vascular pathophysiology from birth through infancy remain uncertain.

We hypothesized that early disruption of angiogenesis due to antenatal inflammation impairs lung alveolar and vascular growth, which increases susceptibility for the late development of PH in chorioamnionitis‐induced BPD. Fetal rats were exposed to ETX or saline (controls) 2 days before term (n = 7‐12 per group). We evaluated lung structure by morphometric analysis and pulmonary hemodynamics by transthoracic echocardiogram at postnatal days 2 (D2), D7, and D14.

ETX‐exposed rats exhibited significantly reduced radial alveolar counts (RAC) by 27% at D2 and D7, and by 43% at D14 compared to controls (p < 0.05). Pulmonary vascular density decreased by 31.5% at D2, 46.2% at D7, and 38.6% at D14 (p < 0.05) in the ETX‐exposed groups compared to controls. ETX‐exposed rats showed progressive arterial wall thickening, with vessel wall thickness increasing by 48.5% at D7 and 85.5% at D14 (p < 0.05).

Serial echocardiograms revealed increased interventricular septal flattening at D2 by 25.2%, at D7 by 35.1%, and by 48.1% at D14 in the ETX group compared to controls. At D7, EXT‐exposed pups showed increased right ventricular wall thickness in diastole (RVWTd) by 20.7% compared to controls. At D14, ETX‐exposed pups show an increase in RVWTd by 19.3%, a decrease in pulmonary artery acceleration time to ejection time ratio by 17.8%, and an increase in right ventricular systolic to diastolic duration ratio by 7.9% (p < 0.05).

We conclude that impaired lung vascular growth due to antenatal ETX precedes and is sufficient to cause progressive changes in echocardiogram markers of PH during the first two weeks of life in this model, even in the absence of postnatal injury.

## 37. Patient Reported Experience of Participants in the Artisan Study for PAH

Tomson M^1^, Gunzenhauser D^2^, Daczkowski N^1^, McGovern A^1^, Oroczo L^1^, Clark M^3^, O'Toole B^4^, Derma A^5^, Sista P^1^, Rahaghi F^6^

^1^United Therapeutics, Silver Spring, USA, ^2^Heart & Vascular Clinical Research Office, University of Nebraska Medical Center, Omaha, USA, ^3^Pulmonary Hypertension Clinical Program, University of Rochester Medical Center, Rochester, USA, ^4^Carrilion Clinic, Roanoke, USA, ^5^University of Arizona, Tucson, USA, ^6^United Therapeutics, RTP, USA

**Objective:** The ARTISAN study is an ongoing Phase 4 study evaluating an aggressive ‘treat to pressure’ approach to reduce mean pulmonary pressure (mPAP) using parenteral treprostinil and CardioMEMS as a daily pressure monitoring device. We developed a Patient Questionnaire to capture concerns before and during the study and whether they would recommend participation for other patients considering the study.

**Methods:** Our Patient Questionnaire covered the following areas: their concerns and apprehensions prior to joining the study; concerns during their participation; their activity level compared to the beginning of the study and their recommendation to other participants considering enrollment in the study. The study coordinator at each site provided the Questionnaire to each participant who completed a minimum of 90 days from their baseline visit.

**Results:** Seventeen of 27 (63%) possible Patient Questionnaires were received. Of these, 41%(7/17) respondents expressed some concerns prior to participation in the study, while the majority (88%; 15/17) stated that pre‐teaching and home health visits with a nurse before participation better prepared them and ameliorated their concerns for participation in the study. For questions related to concerns during participation, 71% (12/17) responded that they were able to manage everyday tasks, while the remaining 29% had various levels of difficulty. A majority (82%) indicated that they were able to do more activities during participation. Finally, 100% of respondents recommended participation in the study to other patients considering the option.

**Conclusions:** Our Patient survey results demonstrate that despite initial concerns regarding participation in ARTISAN, their experience with preparation, education and availability of resources prior to and during the study helped them overcome their initial concerns and led them to a unanimous recommendation for others to consider participation in the ARTISAN study.

## 38. Rker‐012, A Novel Modified ActRIIB Ligand Trap, Reversed Vascular Remodeling to Increase Lung Perfusion and Alleviate Experimental Pulmonary Arterial Hypertension

Jain P^1^, Babbs K, Nurse T, Thakre A, Pinkus C, Thirumurthy G, Ishimwe J, Fisher f, Grenha R, Lachey J, Seehra J, Lerner L, Joshi S

^1^Keros Therapeutics, Lexington, USA

**Background:** Pulmonary arterial hypertension (PAH) is a severe cardiopulmonary condition characterized by vascular remodeling, resulting in elevated pulmonary artery pressure, right ventricle (RV) dysfunction and heart failure. RKER‐012 is a modified activin receptor type IIB (ActRIIB) ligand trap that specifically binds and inhibits select TGF‐β ligands associated with PAH, including activins A and B and GDFs 8 and 11. To investigate the hypothesis that RKER‐012 improves vascular remodeling, we evaluated the cardiopulmonary effect of RKER‐012 in an established monocrotaline (MCT)‐induced rat PAH model.

**Methods:** Male Wistar rats were divided into 3 groups: normoxia‐control vehicle‐treated (Nor‐Veh), MCT‐induced (40 mg/kg; sc) vehicle‐treated (MCT‐Veh), and MCT‐induced RKER‐012‐treated (MCT‐RKER‐012) rats. Treatment with vehicle/RKER‐012 (10 mg/kg BIW for 2 weeks; sc) was initiated 2 weeks post‐MCT administration, a timepoint where RV systolic pressure (RVSP) has been shown to be elevated¹ (~50 mmHg). At day 28, vascular remodeling was examined using Microfil® infusion and RVSP, RV hypertrophy, echocardiography were measured.

**Results:** Microfil®‐infused angiograms of MCT‐Veh rats showed reduced pulmonary arterial bed density, lumen diameter and discontinuous microvessels reflecting vascular remodeling. In contrast, treatment with RKER‐012 resulted in a near‐complete restoration of the MCT‐induced reduction in pulmonary arterial bed density and lumen diameter, supporting potential reversal of the vascular remodeling underpinning PAH. This reversal of vascular remodeling was reflected in reduced RVSP (‐44.9%; p < 0.004) and reduction in RV hypertrophy (‐24.3%; p < 0.021) in MCT‐RKER‐012 rats compared to MCT‐Veh. Additionally, echocardiography showed near‐complete amelioration of flattening of septal wall and RV dilation in MCT‐RKER‐012 rats vs. MCT‐Veh.

**Conclusion:** Treatment with RKER‐012 reversed MCT‐induced changes in vascular remodeling RVSP, RV hypertrophy, and RV geometry in an experimental PAH rat model, suggesting a potentially translatable benefit for patients with PAH. These preclinical findings further support rationale for the ongoing Phase 2 trial of KER‐012 in patients with PAH (NCT05975905).

¹PMID:17496210

## 39. Heterogeneous Presentation of Pulmonary Hypertension in Children Born and Living at High Altitude. A Cohort Study From Colombia

Diaz Gongora G^1^, Marquez Garcia A, Diaz C

^1^Universidad Nacional De Colombia, Bogota, Colombia

**Introduction:** Pulmonary hypertension (PH) in children living at high altitudes remains poorly understood, particularly the diverse clinical manifestations influenced by hypobaric hypoxia. Given the physiological stressors at altitudes above 2,500 MASL, this study aims to elucidate the heterogeneous ways in which PH presents in pediatric patients.

**Justification:** This study is crucial given the lack of research on the pediatric population with PH at high altitudes, despite millions living in such regions. Understanding these variations in clinical presentation improves early diagnosis and treatment outcomes.

**Methods:** A retrospective cohort of 95 children diagnosed with PH living at high altitude was analyzed. Data were collected from clinical records, echocardiography, and right heart catheterization, with cases classified according to the time and severity of PH presentation.

**Results:** Different ways of expression of PH were found:

a) Severe pulmonary vascular disease in newborns: (9 Cases)

b) Severe pulmonary vascular disease at early age (No newborns)

c) Severe PH after moving from low to high altitude (4 cases)

d) PH after surgery of CHD (13 cases)

e) Suddent presentation of severe and fatal PH

f) Subacute Infant Mountain Sickness

g) High altitude Pulmonary Edema

**Discussion:** This abstract emphasizes the heterogeneity in PH among children living at high altitudes, which can improve clinical trial design with tailored endpoints, and implementing personalized medicine approaches based on environmental and physiological factors. Designing trials for specific needs of each population will lead to more effective interventions.

**Conclusion:** The varying forms of expression, from severe pulmonary vascular disease in newborns to acute cases triggered by environmental changes, underscore the need for tailored diagnostic and therapeutic approaches. Understanding these different manifestations is essential for early intervention and improving outcomes, in populations exposed to hypobaric hypoxia. Further research is necessary to develop strategies that address this heterogeneity in pediatric populations at high altitude.

## 40. Characterization of Persistent Pulmonary Hypertension of the Newborn at High Altitude, BogotÁ: 2640 Meters Above Sea Level. Comparison With Sea Level

Diaz Gongora G^1^, Pedraza D, Ruiz A, Marquez Garcia A, Barrios H, Torres M, Maza O, Vargas Chaves D, Krishnan U

^1^Universidad Nacional De Colombia, Bogota, Colombia

**Introduction:** PPHN is an important pathology in neonates, resulting in significant morbimortality. In the current era, newer management protocols have improved outcomes for these neonates.

**Justification:** Majority of studies on PPHN has been developed at low altitude ‐ sea level; We aim to compare the characteristics and outcomes of PPHN in high altitude vs. At sea level.

**Patients and Methods:** The retrospective descriptive study was conducted in two maternities of Bogota, located at high altitude, and in two maternities of Barranquilla Colombia and we propose to retrospectively study patients at one NICU in New York (control groups at sea level). Patients with diagnosis of PPHN from January, 2018 to December, 2021 were included. The diagnosis was made based on, clinical findings, RV size, pressure and function and ductal shunt by echocardiogram, and measures of BNP or NT‐ProBNP. Gestational age, frequency, age at diagnosis, severity of elevation of pulmonary pressure, perinatal hypoxia, treatment and clinical evolution were analyzed

**Results:** In 4 years we found 154 neonates with PPHN equivalent to a frequency of 3.96/1000 live newborns (double of published data: 2.0/1000 live newborns at sea level). The age at diagnosis was at a median of 48 hours (IQR: 24‐72). Most patients had severe PPHN with a median of PSP of 62 mm Hg (IQR: 54‐70 mmHg). There was a positive correlation between the age at diagnosis and the time to resolution of PH (CI 0.2247, p = 0.0096).

**Conclusions:** At high altitude, the frequency of PPHN was around double of published data at sea level and most patients have severe PPHN. Based on the findings related with the importance of early detection of PPHN, we propose to screen neonates for PPHN if they have near systemic RV pressures with clinical findings of PPHN and elevated BNP or NT‐ProBNP.

## 41. The Role of Mitochondrial Uncoupling Protein 2 in Hypoxia‐ And Cigarette Smoke‐Induced Pulmonary Hypertension

Balasubramanian Lakshmi V^1^, Pak O^1^, Kojonazarov B^1^, Kraut S^1^, Seeger W^1,2,3^, Weissmann N^1^, Sommer N^1^, Garcia Castro C^1^

^1^Excellence Cluster Cardio‐Pulmonary Institute (CPI), Universities of Giessen and Marburg Lung Center (UGMLC), Member of the German Center for Lung Research (DZL), Justus‐Liebig University of Giessen, Giessen, Germany, ^2^Max Planck Institute for Heart and Lung Research, Bad Nauheim, Germany, ^3^Institute for Lung Health (ILH), Giessen, Germany

**Introduction:** Mitochondria are key players in the development of pulmonary hypertension (PH). Previously, we demonstrated that mice deficient in mitochondrial uncoupling protein 2 (Ucp2‐/‐) spontaneously develop mild PH. However, the exact cellular mechanisms underlying the effects of Ucp2 deletion in the pulmonary vasculature during PH remain unclear. To address this, we compared the development of PH in Ucp2‐/‐ mice using two experimental models of PH: chronic exposure to hypoxia and cigarette smoke (CS).

**Methods and Results:** Mice with global Ucp2‐/‐ exhibited mild PH and pulmonary vascular remodeling under basal conditions compared to wild‐type (WT) mice, consistent with previous findings. Additionally, smooth muscle cell (SMC)‐specific deletion of Ucp2 led to mild PH, as assessed by in vivo hemodynamics, echocardiography, and histological analysis. Mechanistically, Ucp2‐/‐ pulmonary artery smooth muscle cells (PASMCs) displayed hyperproliferation in normoxia with enhanced glycolysis, although mitochondrial respiration remained unchanged. This hyperproliferation was partially reduced by increasing glucose oxidation with dichloroacetate. After chronic hypoxic exposure (4 weeks, 10% O2), both Ucp2‐/‐ mice and those with SMC‐specific Ucp2 deletion developed similar levels of PH compared to WT mice. In contrast, after chronic exposure to CS (8 months, 6 hours/day, 5 days/week), Ucp2‐/‐ mice exhibited less severe PH than WT mice. Interestingly, Ucp2‐/‐ mice also showed attenuated emphysema development, as assessed in vivo and through stereological analysis, compared to WT mice.

**Conclusion:** Ucp2 downregulation in PASMCs contributes to the spontaneous development of PH via metabolic alterations. However, Ucp2 appears to play distinct roles in chronic hypoxia‐induced PH and CS‐induced PH, indicating that these pathologies may involve divergent cellular mechanisms.

## 42. Smyd2 And Its Contribution to Right Ventricular Hypertrophy: A Molecular and Therapeutic Perspective

Veeroju S^1^, Kojonazarov B^1,2^, Mamazhakypov A^1^, Sydykov A^1^, Ashir A^1^, Rai N^1^, Breuils‐Bonnet S^3^, Pilz C^1^, Wilhelm J^1^, Guenther S^4^, Ardeschir Ghofrani H^1^, Weissmann N^1^, Braun T^4^, Provencher S^3^, Bonnet S^3^, Seeger W^1,2,4^, Novoyatleva T^1^, Schermuly R^1^

^1^Excellence Cluster Cardiopulmonary Institute (CPI), Department of Internal Medicine, Justus‐Liebig University, Member of the German Center for Lung Research (DZL), Giessen, Germany, ^2^Institute for Lung Health, Giessen, Germany, ^3^Pulmonary Hypertension and Vascular Biology Research Group,Institut Universitaire de Cardiologie et de Pneumologie de Québec, Québec, Canada, ^4^Max Planck Institute for Heart and Lung Research, Bad Nauheim, Germany

Pulmonary arterial hypertension (PAH) often leads to right ventricular (RV) failure, a major cause of mortality. Our study identifies downregulation of HIF‐1α and lysine methyltransferase Smyd2 in decompensated RVs of PAH patients and mice with RV hypertrophy (RVH). Smyd2 expression significantly correlated with Tricuspid Annular Plane Systolic Excursion (TAPSE) in decompensated RVH patients. Hypoxia induced Smyd2 expression in neonatal rat cardiomyocytes (CMs) and cardiac microvascular endothelial cells (CMECs), while Smyd2 silencing reduced HIF‐1α levels under hypoxia.In silico analysis identified a methylation site (K94) on the HIF‐1α protein, and mutant studies confirmed Smyd2‐specific methylation of HIF‐1α at K94, facilitating its nuclear translocation and stabilization under hypoxia. Smyd2 silencing blocked HIF‐1α methylation and stabilization, while conditioned media from Smyd2‐overexpressing CMs enhanced angiogenesis in vitro. Therapeutically, AAV‐mediated Smyd2 overexpression in a pulmonary artery banding (PAB) mouse model improved RV function, as evidenced by enhanced TAPSE, cardiac index, and stroke volume index. Smyd2 overexpression also attenuated RV fibrosis and cardiomyocyte hypertrophy while maintaining capillary density. Additionally, plasma levels of anti‐angiogenic factors, including PAI‐1, TSP‐2, SDF‐1, ANG, and PTX3 were significantly reduced. RNA sequencing revealed decreased glycolytic pathways and upregulation of cardioprotective genes such as Smad6, Nrg1, Ncam1, and Fgfr2 in Smyd2‐overexpressing RVs.

Our findings identify Smyd2 as a key regulator of HIF‐1α methylation and stabilization, suggesting its potential as a therapeutic target for preventing right ventricular failure in PAH.

## 43. Lung De/Recellularization: Unmasking Matrix Cues in Pulmonary Hypertension

Rai N^1^, Feichtenschlager V^1^, Schermuly R^1^

^1^Universities of Giessen and Marburg Lung Center (UGMLC), Excellence Cluster Cardio‐Pulmonary Institute (CPI), Member of the German Center for Lung Research (DZL), Giessen, Germany

**Introduction:** Extracellular matrix (ECM) remodeling with increased collagen deposition, crosslinking of collagen fibers, and a dysfunctional elastic layer are one of the main characteristics of pulmonary arterial hypertension (PAH). Decellularized (DC) tissue scaffolds retain ECM proteins and provide structural support and biochemical cues for cell attachment, proliferation, migration, and apoptosis. Endothelial progenitor cells (CD34 + ) are known to be involved in the pathogenesis of PAH. We aim to utilize de‐ and recellularization to elucidate the ECM effect on human endothelial progenitor cells (EPCs) in the human PAH lungs and murine model of PAH.

**Results:** Decellularization decreased the genomic DNA content in lungs, indicating effective removal of cells. H&E staining showed the maintained lung architecture with absence of cellular components in native and DC lung scaffolds. Immunofluorescence staining revealed preserved ECM proteins in DC mouse and human lungs. Furthermore, mass spectrometry‐based proteomic analysis of decellularized lung scaffold revealed increased core matrisome, proteoglycans and matrix‐bound proteins in hypoxic decellularized lung scaffold compared to control. The phenotype of recellularized EPCs in decellularized scaffold was altered, becoming elongated, and aligned along the vessels over the course of 21 days. We observed higher proliferation of recellularized EPCs on day 14 in hypoxic scaffold compared to control. TUNEL assay revealed a higher number of apoptotic EPCs, recellularized in the control lungs as compared to the recellularized EPCs in the hypoxic scaffold on day 3.

**Conclusion:** ECM alterations in PAH have an impact on the gene expression signature and cellular heterogenity. Elucidating the effect of matrisome from human and murine lungs on cellular phenotype and differentiation would provide insight into matrix‐cell interplay during disease progression and pathogenesis. Thus, the respective pharmacological modulation of ECM as well as endothelial cells in different ex‐vivo and in‐vivo models will lead to novel pulmonary hypertension therapies.

## 44. Hippo/Rhoa Signaling Mediates Nuclear Architectural Alteration via Actin Cytoskeletal Remodeling, a Novel Molecular Axis Driving Pulmonary Hypertension

Olapoju S^1^, Chelladurai P^1^, Cherian A^1^, Lemay S^2^, Goncharov D^4^, Hesami G^1^, Bartkuhn M^1^, Savai R^1^, Seeger W^1^, Kurakula B^3^, Bonnet S^2^, Goncharova E^4^, Pullamsetti S^1^

^1^Universities of Giessen and Marburg Lung Centre (UGMLC), Institute for Lung Health (ILH), Cardio‐Pulmonary Institute (CPI), Member of the German Centre for Lung Research (DZL), Giessen, Germany, ^2^Department of Medicine, Faculty of Medicine, Université Laval, Quebec City, Canada, ^3^Department of Physiology, Amsterdam University Medical Center, Amsterdam, The Netherlands, ^4^Lung Center, Division of Pulmonary, University of California, Davis School of Medicine, USA

Pulmonary hypertension (PH) is a fatal disease characterized by hyperproliferation and increased apoptosis resistance of pulmonary vascular cells. Due to limited treatment options, deciphering novel signaling axis that can be modulated as a therapeutic strategy is of utmost interest.

The cellular nuclear compartment is a key organelle that controls numerous biological processes. Therefore, it is important that the structural integrity of the nucleus is maintained to ensure optimal function. Unfortunately, the structural integrity of the nucleus can be compromised or altered by cellular stress or by mechanosensors, a concept known in various pathological conditions, but has not yet been explored in pulmonary hypertension.

We have observed changes in actin structure and nuclear integrity in IPAH patients compared to healthy controls. Confocal microscopy revealed a significant loss of nuclear circularity, nuclear volume and nuclear shrinkage in the smooth muscle compartment of lung tissue from IPAH patients, with the same trend observed in IPAH PASMCs.

To mimic actin cytoskeletal remodeling and nuclear change in vitro, we stimulated healthy human PASMCs with a RhoA activator (lysophosphatidic acid), as RhoA is known to control mechanosensing that affects actin remodeling and nuclear structure.

Our results show alterations in nuclear integrity, actin structure and dysregulation of Hippo signaling molecules that resemble the phenotype observed in PAH patients.

To reverse this phenotype, we used siRNA and MST1/2‐specific inhibitors (XMU/MP/1) to disrupt the expression of MST1/2, the upstream kinases of Hippo signaling. Interestingly, both strategies reversed the PAH‐like phenotype and suppressed RhoA/LPA signaling upon treatment.

Daily intraperitoneal administration of XMU‐MP‐1 reduced hemodynamic changes, improved cardiac functions, and decreased clinical markers of RV failure in MCT‐induced PH.

In summary, our data demonstrate that nuclear architecture alteration is a central feature of PAH and can be modulated via the RhoA/MST1/2 signaling axis. This represents a potential therapeutic option in PH.

## 45. Endotyping Pulmonary Hypertension in COPD: Possible Role of Systemic and Lung Iron Status

Myronenko O^1^, Curcic P^1^, Douschan P^1^, Zeder K^1,2^, John T^1^, Hoetzenecker K^3^, Suessner S^4^, Kovacs G^1^, Olschewski A^1^, Olschewski H^1,5^, Foris V^1,6^

^1^Medical University Of Graz, Graz, Austria, ^2^Institute for Health Computing, University of Maryland, Bethesda, USA, ^3^Medical University Of Vienna, Vienna, Austria, ^4^Austrian Red Cross, Linz, Austria, ^5^ERN Lung, ORLD Core Network, Frankfurt, Germany, ^6^ Brigham and Women´s Hospital, Harvard Medical School, Boston, USA

**Background:** Chronic obstructive pulmonary disease (COPD) is a global health challenge frequently associated with mild pulmonary hypertension (PH). However, some patients develop severe PH with a very poor prognosis. The underlying factors of different PH severity are unknown. Notably, the lungs of COPD patients contain higher iron levels than those of healthy controls, yet up to 50% of patients develop anemic or non‐anemic iron deficiency associated with more severe PH. Therefore, we aimed to characterize iron status and associated markers in COPD patients with and without severe PH.

**Methods:** In a cohort of 20 lung transplanted COPD patients (LTX) without PH (n = 6), with mild‐to‐moderate PH (n = 7), or with severe PH (mPAP> 35 mmHg, n = 7), serum total iron levels, key iron‐related factors (ferritin, transferrin, and soluble transferrin receptor‐1 (sTfR)), along with ferric iron‐containing cells in the respective lung sections were assessed. Bronchial gene expression was assessed via microarray in donor and COPD lungs with moderate and severe PH (n = 15). An independent cohort of COPD‐PH outpatients (n = 79) was analyzed for systemic factors differentially regulated in the LTX cohort.

**Results:** Across LTX subgroups, systemic iron levels and associated factors were similar, except for sTfR, which was reduced in severe PH vs. no PH (p = 0.029). In lung tissues, the number of iron‐loaded cells was negatively correlated with mPAP (r = 0.636; p = 0.019). In severe PH but not moderate PH, compared to controls, bronchial genes related to iron binding (including FTL, ferritin light chain) were downregulated. In COPD‐PH outpatients, sTfR was correlated with mPAP (r = 0.45; p < 0.001) and PVR (r = 0.395; p < 0.001) and was associated with poor survival (p = 0.002).

**Conclusions:** sTfR may serve as a biomarker for severe PH in COPD outpatients but not end‐stage LTX patients. Iron deposition in the lungs may affect mPAP levels, while downregulation of bronchial iron‐binding genes may be associated with severe PH in COPD.

## 46. Effects of Dapagliflozin on Pulmonary Hypertension in a Rat Pulmonary Vein Banding (PVB) Model

Blumrich L^1^, Muenks J^1^, Mamazhakypov A^1^, Pilz C^1^, Schermuly R^1^, Sydykov A^1^

^1^Cardio‐Pulmonary Institute (cpi), Giessen, Germany

**Introduction:** Left sided congestive heart failure (HF) is the most common type of HF and pulmonary hypertension (PH) due to left heart disease (PH‐LHD) is the most prevalent form of PH. However, there are currently no approved therapeutic strategies available for PH‐LHD. In recent years, several SGLT‐2 inhibitors have shown promise as a novel treatment option for HF. The effects on SGLT‐2 inhibitors on PH‐LHD remain unexplored. For this aim, we utilized our recently developed PH‐LHD model of pulmonary vein banding (PVB).

**Methods:** Wistar‐Kyoto rats underwent either partial occlusion of the left pulmonary veins to induce pulmonary venous congestion (n = 18) or sham surgery (n = 18). After 20 days post op, the animals were treated orally with either placebo or 10 mg/kg BW dapagliflozin, a SGLT‐2 inhibitor, for 4 weeks. Echocardiography and cardiac catheterization were used for in vivo cardiopulmonary phenotyping. Vascular remodelling (wall thickness, degree of muscularisation) was assessed in paraffin‐embedded lung sections.

**Results:** Treatment with dapagliflozin had no negative effects on body weight. Dapagliflozin decreased wall thickness in small pulmonary vessels compared to placebo treatment. These beneficial changes in pulmonary vessels were accompanied by reduced right ventricular hypertrophy.

**Conclusion:** These Data demonstrate that dapagliflozin has a direct effect on the pulmonary vasculature in PH‐LHD.

## 47. Early Recruitment and Alternative Activation of Macrophages Initiate Pathophysiological Changes in Pulmonary Vasculature Upon Smoke Exposure

Sharma V^1^, Loku E^1^, Hadzic S^1^, Kowalski T^2^, Bartkuhn M^2,3^, Seeger W^1,2,4^, Sommer N^1^, Weissmann N^1^, Gredic M^1^

^1^Cardio‐Pulmonary Institute (CPI), Universities of Giessen and Marburg Lung Center (UGMLC), Member of the German Center for Lung Research (DZL), Gießen, Germany, ^2^Institute for Lung Health (ILH), Justus‐Liebig‐University, Giessen, Germany, Gießen, Germany, ^3^Biomedical Informatics and Systems Medicine Science Unit for Basic and Clinical Medicine Justus Liebig University Giessen, Giessen, Germany, Gießen, Germany, ^4^Max Planck Institute for Heart and Lung Research, Bad Nauheim, Germany, Gießen, Germany

**Rationale:** Pulmonary hypertension (PH) often develops in patients with chronic obstructive pulmonary disease (COPD), increasing mortality and morbidity. We previously showed that in smoke‐exposed lungs, bone marrow‐derived macrophages (BMDM) communicate with adjacent pulmonary artery smooth muscle cells (PASMCs) to boost ERK phosphorylation and PASMCs proliferation, ultimately leading to PH in COPD. However, the early cellular and molecular changes leading to the development of pulmonary vascular pathology in COPD remain elusive. In this study, we aimed to explore initial pulmonary vascular changes that contribute to PH in COPD.

**Methods:** C57BL/6 J mice were exposed to CS or room air for 1‐4 weeks and 8 months. We used bulk RNA sequencing, immunostaining, and qPCR to assess alterations in cellular signaling.

**Results:** Immunostaining of the lung sections from mice exposed to CS for 1‐4 weeks demonstrated that accumulation of perivascular macrophages are increased after 3 weeks, while upregulation of ERK pathway and proliferation of pulmonary vascular cells are detectable 1 week later. RNA sequencing revealed the upregulation of several genes associated with the M2‐like phenotype and oxidative phosphorylation pathways in both BM‐derived monocytes (BMMo) and interstitial macrophages (IM) after smoke exposure. Additionally, CS induced distinct transcriptomic changes in lung IM, including the upregulation of cellular pathways known to contribute to tissue remodeling.

**Conclusions:** Overall, our data suggest that CS exposure may trigger monocyte activation and promote their infiltration into the lungs by altering gene expression in BMMo, but also induce further transcriptomic changes in mature recruited macrophages found in the lungs, thus potentiating the pathological crosstalk with PASMCs in situ. CS‐induced accumulation of macrophages in the perivascular space is detectable shortly before the increase in proliferation of pulmonary vascular cells and could initiate this process through the release of soluble factors that activate ERK pathway.

## 48. Pulmonary Vascular Phenotype in COPD: Is There a Transcriptomic Signature in Lung Tissue?

Myronenko O^1^, Foris V^1,2^, Nagaraj C^1^, Wilhelm J^3^, Hoetzenecker K^4^, Olschewski A^1^, Olschewski H^1,5^

^1^Medical University Of Graz, Graz, Austria, ^2^Brigham and Women´s Hospital, Harvard Medical School, Boston, USA, ^3^Justus Liebig University Giessen, Giessen, Germany, ^4^Medical University of Vienna, Vienna, Austria, ^5^ERN Lung, ORLD Core Network, Frankfurt, Germany

**Background:** Chronic obstructive pulmonary disease (COPD) is characterized by airway obstruction, lung parenchymal destruction and pulmonary vascular remodeling. However, between COPD patients, the pathologic involvement of these compartments is very different. The pulmonary vascular phenotype refers to COPD patients with severe pulmonary hypertension (PH) and relatively preserved lung function, and is associated with poor prognosis. To better understand this phenotype, we examined the transcriptomic landscape of lung parenchyma in explanted COPD lungs in the framework of the Vienna lung transplant program.

**Methods:** Bulk RNA sequencing was performed on the explanted lung homogenates from 16 COPD patients, grouped into i) vascular phenotype, ii) severe COPD with severe PH and iii) severe COPD with no/mild/moderate PH. Patients were matched for age and sex. Differential gene expression, gene enrichment and regression analyses were conducted using the R packages. Genes with an adjusted p‐value < 0.05 and a log2 fold change > 1.5 were considered to be significantly differentially expressed (DEGs).

**Results:** In groups i) and ii), compared to group iii), many gene signatures were shared, indicating common pathways associated with severe PH: in particular, inositol lipid‐mediating signaling and IL‐17 signaling pathway. In addition, in groups i) and ii), the NR3C1, glucocorticoid receptor gene, was negatively associated with mPAP (FDR = 0.024). However, in each group, distinct DEGs were also identified. Surfactant homeostasis genes were upregulated in group ii), while in group i), genes related to endothelial cell migration and carbon dioxide transport were upregulated, which may explain more prominent capillary loss, hypoxemia and hypocapnia. Finally, in group i), genes related to B cell receptor signaling, B cell proliferation and hematopoietic cell lineage were upregulated, as compared to group ii).

**Conclusion:** At the parenchymal level, the vascular phenotype of COPD presents with a unique gene expression pattern suggesting a distinct pathological mechanism.

## 49. Lipidomic Signatures as Diagnostic and Prognostic Biomarkers in Pulmonary Hypertension

Olschewski A^1^, Bordag N^10^, Nagy B^2^, Zügner E^4^, Ludwig H^5^, Foris V^11^, Nagaraj C^2,11^, Biaisn V^12^, Kovacs G^11^, Bodenhofer U^5^, Magnes C^4^, Maron B^6^, Ulrich S^7^, Lange T^8,9^, Hötzenecker K^13^, Pieber T^3,14^, Olschewski H^11^

^1^Experimental Anaesthesiology, Department of Anaesthesiology and Intensive Care Medicine, Medical University of Graz, Graz, Austria, ^2^Ludwig Boltzmann Institute for Lung Vascular Research, Graz, Austria, ^3^CBmed GmbH, Center for Biomarker Research in Medicine, Graz, Austria, ^4^Institute for Biomedical Research and Technologies (HEALTH), Joanneum Research Forschungsgesellschaft m.b.H, Graz, Graz, ^5^School of Informatics, Communications, and Media, University of Applied Sciences Upper Austria, Hagenberg, Austria, ^6^University of Maryland School of Medicine, Baltimore, MD and The University of Maryland‐Institute for Health Computing, Bethesda, USA, ^7^Clinic of Pulmonology, University and University Hospital of Zurich, Zürich, Schwitzerland, ^8^Department of Internal Medicine II, Pulmonology and Critical Care, Kreisklinik Bad Reichenhall, Bad Reihenhall, Germany, ^9^Faculty of Medicine, University of Regensburg, Regensburg, Germany, ^10^Department of Dermatology and Venereology, Medical University of Graz, Graz, Austria, ^11^Division of Pulmonology, Department of Internal Medicine, Medical University of Graz, Graz, Austria, ^12^Division of Physiology, Otto Loewi Research Centre, Medical University of Graz, Graz, Austria, ^13^Department of Thoracic Surgery, Medical University of Vienna, Vienna, Austria, ^14^Division of Endocrinology and Diabetology, Department of Internal Medicine, Medical University of Graz, Graz, Austria

Pulmonary hypertension (PH) is characterized by high morbidity and mortality, but early diagnosis is difficult. Less invasive diagnostics and better prognostic markers could significantly improve patient management. We employed a comprehensive metabolomics approach combined with machine learning and specific free fatty acid (FFA)/lipid ratios to identify novel PH diagnostic biomarkers with prognostic properties.

**Methods:** We conducted an exploratory analysis of a broad spectrum of metabolites in PH patients, healthy controls, and disease controls, using both a training and a validation cohort, along with in vitro studies on human pulmonary arteries (PA). High‐resolution mass spectrometry was performed on 233 subjects, followed by machine learning analysis. Additionally, we conducted histologic and gene expression studies focused on lipid metabolism in the PAs of idiopathic pulmonary arterial hypertension (IPAH) lungs and assessed the acute effects of extrinsic FFAs on PA smooth muscle and endothelial cells.

**Results:** The training cohort included 74 PH patients, 30 disease controls, and 65 healthy controls, with an independent validation cohort of 64 subjects. Machine learning demonstrated high diagnostic potential for PH. We identified FFA/lipid ratios with exceptional diagnostic accuracy (AUC 0.89 in the training cohort, 0.90 in the validation cohort), outperforming machine learning. These ratios were also prognostic, significantly enhancing established clinical scores (COMPERA2.0 and FPHR‐noninvasive) for mortality risk. IPAH lungs showed lipid accumulation and dysregulation of lipid homeostasis‐related genes. In PA smooth muscle and endothelial cells, extrinsic FFAs induced excessive proliferation and barrier dysfunction, respectively.

**Conclusion:** Our metabolomics approach reveals lipidic alterations in PH that are suitable for non‐invasive PH diagnosis and simultaneously improve prognostic assessment, complementing established clinical scores. These “lipidic” changes likely reflect the pathological processes in the pulmonary arteries of PH patients.

## 50. Genetic Variants Of Group 1 Paediatric Pulmonary Arterial Hypertension

Juaneda E^1,3^, Damonte V^1^, Larovere L^1^, Grosso C^1^, Silvera S^1^, Savi S^2^, Pabletich F^3^, Diaz J^3^, Juaneda I^1,3^, Peirone A^1,3^, Nicola J^2^

^1^Children´s Hospital, Córdoba, Argentina, ^2^Faculty of Chemical Sciences, National University of Córdoba, Córdoba, Argentina, ^3^Private University Hospital of Córdoba, Córdoba, Argentina

**Introduction:** Pulmonary arterial hypertension (PAH) is a progressive vascular disease with increased rigth ventricular afterload that conducts to heart faillure. Pulmonary arterioles endotelial and muscular cells molecular signals may be altered when genetic variants are present. Objective: Describe genetic variants of group 1 paediatric PAH.

**Material and Methods:** Between February 2019‐March 2024, 14 children had confirmed PAH group, through screening phenotype: paediatric functional class (FC), physical examination, 2D‐Doppler Echocardiography, laboratory and elective cardiac catheterization (CC) After signed informed consent for genetic study, blood samples (biobank 78,5%) for ADN exome PAH pannel was performed.

**Results:** PAH onset age 5.2 ± 5.2 years, 8 female, 6 males, paediatric FC: II:2, IIIa:3 and IIIb:9 patients and phenotype: idiopathic 9, associated to congenital heart disease 3 and persistent fetal circulation of newborn in 2 patients (1 with coincidental secundum atrial septal defect). There was no family history of PAH and comorbidities were present in 2 patients. Genetic variants were present in 5/14 (35,7%): KDR, ATP13A, AQP1, GGCX and NFU1; recognized in 4 patients; unrecognized NFU1 variant with multiple mitochondrial dysfunction syndrome in 1 patient. CC mPAP: 57 ± 14.8 mmHg, PVR 15.5 ± 8.8 mmHg and wedge pressure < 15 mmHg in all. Specific PAH treatment was stablished according risk determinants in all patients through current therapeutic targets. Survival 9/14 (64%), 4 of them > 10 years follow‐up.

**Conclusion:** Genetic variants of paediatric onset PAH were found in 35,7% of this cohort. Pulmonary arterioles endotelial and muscular cells molecular altered signals were described on literature and explains pathophisiology.

## 51. Hypoxia‐Mediated Translation Control in the Lungs – “lncRNA”, the New Kid in the Block?

Msheik Z, Valasarajan C^1,2^, Khassafi F^1,2^, Cherian A^1,2^, Bartkuhn M^2,3^, Savai R^1,2^, Seeger W^1,2^, Pullamsetti S^1,2^

^1^Department of Lung Development and Remodeling, Max Planck Institute for Heart and Lung Research, Bad Nauheim, Germany, ^2^Universities of Giessen and Marburg Lung Center (UGMLC), Institute for Lung Health (ILH), Cardio‐Pulmonary Institute (CPI), Member of the German Center for Lung Research (DZL), Giessen, Germany, ^3^Biomedical Informatics and Systems Medicine, Justus Liebig University, Giessen, Germany

Hypoxia, a condition characterized by insufficient oxygen levels in the tissues, plays a crucial role in various pathological events in the lungs. Recent studies have shown that in addition to coding genes, several long non‐coding RNAs (lncRNAs) are dysregulated under hypoxia. Notably, a few recent studies demonstrated that most lncRNAs have short open reading frames hence the potential to encode functional micro‐peptides. In this project, we aim to delineate the role of lncRNAs with high coding potential in modulating the hypoxia‐driven signaling pathways and their contribution to the pathogenesis of chronic lung diseases. Screening for dysregulated lncRNAs after 24 hours of hypoxia (hox) exposure in human pulmonary arterial smooth muscle cells (hPASMCs) identified several candidates. Among the top 10 most significantly upregulated lncRNAs, LNC16 and LNC19 exhibited a notable coding potential, suggesting their possible translation into micro‐peptides. This was further validated by generating antibodies against the endogenous peptides. Screening of the selected lncRNAs in different lung diseases revealed that LNC16 was significantly upregulated in COPD, while LNC19 was significantly upregulated in both COPD and IPF. Further investigation of the cellular localization of LNC16 and LNC19 revealed that LNC16 is equally distributed in both cell compartments while LNC19 is predominantly expressed in the nucleus. Furthermore, the silencing of LNC16 induced significant apoptotic resistance in the hox‐exposed hPASMCs, whereas the knockdown of LNC19 induced a significant anti‐proliferative phenotype. Transcriptomic analysis revealed that silencing LNC16 and LNC19 dysregulated pathways associated with ribosomal biogenesis and translation in the hox‐exposed hPASMCs. Considering the role of mTOR in translation and given the importance of cap‐independent translation, particularly the internal ribosome entry site (IRES) system, in the translation of hypoxia‐responsive genes to sustain the hypoxic response, the role of LNC16 and LNC19 in IRES‐dependent translation under hypoxia will be further investigated.

## 52. Revealing The Role of Interleukin 9 in the Pathogenesis of Pulmonary Hypertension

Medrano‐Garcia S^1,2,3^, Maroli G^1,2,3^, Zhang Y^4^, Seeger W^1,2,3^, Huber M^4^, Savai R^1,2,3^, Pullamsetti S^1,2,3^

^1^Institute For Lung Health (ILH), Justus Liebig University, Giessen, Germany, ^2^Max Planck Institute for Heart and Lung Research, Member of the German Center for Lung Research (DZL), Member of the Cardio‐Pulmonary Institute (CPI), Germany, ^3^Department of Internal Medicine, Justus‐Liebig University Giessen, Member of the DZL, Member of CPI, Germany, ^4^Institute for Medical Microbiology and Hygiene, Philipps‐University Marburg, Germany

T cell populations are divided into several subpopulations based on their cytokine profile, of which a newer subtype of Th cells has been linked to tumorigenesis: Th9 cells, which predominantly produce the cytokine IL‐9. Pulmonary hypertension (PH) is also characterized by a perivascular accumulation of T cells that likely contributes to the vascular changes. In PH, CD4 + T cells (T‐helpers, Th) accumulate and communicate with pulmonary artery cells and the surrounding environment via soluble (cytokines) and cell‐derived molecules. However, it is not known exactly which subpopulations of T cells are involved in the pathogenic mechanism. In addition, the role of Th9 cells and the cytokines they release (e.g. IL‐9) in the pathology of PH has not yet been investigated. The impact of Th9 on PH was investigated in vitro to study the functional changes in pulmonary arterial adventitial fibroblasts (PAAFs), pulmonary arterial smooth muscle cells (PASMCs) and pulmonary microvascular endothelial cells (PMVECs), and Th9 was also mapped and validated in lung tissues and cells from patients with idiopathic pulmonary PH (IPAH). Our data show that Th9 and IL‐9+ cells are increased in the perivascular compartment in lung tissue from IPAH patients, as well as IL‐9+ cells are increased in the perivascular compartment after 35‐day MCT rat model and 5‐week hypoxic exposure of mouse lungs. In addition, IL‐9 induces proliferation and migration of human donor and IPAH PASMCs and mouse control PASMCs, proliferation of IPAH PAAFs, and angiogenesis and proliferation of human and mouse PMVECs. In summary, our study demonstrates for the first time a role of IL9+ cells in PH pathology and a proliferative, migratory and angiogenic effect on pulmonary vascular cells, although the underlying mechanisms require further investigation.

## 53. Single‐Cell Atlas of The Human Lung in Pulmonary Arterial Hypertension

Brownstein A^1^, Wong B^1^, Graves T^2^, Aldred M^2^, Dai Z^3^, Yang X^1^, Eghbali M^1^, Hong J^1^

^1^University of California, Los Angeles, Los Angeles, USA, ^2^Indiana University, Indianapolis, USA, ^3^Washington University, St. Louis, USA

**Introduction:** Pulmonary arterial hypertension (PAH) is a complex disease characterized by progressive pulmonary vascular remodeling. Single‐cell profiling of lung tissue may provide novel insights into this incurable disease.

**Methods:** Single‐nucleus RNA sequencing was performed on human lung samples from the Pulmonary Hypertension Breakthrough Initiative using the 10x Genomics Chromium Fixed RNA Profiling workflow. Rigorous quality control measures were implemented, including the detection and removal of ambient RNA using CellBender and doublets using scDblFinder. Data normalization and dimensionality reduction were performed using Seurat, and alignment by sample and sequencing batch was conducted using Harmony. Cells were annotated by mapping onto the Human Lung Cell Atlas using Azimuth. Differential gene expression analysis was conducted using a robust pseudobulk approach with edgeR. Gene set enrichment analysis was performed using the Hallmark pathways. Cell type abundance was analyzed and correlated with markers of disease severity.

**Results:** Lung samples from 67 individuals were profiled, including 42 PAH and 25 age‐ and sex‐matched control lungs. After quality control, over 800,000 cells were captured, with a median of 12,000 cells per sample. Clustering uncovered over 30 cell types, representing endothelial, stromal, immune, and epithelial lineages of the lung. A median of 2,500 transcripts and 1,500 genes were detected per cell. Differentially expressed genes between PAH and control were detected in all major cell populations (FDR < 0.05). Comparative analysis across all cell types revealed interferon response in monocyte subpopulations and endothelial‐to‐mesenchymal transition in endothelial subpopulations as top upregulated pathways. Fibroblast and T lymphocyte subpopulations were more abundant in PAH lungs, with regulatory T cells correlating with higher pulmonary vascular resistance.

**Conclusion:** Comprehensive single‐nucleus profiling of human lungs in PAH revealed extensive gene and pathway dysregulation, underscoring the complexity of the disease's cellular pathobiology. This rich dataset offers opportunities to advance and refine our molecular understanding of PAH.

## 54. Echocardiographic Assessment for Responder Analysis in Pulmonary Arterial Hypertension Patients Treated With Parenteral Prostanoids

Papa S^1^, Scoccia G^1^, Adamo F^1^, Serino G^1^, Filomena D^1^, Maggio E^1^, Manzi G^1^, Mihai A^1^, Recchioni T^1^, Vizza C^1^, Badagliacca R^1^

^1^Sapianza University of Rome, Rome, Italy

**Background:** Although pulmonary arterial hypertension (PAH) prognosis has improved in the last two decades, patient outcomes are still unfavorable and there are unresolved issues regarding current therapeutic strategies.

**Objectives:** The present study aims to identify echocardiographic changes at 1‐year assessment from prostanoid initiation, predictive of long‐term survival.

**Methods:** One hundred and sixty‐three consecutive PAH patients (idiopathic PAH, 69%) referred to our center and started on parenteral prostanoids were followed with clinical, hemodynamic, and echocardiographic assessment.

**Results:** Improvement in RVEDA and RVFAC at 1‐year assessment was independently predictive of survival (c‐statistic: 0.83, C.I. 0.75‐0.92). ROC‐derived cut‐points of ΔRVEDA > ‐2.5 cm2 and ΔRVFAC > 5.6% were used for patients’ clinically meaningful responsiveness definition. After a mean follow‐up of 33 (IQR 10‐69) months, 68 patients (42%) died. Fifty‐five (34%) patients were full‐responders with significantly better survival rates compared with partially‐responders patients (108, 66%), respectively, 100%, 100%, 100%, and 100% versus 69%, 44%, 33%, and 13% after 1, 3, 5 and 10 years of follow‐up (p < 0.0001). At multivariate logistic analysis, PVR reduction was the only independent determinant of both Δ RVEDA and Δ RVFAC improvement (O.R. 0.76, 95% C.I. 0.66‐0.89, p < 0.001). Although there was no significant difference in maximal dosage reached over time between survivors and non‐survivors (55.8 ± 33.2 vs 48.1 ± 25.9 ng/kg/min, respectively; p=ns), full‐responders were characterized by a progressive improvement of RVEDA and RVFAC with increasing prostanoid dosage compared with partially‐responders (Full‐responders: Δ RVEDA vs prostanoid dosage: r2 = 0.61, p = 0.0001, y = ‐11.3‐0.27x; Δ RVFAC vs prostanoid dosage: r2 = 0.12, p = 0.01, y = 9.43 + 0.09x). Patients starting parenteral prostanoids at ESC/ERS high‐risk score experienced a reduction of 52% (O.R. 0.48, 95% C.I. 0.26‐0.79, p < 0.01) in the odds of achieving a clinically meaningful responsiveness definition.

**Conclusions:** Echocardiography assessment at 1 year from prostanoid initiation allows for a clinically meaningful responsiveness definition, able to identify patients characterized by long‐term survival.

## 55. Targeting Aurora Kinase B‐Induced Senescence: A Novel Therapeutic Avenue in Pulmonary Arterial Hypertension

Lemay S^1^, Mougin M^1^, Sauvaget M^1^, El Kabbout R^1^, Valasarajan C^2^, Yamamoto K^1^, Martineau S^1^, Pelletier A^1^, Bourgeois A^1^, Romanet C^1^, Breuils‐Bonnet S^1^, Montesinos M^3^, Lu M^3^, Chen H^3^, Théberge C^1^, Potus F^1^, Pullamsetti S^2^, Provencher S^1^, Bonnet S^1^, Boucherat O^1^

^1^Pulmonary Hypertension Research Group, Quebec Heart and Lung Institute, Quebec, Canada, ^2^Max Planck Institue for Health and Lung Research, German Center for Lung Reasearch, Giessen, Germany, ^3^Morphic Therapeutic, Waltham, United‐States

Pulmonary arterial hypertension (PAH) is characterized by pulmonary artery (PA) remodeling leading to right ventricular failure and premature death. Extensive proliferation of PA smooth muscle cells (PASMCs) is an important feature of PAH accounting for vascular remodeling.

To discover novel actionable targets involved in vascular remodeling, we performed a comparative RNA‐sequencing analysis between control and PAH‐PASMCs. To extract reliable targets, we enriched our experiment with two publicly available datasets conducted on comparable cell lines. After merging the three datasets, 136 genes were found to overlap, of which 126 and 10 were up‐ and down‐regulated, respectively. A connectivity map analysis using SigCom Library of Integrated Network‐based Cellular Signatures was performed on the commonly upregulated genes and identified aurora kinase B (AURKB) inhibitors as the top reverser drug of PAH‐PASMCs gene signature. In support of this, aurora kinase B (AURKB), known to play a critical role in the progression of mitosis, was present among the overlapping upregulated genes in PAH‐PASMCs. Further experiments revealed that FOXM1 positively regulates AURKB. Pharmacological (Barasertib) and molecular inhibition of AURKB reduced PAH‐PASMC proliferation (EDU incorporation, Ki67 & PLK1 expression), survival (Tunel, Annexin V & Survivin expression) and was associated with accumulation of mitotic defects. Acquisition of a senescence‐like phenotype was observed in PAH‐PASMCs that escaped apoptosis following AURKB inhibition (SA‐ βGal, p21, SASP…). Barasertib treatment significantly improved pulmonary vascular remodeling and hemodynamics in MCT and Su/Hx rats with established PAH. A therapeutic effect was also observed in human precision‐cut lung slices isolated from PAH patients. Finally, the combination of Barasertib with the p21 attenuator UC2288 was more effective in reducing vascular remodeling in Su/Hx and MCT rats than either drug alone.

Our research identified AURKB as a novel therapeutic target and suggests that combining anti‐remodeling drugs with senotherapeutics may be more effective to counteract vascular remodeling.

## 56. Preclinical Studies Revealed Integrin α5β1 as a Key Target for the Treatment of Pulmonary Arterial Hypertension

Lemay S^1^, Montesinos M^2^, Grobs Y^1^, Yokokawa T^3^, Shimauchi T^4^, Mougin M^1^, Romanet C^1^, Sauvaget M^1^, Gilbert M^1^, Breuils‐Bonnet S^1^, Bourgeois A^1^, Théberge C^1^, Pelletier A^1^, El Kabbout R^1^, Martineau S^1^, Yamamoto K^1^, Ray A^2^, Lippa B^2^, Goodwin B^2^, Lin F^2^, Wang H^2^, Dowling J^2^, Lu M^2^, Qiao Q^2^, McTeague T^2^, Moy T^2^, Potus F^1^, Provencher S^1^, Boucherat O^1^, Bonnet S

^1^Pulmonary Hypertension Research Group, Quebec Heart and Lung Institute, Quebec, Canada, ^2^Morphic Therapeutic, Waltham, United‐States, ^3^Departement of Cardiovascular Medicine, Fukushima Medical University, Japan, ^4^Departement of Anesthesiology, St. Mary's Hospital, Kurume, Japan

**Introduction:** Pulmonary arterial hypertension (PAH) is characterized by progressive obstruction and decreased compliance of pulmonary arteries (PA), leading to right ventricular failure and premature death. Sustained proliferation and resistance to apoptosis of PAs smooth muscle and endothelial cells (PASMCs, PAECs) and accumulation of extracellular matrix (ECM) elements contribute to vascular remodeling. Integrins, members of the cell adhesion receptor superfamily, are known to promote cell proliferation and survival through ECM binding. Despite the recognition of the role of ECM elements in vascular remodeling in PAH, the contribution of integrins remains poorly studied. We hypothesized that integrin signaling could promote PAH‐PASMCs and PAH‐PAECs proliferation and resistance to apoptosis contributing to vascular remodeling.

**Methods & Results:** Using Western blot (WB) and Meso Scale Discovery, we found that α5β1 was upregulated in distal PAs, PA smooth muscle cells (PASMC) and PA endothelial cells (PAEC) from PAH patients compared to controls. Small molecule, antibody (M200), or siRNA‐mediated inhibition of α5β1 decreased PAH‐PASMC and PAH‐PAEC proliferation (WB, MCM2 and Ki67 labeling), resistance to apoptosis (WB, Survivin and Annexin V labeling) and migration (scratch test assay). RNA sequencing analysis revealed that inhibition of α5β1 regulates a FOXM1‐dependant gene network by activating focal adhesion kinase, resulting in mitotic defects (α‐tubulin and pericentrin labeling). In vivo, in both monocrotaline and Sugen/hypoxia rats with established PAH, inhibition of α5β1 using a small molecule inhibitor or a neutralizing antibody alone or in combination with macitentan and tadalafil improved hemodynamics (RHC and echocardiography: mean pulmonary arterial pressure, cardiac output, PAAT) and vascular remodeling (Elastica Van Gieson staining). Ex vivo, inhibition of α5β1 decreased vascular remodeling in human precision‐cut lung slices (PCLS) from PAH patients and attenuated growth factors‐induced vascular remodeling in control PCLS.

**Conclusion:** Integrin α5β1 plays a key role in vascular remodeling in PAH and represents a promising therapeutic target.

## 57. Integrin α5β1 Represents a New Therapeutic Target to Counter Pathological Remodeling of the Right Ventricle in Pulmonary Arterial Hypertension

Lemay S^1^, Montesinos M^2^, Grobs Y^1^, Romanet C^1^, Sauvaget M^1^, Breuils‐Bonnet S^1^, Bourgeois A^1^, Théberge C^1^, Martineau S^1^, Lippa B^2^, Goodwin B^2^, Lin F^2^, Wang H^2^, Dowling J^2^, Lu M^2^, Qiao ^2^, McTeague T^2^, Moy T^2^, Potus F^1^, Provencher S^1^, Boucherat O^1^, Bonnet S^1^

^1^Pulmonary Hypertension Research Group, Quebec Heart and Lung Institute, Quebec, Canada, ^2^Morphic Therapeutic, Waltham, United‐States

**Introduction:** Right ventricular (RV) function is an important prognosis factor in pulmonary arterial hypertension (PAH). In response to pressure overload, the RV undergoes morphological changes driven by the accumulation of extracellular matrix (ECM) elements and cardiomyocyte hypertrophy. Integrins (ITG) are members of the cell adhesion receptor superfamily. Through ECM‐binding, ITG are known to transduce extracellular cues to cardiac cells resulting in the activation of pro‐hypertrophic and pro‐fibrotic signaling pathways. Among them, integrin α5β1 is one of the most abundant heterodimers in cardiac tissue. We thus hypothesized that α5β1 signaling promotes pathological RV remodeling.

**Methods & Results:** Using Western blot (WB), we found elevated levels of Itgα5 and Itgβ1 protein in RV isolated from PAH patients compared to controls. Similar findings were observed in monocrotaline (MCT) and pulmonary artery banding (PAB) rats. In adult rat cardiomyocytes, pharmacological inhibition of α5β1 decreased phenylephrine‐induced hypertrophy and reversed established hypertrophy induced by MCT treatment (assessment of cell surface area by F‐actin labeling). In human RV fibroblasts (RVFB) inhibition of α5β1 prevented TGFβ1‐induced activation of control RVFB and decreased proliferation and activation of RVFB derived from PAH patients (WB PCNA, αSMA, COL1; immunofluorescence Ki67). In vivo, inhibition of α5β1 using a small molecule inhibitor or a blocking‐antibody improved RV dysfunction (CO, TAPSE, RVFAC, RV strain, as assessed by echocardiography, right heart catheterization and cardiac magnetic resonance imaging) and diminished RV hypertrophy and fibrosis (Fulton index, H&E and Masson's trichrome staining) in MCT‐injected rats with established PAH and PAB rats. Ex vivo, inhibition of α5β1 attenuated cardiomyocyte hypertrophy and RV fibrosis in human precision‐cut RV slices exposed to phenylephrine and TGFβ1.

**Conclusion:** Our results suggest that integrin α5β1 plays a key role in pathological RV remodeling in PAH.

## 58. Pulmonary Hypertension is Highly Prevalent in Rheumatic Heart Disease in Sub‐Saharan Africa: A Systematic Review and Meta‐Analysis

Zeder K^1,2^, Wang L^3^, Santi A^1^, Ngah V^1^, Robbins E^4^, Diao G^3^, Maron B^1,5^

^1^University of Maryland ‐ Institute for Health Computing, Bethesda, USA, ^2^Division of Pneumology, Medical University of Graz, Graz, Austria, ^3^Department of Biostatistics and Bioinformatics, George Washington University, District of Columbia, USA, ^4^Division of Pulmonary and Critical Care Medicine, University of Maryland School of Medicine, Baltimore, USA, ^5^Department of Medicine, University of Maryland School of Medicine, Baltimore, United States

**Background:** Rheumatic heart disease (RHD) plays a major role in global disease burden with most patients living in low‐middle income countries, particularly across sub‐Saharan Africa (SSA). In RHD, post‐streptococcal rheumatic fever causes valvular heart disease leading to pulmonary hypertension (PH), an independent predictor of increased morbidity and early mortality. Despite the importance of PH‐RHD clinically, its prevalence in SSA is unknown.

**Aim:** To determine the prevalence of RHD‐PH in SSA.

**Methods:** We performed a systematic review following PRISMA and GATHER guidelines searching PubMed, Embase, Web of Science, and LILACS for original studies for the prevalence of RHD‐PH in 15 pre‐selected SSA countries based on population size and income level. We included studies published between 1973 (first definition of PH) and September 31, 2023; studies without a clear PH definition were excluded. A meta‐analysis was performed using a random‐effects model.

**Results:** In total, we included N = 18 studies published between 1991‐2023, comprising 3,181 patients (range of mean age: 6‐53 yr). Of all studies, 72%, 11%, and 17% originated from high‐middle, lower‐middle and low‐income countries, respectively, and 72% were from Southern Africa. In 89% (N = 16) of studies, echocardiography was used to diagnose PH. The estimated mean PH prevalence based on estimated systolic pulmonary arterial pressure > 35 mmHg was 64.8% (95%CI: 47.9‐80.0% from N = 12 reports) and was 3‐fold greater in severe compared to incident RHD (78.2% [95%CI: 69.6‐85.4%] from N = 10 reports vs. 25.7% [95%CI: 13.8‐39.8%] from N = 6 reports; p < 0.001). Amongst the studies, 72% (N = 13), 6% (N = 1), and 22% (N = 4) had low, intermediate, and high risk of bias, respectively.

**Conclusion:** PH is extremely common in RHD in SSA. These findings warrant prospective studies that quantitate the contribution of RHD‐PH to global disease burden as well as public health measures aimed at PH risk reduction in RHD through primary prevention of RHD and mitigation of RHD complications.

## 59. Erythrocytosis‐Mediated Hyperviscosity Syndrome Exacerbated Bleeding in Sugen‐ Hypoxia Rat Model

Ishimwe J^1^, Thirumurthy G^1^, Pinkus C^1^, Jain P^1^, Nurse T^1^, Babbs K^1^, Lachey J^1^, Lerner L^1^, Seehra J^1^, Joshi S^1^

^1^Keros Therapeutics, Lexington, USA

The Sugen‐hypoxia rat model is widely used to study pulmonary arterial hypertension (PAH). Sotatercept, a drug recently approved for the treatment of PAH, is associated with severe thrombocytopenia and erythrocytosis, which, if severe, may increase the risk of hyperviscosity syndrome (HVS)1. HVS is a condition of elevated blood viscosity due to pathological increases in certain blood components, including red blood cells (RBCs). HVS can limit tissue perfusion and increase the risk of bleeding, especially when the endothelial barrier is impaired. It is unclear whether erythrocytosis combined with severe thrombocytopenia may directly contribute to HVS‐related bleeding events. We evaluated whether erythrocytosis‐mediated HVS combined with thrombocytopenia exacerbates bleeding in the Sugen‐hypoxia rat model. Male rats received a single dose of Sugen5416 (Su, 20 mg/Kg sc) and epoetin‐alpha (EPO, 7200U/Kg thrice weekly sc) or vehicle under normoxic (SuNx‐EPO) or hypoxic (SuHx‐EPO, 10% O₂) conditions for 5 weeks and compared to normoxia (Nx) controls. Complete blood count, blood viscosity, bleeding events, and survival were evaluated. EPO treatment increased RBCs (SuNx‐EPO: +41%; SuHx‐EPO: + 24%) and hemoglobin (SuNx‐EPO: + 35%; SuHx‐EPO: + 37%), and decreased platelets (SuNx‐EPO:‐77%; SuHx‐EPO:‐194%), compared to non‐EPO‐treated Nx controls. Increased erythrocytosis (r = 0.6604, p < 0.0001) and hemoglobin (r = 0.6813, p < 0.0001) positively correlated with increased blood viscosity. Gastrointestinal bleeding and nosebleed were observed in SuHx‐EPO rats, whereas SuNx‐EPO rats had increased RBCs without bleeding events. Only SuHx‐EPO rats displayed a reduced survival rate (‐62.5%, p < 0.0001). Our data demonstrate that hypoxia increased susceptibility to HVS‐related bleeding and mortality in response to EPO‐induced erythrocytosis and severe thrombocytopenia in Sugen rats. These findings suggest that increased blood viscosity, partly due to erythrocytosis, along with accompanying severe thrombocytopenia, may potentially raise the risk of bleeding in patients with chronic diseases associated with systemic hypoxia, such as PAH.

1WINREVAIR USPI. (sotatercept‐csrk) for injection, for subcutaneous use. US Prescribing Information. Merck & Co., Inc.

## 60. Pulmonary Arterial Pruning is Associated With Death in Copdgene Participants Who Smoke With Normal Spirometry

Johnson S^1^, Ross J^1^, Come C^2^, Diaz A^1^, Pistenmaa C^1^, Nardelli P^1^, Washko G^1^, Rahaghi F^1^, Estepar R^1^

^1^Brigham And Women's Hospital, Boston, USA, ^2^Lahey Hospital and Medical Center, Burlington, USA

**Background:** In COPD, the risk of adverse outcomes associated with chest computed tomography (CT)‐based metrics of pulmonary vascular disease including loss of small pulmonary arterial vascular volume or pruning, increased right ventricular volume and pulmonary artery dilation, are well described. The relevance of these imaging features in smokers without COPD is not known. We investigated the association of CT features along a pulmonary vascular continuum with death in smokers without COPD.

**Methods:** We analyzed subjects from the Genetic Epidemiology of COPD (COPDGene) study Phase 1 visit with available vascular volumes, PA to aorta (PA/Ao) diameter and right/left ventricle (RV/LV) volume measurements with FEV1/FVC □ 0.7 and FEV1 □ 80% (n = 3,140). Cox proportional hazard models were used to compare the standardized predictive ability of each vascular feature with all‐cause mortality.

**Results:** The prevalence of current and former smokers was similar (53.8% and 46.2%) with median age 56 [29] years and 34.0 [23.6] pack‐year exposure; subjects had minimal emphysema (1.0[2.4]%). At 15‐year follow up, overall survival in the quartile with the least pruning to that with the most pruning, was 93.8% vs. 85.1% (p < 0.0001). In a multivariable model including adjustment for age, BMI and pack‐years, each standard deviation decrease in small vessel volume (< 5mm2) (more pruning) was associated with a 29% increased risk of death (HR 1.29, 95% CI 1.15, 1.46; p < 0.0001). In an additional model inclusive of the stronger extra‐parenchymal vascular predictor of death (PA/Ao over RV/LV), pruning remained significant.

**Conclusions:** Among smokers without COPD on spirometry and with minimal emphysema on imaging, loss of distal pulmonary vascular volume is associated with increased risk of death. As pulmonary vascular injury may precede development of COPD and is associated with pulmonary hypertension (PH), these results question whether small vascular volume may be a measurable therapeutic target for disease prevention and progression.

## 61. Methamphetamine‐Associated Pulmonary Arterial Hypertension is Not Associated With Worse Right Ventricular Function Than Idiopathic Pulmonary Arterial Hypertension When Compared by Cardiac MRI

Baird A^1^, Hutman‐Zahler A^1^, MacLean E^1^, Ingram D^1^, Dranow E^1^, Ibraham M^1^, Ryan J^1^, Clapham K^1^

^1^University Of Utah, Salt Lake City, United States

**Background:** Methamphetamine is an illicit substance of growing public health concern, and a cause of both pulmonary arterial hypertension as well as non‐ischemic cardiomyopathy. Patients with methamphetamine associated pulmonary arterial hypertension (Meth‐APAH), compared to those with idiopathic pulmonary arterial hypertension (IPAH) are known to have worse functional class, as well as lower cardiac outputs after accounting for mean pulmonary artery pressures and pulmonary vascular resistance. We postulated that individuals with Meth‐APAH would have more right ventricular dysfunction assessed by cardiac MRI than those with IPAH.

**Methods:** This retrospective study included 13 individuals with Meth‐APAH and 13 with IPAH, matched by PVR measured by right heart catheterization at the time of diagnosis. MRI indices of right ventricular size and function were compared.

**Results:** The majority of individuals were female in both groups (IPAH: 11/13, Meth‐APAH: 10/13); those with IPAH were older (50.9 standard deviation (SD) +/‐ 12.3 vs. 39.1 SD+/‐ 11.8). The mean PVR in the IPAH group was 9.3 Woods Units SD+/‐ 7.1 vs. 9.5 Woods Units SD+/‐ 5.6 in the Meth‐APAH group, p = 0.95. MRI measures of right ventricular size, systolic function, and strain were not significantly different between IPAH and Meth‐APAH groups (right ventricular end diastolic index 103.2 mL SD+/‐ 39.5 vs. 120.5 SD+/‐ 32.8, p = 0.26, right ventricular ejection fraction 39.9% SD+/‐ 12.1 vs. 38.9% SD+/‐ 16.2, p = 0.86, right ventricular global longitudinal strain ‐18.4% SD+/‐ 5.1 vs. ‐16.7% SD+/‐ 4.8, p = 0.39).

**Conclusions:** In this cohort, we did not detect a statistically significant difference in right ventricular function as measured by cardiac MRI between Meth‐APAH and IPAH patients in contrast to previously reported invasive hemodynamic observations. Further work with larger sample sizes may shine light on the growing role for cardiac MRI in disease prognostication and treatment response in this population.

## 62. Building A Pulmonary Hypertension Automated Follow up Order Process

Markley I^1^, Varghese N^1,2^, Ely E^1^, Fombin R^1^, Whalen E^1^

^1^Texas Children's Hospital, Houston, United States, ^2^Baylor College of Medicine, Houston, United States

**Background:** The electronic medical records (EMR) designed to support patient care can be fraught with increased burden to track disease progression across lab monitoring, diagnostic imaging studies, and functional testing across its many data points. We worked with our EMR team to build a pulmonary hypertension (PH) database to collect and report discrete data (labs, encounters, note information, results) for the center's PH population, streamlining workflow process to reduce order redundancy and errors.

**Methods:** A discrete picklist (SmartList) was created for capturing required follow up orders for future visits for PH patients. The list consisted of standard monitoring labs, functional, and diagnostic tests. The SmartList was accessed from within the PH office note during the clinic encounter. Once the SmartList was completed, a dynamic list of orders (SmartSet) was generated for the patient based on the clinician's selections, with prefilled information set by the PH team.

**Results:** Creation of SmartList and subsequent SmartList improved clinic flow, resource usage, and allowed for future care planning. The SmartList enabled clinicians to place point of care orders with complete detail to minimize delays in scheduling and actualization of orders. There was a decrease in order errors and corrections as well as time spent to address these. There remains a need to flag follow up orders not completed by patient during the previous clinic visit when reviewing for upcoming clinic appointments on the PH dashboard.

**Conclusions:** Collaboration between information services and the clinical team led to creation of a clinically actionable dashboard embedded within the EMR and further customization to include a SmartList tool. Use of this tool allowed effortless and efficient order entry, while improving clinic flow, resource usage, and future care planning. We plan to continue building on this functionality to improve clinic efficiency and patient outcomes with emphasis on quality improvement.

## 63. A Monoclonal Antibody Targeting The ZIP12 Transporter Attenuates Severe Experimental Pulmonary Arterial Hypertension

Hopley S^1^, Chen C^2^, Xie C^2^, Baxan N^2^, Niglas M^2^, Butt R^1^, Hamblin P^1^, Wilkins M^2^, Zhao L^2^

^1^Apollo Therapeutics, Cambridge, United Kingdom, ^2^Imperial College London, London, United Kingdom

Pulmonary hypertension (PH) remains an unmet clinical need, despite new advances. In particular, direct targeting of the remodelled pulmonary vasculature is still a gap in our therapeutic armoury.

ZIP12 (SLC39A12) is a non‐voltage gated Zn2+ ion transporter belonging to the solute carrier gene superfamily. It has very low expression in healthy lung, but is upregulated in the lungs of PH patients. Knockout of ZIP12 abrogates PH in preclinical models. We have generated a ZIP12 function‐blocking mAb and evaluated in preclinical PH models, using ZIP12 humanised rats (huslc39a12), generated to express the human protein.

ZIP12 immunogens were generated and immunized into mice, followed by mAb generation using hybridoma technology. The preferred mAb, APL‐13, was tested for preclinical efficacy in experimental models of PH. Briefly, huslc39a12 rats were administered APL‐13 or an isotype‐control mAb (0.1‐10 mg/kg, i.p. twice weekly) in chronic, Sugen/hypoxia and monocrotaline models. Physiological measurements (PAP, RVH and cardiac MRI) and lung histological endpoints were assessed.

Histological examination demonstrated that APL‐13 attenuated pulmonary vascular remodelling in all models of pulmonary hypertension tested. These results were accompanied by dose dependent and significant decreases, relative to isotype matched control, in PAP, RSVP and RVH. We observed improved cardiac output as measured by increased right ventricular ejection fraction and ventricular‐arterial coupling (RVEF and Ees/Ea). There was a statistically significant reduction in the numbers of both occluded and muscularized vessels following treatment with APL‐13. No significant changes were observed on systolic blood pressure (SBP) or haematocrit levels following treatment.

In conclusion, APL‐13 is a first‐in‐class mAb which has been shown to abrogate and reverse severe experimental PH in rat models with no significant impact on the systemic vasculature. APL‐13 is now being progressed into clinical studies for the treatment of PH.

## 64. Cyclophilin D Deficiency Triggers Spontaneous Development of Pulmonary Hypertension and Emphysema During Aging

Castro C^1^, Hadzic S^1^, Kojonazarov B^1,2^, Kraut S^1^, Hecker M^1^, Seeger W^1,2^, Schermuly R^1^, Ghofrani H^1^, Weissmann N^1^, Sommer N^1^, Pak O^1^

^1^Excellence Cluster Cardio‐Pulmonary Institute, University of Giessen and Marburg Lung Center (UGMLC), member of the German Center for Lung Research (DZL). Justus‐Liebig‐University, Giessen, Germany, ^2^Institute for Lung Health (ILH), Giessen, Germany

**Background:** Pulmonary hypertension (PH) associated with chronic obstructive pulmonary disease (COPD) leads to increased morbidity and mortality. Mitochondrial cyclophilin D (CypD), encoded by the peptidylprolyl isomerase F (Ppif) gene, regulates the mitochondrial permeability transition pore and may contribute to PH‐COPD by modulating apoptosis. Previously, we found that CypD modulates pulmonary vasoconstriction. Therefore, in this study, we investigated the role of CypD in PH‐COPD development.

**Methods and Results:** Wild‐type (WT) and CypD knockout (Ppif‐/‐) mice (aged 3 months) were exposed to cigarette smoke (CS) or room air (RA) for 8 months (6 hours/day, 5 days/week). Interestingly, RA‐exposed Ppif‐/‐ mice (now aged 11 months) exhibited signs of PH compared to RA‐exposed WT mice (also aged 10‐11 months), characterized by increased right ventricular systolic pressure (RVSP) and right ventricular hypertrophy. At 3 months, RVSP was similar between WT and Ppif‐/‐ groups. Additionally, 8‐month RA‐exposed Ppif‐/‐mice showed signs of emphysema compared to RA‐exposed WT mice, as indicated by in vivo lung function tests, µCT scans, and post‐mortem stereological analysis. However, after 8 months of CS exposure, Ppif‐/‐ mice developed PH and emphysema to a similar extent as WT mice. CypD deficiency increased the expression of the endoplasmic reticulum (ER) stress marker X‐box binding protein 1 (XBP1) and the cell cycle arrest marker p21 in the lung homogenates of 3‐month‐old mice, while it reduced cyclin D1 expression in 12‐month‐old mice compared to WT mice of the same age. Furthermore, CypD protein levels were reduced in lung homogenates of COPD patients.

**Conclusion:** CypD deficiency promotes pulmonary alterations resembling PH and emphysema during aging but does not exacerbate these conditions following CS exposure. Inhibition of cellular proliferation leading to parenchymal changes may underlie these alterations.

## 65. Early Immuno‐Ageing (EIA): An Unexplored Risk Factor in Pulmonary Hypertension?

Valasarajan C^1,2^, H. Meyer D^3^, Maroli G^1^, Weigert A^4^, Rosenkranz S^5^, H. Ghofrani A^1^, Seeger W^1,2^, Tello K^1^, Schumacher B^3^, Savai R^1,2^, Pullamsetti S^1,2^

^1^Department of Internal Medicine, Member of the DZL, Member of CPI, Justus Liebig University, Giessen, Germany; Institute for Lung Health (ILH), Member of the DZL, Justus Liebig University, Giessen, Germany, ^2^Max Planck Institute for Heart and Lung Research, Member of the German Center for Lung Research (DZL), Member of the Cardio‐Pulmonary Institute (CPI), Bad Nauheim, Germany, ^3^Institute for Genome Stability in Ageing and Disease, Medical Faculty, Cologne Excellence Cluster for Cellular Stress Responses in Ageing‐Associated Diseases (CECAD), Center for Molecular Medicine Cologne (CMMC), University of Cologne, Cologne, Germany, ^4^Institute of Biochemistry I, Goethe‐University Frankfurt, Frankfurt, Germany, ^5^Department of Cardiology, Heart Center at the University Hospital Cologne, and Cologne Cardiovascular Research Center, Cologne, Germany

Ageing is currently defined at two levels: Chronological age, calculated based on the years from the time of birth and Biological age, defined by the functional ability and molecular make up of cells and organs. An accelerated biological clock in response to stress factors can have detrimental effect and may contribute to the initiation or progression of various pathophysiological conditions. Early immuno‐ageing, accelerated ageing of the immune cells, have shown to play a detrimental role in various age‐associated diseases and vascular homeostasis. Pulmonary hypertension (PH), an age associated vascular disease characterized by elevated pressures in the lung vasculature because of pathological remodelling of blood vessels. In this study, we investigated the existence of early immuno‐ageing in the PH patients. The initial interest to look for early immuno‐ageing in PH patients was triggered from the RNA‐seq data and multispectral flow cytometry data of the PBMCs obtained from PH patients. From the pathways analysis of the transcriptomic dataset, the ageing associated pathways were significantly upregulated in the PH patient samples. Further, looking into the immune population the PH patient samples showed a skew towards the elevated myeloid population, which from previous studies is known to be a characteristic of immuno‐ageing. The ageing clocks that are currently in use to calculate the biological age are mainly based on the DNA methylation data, which has been a major bottleneck in this field. To overcome this, we used a unique transcriptomic based clock to calculate the early immuno‐ageing. The data derived from the ageing clock have shown immune cells of the PH patients has a mean accelerated age of five years. Further, this data correlated with the reduced telomere length and dysregulated autophagy in the immune cells of the PH patients. These data confirms the existence of the early immuno‐ageing the PH patients.

## 66. Tbx4 – A Potential Transcription Factor in Postnatal Pulmonary Vascular Development and Pediatric Pulmonary Arterial Hypertension

V. Cherian A^1,2^, Ebeling M^1,2^, De P^1,2^, Medrano‐Garcia S^1,2^, Mordjana N^1,2^, Valasarajan C^1,2^, Alcazar M^3^, Seeger W^1,2^, Savai R^1,2^, Pullamsetti S^1,2^

^1^Max Planck Institute for Heart and Lung Research, Bad Nauheim, Germany, ^2^Center for Department of Internal Medicine, Member of the DZL, Member of CPI, Justus Liebig University, Giessen, Germany; Institute for Lung Health (ILH), Member of the DZL, Justus Liebig University, Giessen, Germany, ^3^University of Cologne, Faculty of Medicine and University Hospital Cologne, Cologne, Germany

Pediatric pulmonary arterial hypertension (PPAH) is associated with a variety of cardiac, pulmonary and systemic diseases in neonates and infants. Mutations in Bone Morphogenetic Protein Receptor 2 (BMPR2) can cause PAH in adults. However, the genetic architecture of pediatric PAH differs from adult PAH and appears to be enriched with TBX4 and ACVRL1 mutations. However, the nature and mechanisms underlying the pathobiology of PPAH in the background of the mutations remain unexplored. To study PPAH in detail, we bred mice with a Tbx4 mutation, which is commonly observed in PPAH patients and which exhibit cardiac hypertrophy and deformation similar to PAH patients; the development of PAH is confirmed by echocardiography. However, in humans and mice, we did not observe vascular remodeling in pulmonary vessels. 3D analysis using a confocal microscope revealed that the number of vessels decreased in Tbx4 mutant mice compared with wild‐type mice. 3D analysis of pulmonary vessels at different developmental stages of mice shows that microvessels are mainly formed after birth and increase dramatically in early lung development. We then investigated the expression of Tbx4 in different cell types by single cell sequencing. Tbx4 is highly expressed in mesenchymal cells, of which pericytes are responsible for the formation and stability of blood vessels. Using FACS, high‐resolution 3D microscopy and single‐cell sequencing, we demonstrated that the number of pericytes is significantly reduced in mutant Tbx4 mice. We then knocked out Tbx4 in pericytes at the early stage of lung development using PdgfrB CreErt2;Tbx4cond mice and observed a reduced number of pulmonary vessels. Reduced numbers of pericytes in Tbx4 mutant mice could inhibit the formation of new blood vessels during lung development, and a reduction in the number of blood vessels could contribute to the occurrence of PPAH.

## 67. Effect of BMP9 in Regulating Bmp/TGF‐β Pathways in TGFB1‐Silenced Human Pulmonary Microvascular Endothelial Cells

Keskinidou C^1^, Lotsios N^1^, Dimopoulou I^1^, Kotanidou A^1^, Langleben D^2^, Orfanos S^1^, Vassiliou A^1^

^1^First Department of Critical Care Medicine & Pulmonary Services, School of Medicine, National and Kapodistrian University of Athens, Evangelismos Hospital, 106 76 Athens, Greece, Athens, Greece, ^2^Center for Pulmonary Vascular Disease, Azrieli Heart Center and Lady Davis Institute, Jewish General Hospital, McGill University, Montreal, QC H3T 1E2, Canada, Montreal, Canada

Pulmonary arterial hypertension (PAH) is a progressive disorder; abnormalities in bone morphogenetic protein (BMP) signaling pathways contribute to PAH onset. Mutations of the BMP receptor type II (BMPR2) gene are commonly reported in hereditary PAH, while dysregulated signaling of the transforming growth factor‐β (TGF‐β) superfamily contributes to disease development and severity. TGF‐β1 levels are elevated in serum and lung samples of PAH patients. The principal ligand of BMRP2, BMP9, may represent a possible PAH therapy; its effects on other BMP‐related signaling molecules when administered exogenously may depend on the expression levels of BMPR2. We have demonstrated that, in human pulmonary microvascular endothelial cells (HPMECs), silencing of either the BMPR2 or the aquaporin 1 (AQP1) gene results in decreased expression of TGFΒ1. AQP1 has gained attention in the setting of PAH for its role in regulating endothelial cell permeability, migration, and proliferation. Having shown that BMP9 could restore TGFB1 mRNA and protein levels in the HPMEC‐ST1.6 R cell line silenced for BMPR2, we proceeded to further explore the expression of BMP/TGF‐β‐related signaling molecules in TGFB1‐silenced HPMECs, compared to untreated cells. Silencing of TGFB1 resulted in the reduced expression of BMPR2 (p < 0.05) and BMP10 (p < 0.05) and increased the expression of BMP9 (p < 0.05). No effect was observed regarding AQP1 expression. The effect of exogenous BMP9 was then examined; administration of BMP9 did not restore TGFB1 (p < 0.01) and BMP10 levels (p < 0.05) to control levels, while it resulted in decreased AQP1 expression (p < 0.01). BMPR2 expression tended to remain low (p = 0.05). Our findings suggest that TGFB1 silencing and BMP9 administration in HPMECs alters the expression of key molecules in the BMP/TGF‐β signaling pathway, and moreover that disruptions in this complex pathway may directly impact endothelial function, though this finding might be cell‐dependent.

## 68. Loss of Notch Transcription Factor RBPJ Partially Protects Mice From Chronic‐Hypoxia‐Induced Pulmonary Hypertension

Kleinhenn V^1^, Hadžić S^1^, Sydykov A^1^, Čilić A^1^, Wu C^1^, Giaimo B^2^, Pak O^1^, Sommer N^1^, Weissmann N^1^, Borggrefe T^2^, Veith C^1^

^1^Universities of Giessen and Marburg Lung Center (UGMLC), Member of the German Center for Lung Research (DZL), Excellence Cluster Cardio‐Pulmonary Institute (CPI), Justus‐Liebig University, 35392 Giessen, Germany, ^2^Institute of Biochemistry, Justus‐Liebig‐University, 35392 Gießen, Germany

Pulmonary hypertension (PH) is a severe, life‐threatening disease characterised by increased pulmonary vascular resistance and pulmonary arterial pressure due to vascular remodelling. Vascular remodelling causes right heart hypertrophy, which can lead to right ventricular failure and death, if left untreated. Chronic alveolar hypoxia significantly contributes to pulmonary vascular remodelling, triggering the uncontrolled proliferation of pulmonary arterial smooth muscle cells (PASMC) and dysfunction of pulmonary arterial endothelial cells (PAEC), leading to the obliteration of small pulmonary arteries.

The evolutionary highly conserved Notch signalling pathway is known to play a crucial role in vascular development and homeostasis by regulating various aspects of endothelial and smooth muscle cell behaviour. While the involvement of Notch receptors in PH pathogenesis is already characterised, the role of the Notch downstream signalling remains unclear. In this context, we aim to investigate the role of the Notch transcription factor Recombination Signal Sequence‐Binding Protein Jκ (RBPJ) in vascular cell dysfunction underlying PH pathogenesis.

Our in vivo data in tamoxifen‐inducible Rbpj‐knockout mice using STOCK Rbpjtm1Hon Tg(Acta2‐cre/ERT2)12Pcn (smooth muscle cell‐specific) and STOCK Rbpjtm1Hon Tg(Tek‐cre/ERT2)1Soff (endothelial cell‐specific) mice, indicate a partial protection from chronic hypoxia‐induced PH, by negatively affecting the hypoxia‐induced increase in right ventricular systolic pressure and right heart hypertrophy/function. Notably, the Acta2‐, but not the Tek‐specific loss of Rbpj protects mice from pulmonary vascular remodelling.

In in vitro studies using primary human PASMC and PAEC, RBPJ is identified as a regulatory factor affecting proliferation, possibly via the PCNA pathway. In addition, our findings suggest a crosstalk between RBPJ and hypoxia‐inducible factor (HIF) signalling. Following Rbpj knockdown in hypoxic PASMC and PAEC, HIF stabilisation is negatively affected.

In conclusion, our data provide the first evidence for a role of RBPJ in chronic hypoxia‐induced PH in mice by influencing vascular cell dysfunction, possibly via HIF signalling.

## 69. Excessive Ventilatory Response to Exercise and It´s Association With Chronic Thromboembolic Pulmonary Hypertension Phenotypes Through Cluster Analysis

Cordeiro A^1^

^1^Universidade Federal De São Paulo,

**Introduction:** CTEPH is a complex disease characterized by two vascular lesions, organized chronic thrombi and arteriopathy, with functional, tomographic, and hemodynamic repercussions of variable, sometimes discordant spectra. Ventilatory and gas exchange features are notorious in CTEPH, but the association with tomographic and hemodynamic changes is still poorly studied.

**Objectives:** To identify phenotypes among CTEPH patients, considering findings at CT pulmonary angiography (CTPA) and functional and hemodynamic features through cluster analysis; to evaluate the association of phenotypes with ventilatory and gas exchange alterations at the incremental cardiopulmonary exercise testing (CPET).

**Methods:** This was a comprehensive retrospective, cross‐sectional, single‐center study. We evaluated consecutive CTEPH patients who underwent echocardiography, CTPA, right heart catheterization (RHC), and CPET at baseline. We used RHC variables (RAP, mPAP, SvO2, PVR, CI, SVi), Qanadli index (%), and mosaic pattern quantification to identify clusters.

**Results:** 66 CTEPH patients were evaluated. Three different clusters were identified, each with unique functional, hemodynamic, and tomographic features. Cluster 1 was hemodynamically more severe (SvO2 50,5 ± 8,8; 58,7 ± 8,3; 64,6 ± 6,9; P Value < 0,001) (PVR dyn.s.cm5 1309 ± 487; 826 ± 343; 666 ± 299 P Value < 0,001), with a greater absolute Qnadilli score (22 ± 8; 15 ± 8; 13 ± 6; P value 0,003); Cluster 2 was intermediate, and Cluster 3 was the mildest. The mosaic pattern differentiated the groups, with cluster 1 showing more mosaic attenuation, and cluster 3 showing less. Importantly, excessive ventilation response to exercise was associated with these clusters (Slope ΔV'E/ΔV'CO2 67,4 ± 18,5; 56,5 ± 16,1; 51,3 ± 11,5; P value = 0.01) respectively to clusters 1, 2, and 3). In conclusion, CTEPH can be differentiated into clusters considering hemodynamic and imaging data, with different phenotypes associated with excessive ventilatory response to exercise.

## 70. MPAP/VO2‐Slope as Non‐Invasive Surrogate of Cardiopulmonary Exercise Hemodynamics and It's Impact on Prognosis

John T^1^, Ablen H^1^, Foris V^1^, John N^1^, Zeder K^1^, Kneidinger N^1^, Olschewski H^1^, Kovacs G^1^, Douschan P^1^

^1^Medical University of Graz, Graz, Austria

**Background:** Exercise pulmonary hypertension (EPH), defined by a mean pulmonary arterial pressure (mPAP)/cardiac output (CO) slope > 3 mmHg/L/min, is associated with poor prognosis. Its diagnosis relies on invasive assessment of pulmonary exercise hemodynamics using right heart catheterization (RHC). Non‐invasive assessment by exercise‐echocardiography (ex‐TTE) is limited by an inaccurate estimation of CO. Therefore, we aimed to investigate the utility of a novel non‐invasive exercise‐echocardiographic surrogate (mPAPecho/VO2‐slope) of pulmonary exercise hemodynamics and its prognostic relevance.

**Methods:** Patients at risk for pulmonary hypertension (PH) who underwent invasive exercise RHC and combined cardiopulmonary‐exercise‐testing (CPET) with ex‐TTE within the same work‐up period were included. CPET derived oxygen‐uptake (VO2) as surrogate for CO and exercise echocardiography derived mPAP (mPAPecho) were used to calculate mPAPecho/VO2‐slopes. The association of VO2 with CO and the association of mPAPecho/VO2‐slope with invasive mPAP/CO‐slope were assessed. Receiver operating characteristic (ROC) were used to identify the best cut‐offs of mPAPecho/VO2‐slope to detect EPH and to predict clinical worsening (hospitalization/death). Survival analyses were performed using Kaplan‐Meier‐curves and log‐rank tests.

**Results:** N = 69 patients (54 ± 13 yrs, n = n = 54 (79%) female), median observational time: 145 (31‐167) months) investigated between 2006 and 2023 were retrospectively analyzed. Exercise performance was well preserved in the cohort (peakVO2: 84 ± 24%predicted). Resting‐ (ρ = 0.68; p = < 0.001)‐ and peakVO2 (ρ = 0.69; p = < 0.001) were significantly correlated to invasive resting‐ and peak CO, respectively. Non‐invasive mPAPecho/VO2‐slope was significantly correlated to invasive mPAP/CO‐slope (ρ = 0.62; p < 0.001). The best mPAPecho/VO2‐slope cut‐off to predict EPH was 13.9 mmHg/L/min (AUC 0.85, 95%CI (0.74 – 0.96), sensitivity:0.83, specificity:0.89). Moreover, a mPAPecho/VO2‐slope ≥ 11.1 mmHg/L/min was associated with poor prognosis (log‐rank, p = 0.036).

**Conclusion:** MPAPecho/VO2‐slope turned out as promising non‐invasive surrogate of invasive pulmonary exercise hemodynamics. Moreover, it may serve as prognosticator in patients at risk for PH. Larger prospective studies are needed to confirm the clinical and diagnostic relevance of mPAPecho/VO2‐slope.

## 71. Endothelial Cells in Chronic Thromboembolic Pulmonary Hypertension Exhibit Cytoskeletal Alterations

Peracaula M^1^, Gratacós M^1^, Martínez‐Blanco Á^2^, Gavara N^2^, König J^3,4,5^, Luque N^1^, Poyatos P^1^, Peinado V^6,7,8^, Castellà M^9^, Blanco I^7,8^, Szulcek R^3,4,5,10^, Barberà J^7,8^, Tura‐Ceide O^1,7,8,11^

^1^Translational Research Group on Cardiovascular Respiratory Diseases (CAREs), Dr. Josep Trueta University Hospital de Girona, Santa Caterina Hospital de Salt and the Girona Biomedical Research Institute (IDIBGI‐CERCA), 17190 Girona, Spain., ^2^Unitat de Biofísica i Bioenginyeria, Facultat de Medicina i Ciències de la Salut, Universitat de Barcelona, Barcelona, Spain., ^3^Laboratory of in vitro modeling systems of pulmonary and thrombotic diseases, Institute of Physiology, Charité – Universitätsmedizin Berlin, corporate member of Freie Universität Berlin and Humboldt‐Universität zu Berlin, Charitéplatz 1, 10117 Berlin, Germany., ^4^DZHK (German Centre for Cardiovascular Research), partner site Berlin, Berlin, Germany., ^5^DZL (German Centre for Lung Research), partner site Berlin, Berlin, Germany., ^6^Department of Experimental Pathology, Institut d'Investigacions Biomèdiques de Barcelona (IIBB), CSIC‐IDIBAPS, Rosselló, 161, 08036, Barcelona, Spain., ^7^Department of Pulmonary Medicine, Hospital Clínic‐Institut d'Investigacions Biomèdiques August Pi I Sunyer (IDIBAPS), University of Barcelona, Villarroel, 170, 08036, Barcelona, Spain., ^8^Biomedical Research Networking Centre on Respiratory Diseases (CIBERES), 28029, Madrid, Spain., ^9^Department of Cardiovascular Surgery, Cardiovascular Institute, Hospital Clínic, University of Barcelona, Barcelona, Spain., ^10^Deutsches Herzzentrum der Charité, Department of Cardiac Anesthesiology and Intensive Care Medicine, Augustenburger Platz 1, 13353 Berlin, Germany., ^11^Department of Biological Sciences, Faculty of Science, University of Girona, 17003 Girona, Spain.,

**Introduction:** Endothelial dysfunction is indicated in the development of chronic thromboembolic pulmonary hypertension (CTEPH). Cytoskeletal elements, particularly actin microfilaments and microtubules are crucial for maintaining endothelial plasticity. We hypothesize that CTEPH endothelial cells (ECs) carry specific alterations in their cytoskeletal components that impact structural and functional integrity of the endothelium.

**Purpose:** The study aimed to investigate cytoskeletal changes in CTEPH‐ECs compared to healthy‐ECs. We characterize morphological and structural differences, focusing on cell size, shape, and cytoskeletal organization. Additionally, we assess the expression of key cytoskeletal components, specifically microtubules and microfilaments, and their influence on ECs migratory capacity.

**Methods:** CTEPH‐ECs were isolated from pulmonary endarterectomy specimens and commercial human pulmonary artery ECs (HPAE‐ECs) were used as healthy‐controls. We employed fluorescence immunocytochemistry to analyze cytoskeletal characteristics. X‐ray microscopy (TXM) evaluated the organization and distribution of cytoskeletal components. Migration assays were conducted to associate the functional impact of structural differences with ECs migration.

**Results:** CTEPH‐ECs were on average larger (1352vs.662 µm², p < 0.01) and more stellate (0.3649vs.0.2887 score, p < 0.01) compared to control‐ECs. Immunostaining revealed higher tubulin (3327vs.2238 AU, p < 0.01) and F‐actin expression (2068vs.1300 AU, p < 0.01) but reduced G‐actin levels (3002vs.7461 AU, p < 0.01). Additionally, CTEPH microfilaments and microtubules were thinner (0.6219vs.0.8311 AU, p < 0.01) (0.3407vs.1.371 AU, p < 0.01) and less aligned (0.1325vs.0.1558 index, p < 0.01) (0.109vs.0.141, p < 0.01). CTEPH‐ECs exhibited impaired wound healing after 24 h (60vs.89% closure, p < 0.05), with increased but irregular microtubule and microfilament expression during healing. TXM technology images showed a less compact arrangement of microtubules in CTEPH‐ECs (4.2×10⁷vs. 8.7×10⁶nm³, p < 0.05).

**Conclusion:** These findings highlight significant structural, compositional and spatial organization differences in CTEPH‐ECs compared to control‐ECs, alongside altered wound healing capacity. Insights into the cytoskeletal dynamics in CTEPH may provide potential therapeutic targets and a deeper understanding of CTEPH pathophysiology.

## 72. The Role of SMYD2/RUNX2 Axis in Right Ventricular Hypertrophy and Failure

Sapehia V^1^, Weismann N^1^, Seeger W^1^, Novoyatleva T^1^, Schermuly R^1^

^1^Jlu Giessen, Giessen, Deutschland, ^2^Cardio‐Pulmonary Institute (CPI), Universities of Giessen and Marburg‐Lung‐Center (UGMLC), Justus‐Liebig‐University, Aulweg 130, 35392, Giessen, Germany, Giessen, Deutschland

Pulmonary hypertension (PH) is a life‐threatening disease, characterized by excessive pulmonary vascular remodeling, leading to elevated pulmonary arterial pressure and right ventricular hypertrophy (RVH).The RV is the major determinant of functional state and prognosis in PH.RVH triggered by pressure overload is initially compensatory but ultimately leads to RV failure.The mechanisms underpinning the development of RV failure are still unexplored. Recent studies demonstrated that an osteogenic transcription factor Runt‐related transcription factor‐2 (RUNX2) plays a pathogenic role in cardiac hypertrophy and failure,and contributes to the development of PH.In this study, we hypothesize the existence of the interplay of the methyl transferase SMYD2 (SET and MYND domain‐containing protein 2) and RUNX2 in RVH. We found that Smyd2 negatively affects RUNX2 protein expression and it appeared as a key player in RUNX2 methylation,confirmed by in vitro methyl transferase assay. Here,we mechanistically demonstrated that Smyd2 and RUNX2 interact physically and functionally. Co‐Immunoprecipitation studies revealed that endogenous and overexpressed Smyd2 protein physically interact with RUNX2 protein. Additionally, the impact of gene silencing and overexpression studies of RUNX2 and Smyd2 over one another was analyzed using primary cardiac fibroblasts (CFs) by employing adenovirus‐mediated and si‐RNA‐mediated overexpression and gene silencing approaches respectively. In loss‐of‐function studies RUNX2 silencing reduced CF proliferation and promoted the down regulation of mRNA expression of osteogenic genes as Col1α1, Col3α1, alkaline phosphatase (ALP) and cartilage oligomeric matrix protein (COMP). Notably, Smyd2 knockdown provided opposite effects in CFs. In our experiments, we observed the negative effects of Smyd2 ectopic expression on RUNX2 protein accumulation in CFs. Furthermore,Smyd2 significantly impeded the expression of osteogenic markers such as Col1α1, Col3α1, ALP and COMP both in human and rat CFs. Thus, the main conclusion of our study is that Smyd2 plays a negative role in osteogenesis by suppressing the osteogenic gene program and inhibiting the expression of RUNX2.

## 73. Piezo1 in Pulmonary Arterial Smooth Muscle Cells – A Critical Player for the Development of Hypoxia‐Induced Pulmonary Hypertension

Gökyildirim M^1,2^, Abid S^3^, Houssaini A^3^, Lipskaia L^3^, Born E^3^, Marcos E^3^, Arhatte M^4^, Glogowska E^4^, Vienney N^3^, Günther A^2^, Kraut S^2^, Quanz K^2^, Fenner‐Nau D^2^, Derumeaux G^3^, Weissmann N^2^, Honoré E^3^, Adnot S^1,3^, Knoepp F^2^

^1^Institute for Lung Health (ILH), Justus Liebig University, Giessen, Deutschland, ^2^Excellence Cluster Cardio‐Pulmonary Institute (CPI), Universities of Giessen and Marburg Lung Center, Member of the German Center for Lung Research, Justus Liebig University, Giessen, Deutschland, ^3^INSERM U955, Département de Physiologie‐Explorations Fonctionnelles and FHU SENEC Hôpital Henri Mondor, AP‐HP, Créteil, France, ^4^Université Côte d'Azur, Centre National de la Recherche Scientifique, Institut national de la santé et de la recherche médicale, Institut de Pharmacologie Moléculaire et Cellulaire, Labex ICST, Valbonne, France

Pulmonary hypertension (PH) is a life‐threatening and progressive, but yet incurable disease. The hallmarks of PH comprise sustained contraction and excessive proliferation of pulmonary arterial smooth muscle cells (PASMCs). A major stimulus to which PASMCs are exposed during PH development is altered mechanical stress. Mechanosensitive ion channels, such as Piezo1, perceive such mechanical stimuli and translate them into various cellular responses. Thus, the objective of the present study was to elucidate the specific role of Piezo1 in PASMCs for PH‐development and progression.

Taking advantage of PASMCs from idiopathic pulmonary arterial hypertension (IPAH)‐patients and two mouse strains characterized by SMC‐specific Piezo1 knock‐out, we assessed the SMC‐specific role of Piezo1 in PH‐development and progression via experiments in isolated, perfused and ventilated mouse lungs, wire myography and proliferation assays. In vivo function of SMC‐specific Piezo1 knockout was evaluated upon induction of chronic hypoxia‐induced PH (CHPH) with insights into pulmonary vascular cell senescence.

Compared to healthy controls, PASMCs from PH‐patients featured an elevated Piezo1‐expression and increased proliferative phenotype. SMC‐specific Piezo1‐deletion, as confirmed via qPCR and patch clamp recordings, prevented the hypoxia‐induced increase in PASMC‐proliferation in mice. Moreover, Piezo1‐knockout reduced hypoxic pulmonary vasoconstriction (HPV) in isolated, perfused and ventilated mouse lungs, endothelial‐denuded pulmonary arteries and hemodynamic measurements in vivo. Consequently, Piezo1‐deficient mice were considerably protected against CHPH‐development with ameliorated right heart hypertrophy and improved hemodynamic function. In addition, distal pulmonary capillaries were preserved in the Piezo1‐knockout mice, associated with a lower number of senescent endothelial cells.

This study provides evidence that Piezo1 expressed in PASMCs is critically involved in the pathogenesis of PH ‐ by controlling pulmonary vascular tone, and arterial remodeling, and associated lung capillary rarefaction due to endothelial cell senescence.

## 74. Association Between Predicted Cardiac Age and Right Ventricular Dysfunction Based on Electrocardiogram Artificial Intelligence in Acute Pulmonary Embolism

Wang D^1^, Zhang X^1^, Geng S^2^, Fan G^1^, Xu F^1^, Hong S^2^, Zhai Z^1^

^1^China‐Japan Friendship Hospital, China, ^2^Peking University Health Science Center, China

**Purpose:** We aimed to evaluate the prediction value of electrocardiographic heart age (ECG‐age) by applying deep neural network on right ventricular dysfunction (RVD) in patients with pulmonary embolism (PE).

**Methods:** The ECG‐age prediction model, built on RegNet and trained using mean squared error (MSE), was developed with 21,799 ECG recordings from 18,869 patients in mimic dataset and had been validated on external test sets previously. In this study,the model was applied to the biological age among patients with PE diagnosed hospitalizied in China‐Japan Friendship Hospital between 2016‐2022. The difference between the ECG‐age and biological age (Δage) of each patient was calculated. Correlation and logistic analyses were conducted to investigate the associations between Δage group and echocardiographic parameters on RVD and pulmonary circulation.

**Results:** 86 data pairs of 74 patients with contemporaneous (intervals of examinations dates less than 7 days) ECG signal and echocardiography were included. The median biological age was 69 (57,77) years and median ECG‐age 57 (49,60) years. The median Δage was ‐9.5 ± 12.2 years. Δage was significantly associated with tricuspid regurgitation velocity, systolic pulmonary arterial pressure, main pulmonary artery diameter and thickness of interventricular septum (R = 0.38, 0.35, 0.34, 0.25, respectively). The prevalence of RVD Δage ≥ 0 and < 0 grouop were 6(25%) and 9(18.75%), respectively(P = 0.76). logistic regression revealed Δage ≥ 0 and biological aging were risk factors for RVD [adjusted ORs 8.39 (1.31,53.58) and 1.08(1.02,1.14), respectively].

**Conclusions:** The increased difference between ECG‐age and biological age was associated with RVD and other parameters of pulmonary circulation. Given the wide availability and low cost of ECG, ECG‐age has the potential to be a new biomarker of risk classification and prognosis in PE patients.

## 75. Real‐World Dosing of Tyvaso Dpi in Pulmonary Arterial Hypertension and Pulmonary Hypertension Associated With Interstitial Lung Disease

Parikh R^1^, El‐Kersh K^2^, Wu B^3^, Thrasher C^3^, Broderick M^3^, Burger C^4^

^1^Hartford Hospital, Hartford, United States, ^2^University of Arizona, Phoenix, United States, ^3^United Therapeutics Corporation, Research Triangle Park, United States, ^4^Mayo Clinic, Jacksonville, United States

**Background:** Treprostinil dry powder inhaler (DPI) is indicated to improve exercise capacity in pulmonary arterial hypertension (PAH) and pulmonary hypertension associated with interstitial lung disease (PH‐ILD). Inhaled treprostinil should be titrated to a maximally tolerated dose to maximize benefit from its dose‐related effects. This analysis characterizes real‐world dosing of treprostinil DPI in patients with PAH and PH‐ILD.

**Methods:** Using specialty pharmacy data, a random sample was generated of patients who received their first treprostinil DPI shipment between 5/25/22‐4/16/24. Patients were stratified 1:1 by indication (PAH, PH‐ILD) and start type (“Transition” from nebulized treprostinil and “De novo” with no prior treprostinil shipment). Dosing was assessed in two‐month intervals to account for titration variation and is reported as equivalent breaths of nebulized treprostinil (4x daily). If multiple shipments were received in one month, the highest dose was reported; invalid doses (missing, zero, not an integer of 16) were recalculated using cartridge strength and quantity.

**Results:** 700 PAH and 700 PH‐ILD patients were included, each with 350 De novo and 350 Transitions. By month two, the median (IQR) dose for both indications (PAH, PH‐ILD) and both start types (Transition, De novo) was 12 (9, 12) breaths, which was maintained for all groups up to month 12. The maximum doses were 27 breaths for PH‐ILD Transitions, 24 breaths for PH‐ILD De novo and PAH Transitions, and 21 breaths for PAH De novo.

**Conclusions:** A limitation of specialty pharmacy records is the lack of patient information available to further characterize the sample population. In general, patients with PAH and PH‐ILD achieved a median treprostinil DPI dose of 12 breaths within two months regardless of starting de novo or transitioning. Maximum doses achieved were similar between de novo and transition, and between indications, demonstrating that treprostinil DPI can be titrated effectively in PAH and PH‐ILD.

## 76. Isolated Ventilated and Perfused Mouse Lungs — A Novel Tool to Study Immune Cell Recruitment in The Development of Copd‐Associated Pulmonary Hypertension

Sharma V^1,2^, Pes K^1,2^, Pak O^1,2^, Sommer N^1,2^, Seeger W^1,2,3^, Weissmann N^1,2^, Gredic M^1,2^

^1^Cardio‐Pulmonary Institute (CPI), Universities of Giessen and Marburg Lung Center (UGMLC), Member of the German Center for Lung Research (DZL), Giessen, Germany, ^2^Institute for Lung Health (ILH), Justus‐Liebig‐University, Giessen, Germany, ^3^Max Planck Institute for Heart and Lung Research, Bad Nauheim, Germany

The isolated ventilated and perfused mouse lung (ILU) model has previously been used in our laboratory to study the mechanisms of hypoxic pulmonary vasoconstriction. We recently adapted it for intratracheal cigarette smoke (CS) exposure to investigate early molecular changes in COPD. However, the inflammatory component—critical for the development of both pulmonary hypertension and emphysema—was missing. In this study, we aimed to modify the ILU model to study immune cell recruitment from circulation to lung tissue in response to stimuli such as CS inhalation.

We introduced in vitro‐differentiated bone marrow‐derived macrophages or freshly isolated bone marrow monocytes into the ILU perfusate. The cells were labeled with viable cell dyes or the fluorescent protein tdTomato. Lung morphology and immune cell presence were analyzed in immunostained cryosections.

Our experiments showed that the use of mature macrophages and commercially available viable cell dyes caused the occlusion of pulmonary capillaries. The inability of macrophages and dyed monocytes to circulate freely through ILU system resulted in their absence from the perfusate at later time points, an increase in pulmonary arterial pressure (PAP), and distorted morphology of these cells and capillaries in the ILU cryosections. Interestingly, tdTomato‐expressing monocytes, freshly isolated from bone marrow, circulated without affecting PAP. The majority remained in the perfusate throughout the experiment, while those recruited to the lungs were found outside capillaries with normal morphology. Recruitment was more prominent in lungs repeatedly intratracheally exposed to CS.

Overall, we developed a model that enables the investigation of monocyte recruitment to the lungs in response to inhalatory challenges such as CS. Since the model can be employed to investigate the recruitment of other immune cells and in response to different stimuli, its application can be extended to study various inflammation‐driven lung pathologies.

## 77. Bakuchiol: Repurposing Anti‐Aging Skincare for the Treatment of Pulmonary Hypertension

Hoffmann F^1,2^, Pes K^1,2^, Pak O^1,2^, Sommer N^1,2^, Seeger W^1,2,3^, Weissmann N^1,2^, Gredic M^1,2^

^1^Cardio‐Pulmonary Institute (CPI), Universities of Giessen and Marburg Lung Center (UGMLC), Member of the German Center for Lung Research (DZL), Giessen, Germany, ^2^Institute for Lung Health (ILH), Justus‐Liebig‐University, Giessen, Germany, ^3^Max Planck Institute for Heart and Lung Research, Bad Nauheim, Germany

Pulmonary hypertension (PH) is characterized by elevated blood pressure in the pulmonary arteries, leading to increased right ventricular workload and potential heart failure. The molecular mechanisms underlying PH pathogenesis include excessive pulmonary vasoconstriction, inflammation, and abnormal vascular cell growth, driven by several pro‐proliferative signaling pathways, such as growth factors, cytokines, metabolic changes, and tissue remodeling enzymes.

Bakuchiol (BAK) is a natural compound derived from the seeds and leaves of Psoralea corylifolia, known for its anti‐proliferative, anti‐inflammatory, and antioxidant properties in vitro and in vivo. However, its effects on pulmonary vasculature and PH remain unexplored.

In this study, we utilized an ex vivo isolated ventilated and perfused mouse lung model to assess the effects of BAK on hypoxic pulmonary vasoconstriction (HPV). Additionally, we conducted various cell‐based assays to evaluate the effects of BAK on the functional properties of pulmonary artery smooth muscle cells (PASMCs) in the presence of PDGF‐BB, a growth factor implicated in pulmonary vascular remodeling in PH.

BAK administration significantly reduced the acute rise in pulmonary arterial pressure induced by hypoxic ventilation, indicating an inhibitory effect on HPV‐induced vasoconstriction. Notably, BAK did not influence pulmonary vasoconstriction triggered by the thromboxane analog U46619. Cell culture experiments demonstrated that BAK attenuates PDGF‐BB‐induced proliferation and migration of PASMCs while moderately promoting apoptosis in these cells. Interestingly, the same concentration of BAK increased both proliferation and apoptosis in control PASMCs, likely resembling its reported effects on skin epithelium turnover.

Overall, our results suggest that BAK inhibits hypoxia‐induced pulmonary vasoconstriction and reduces the excessive proliferation and migration of PDGF‐BB‐treated PASMCs in vitro. Further studies in relevant animal models are warranted to explore the therapeutic potential of BAK for treating PH.

## 78. Impact of IRG1/Itaconate Axis on Cardiopulmonary Phenotype: Linking Bone Marrow Dynamics to Inter‐Organ Communication

Hesami G^1^, Mansouri S^1^, Schmachtel T^3^, Valasarajan C^1^, Olapoju S^1^, Maroli G^1^, Kojonazarov B^1^, Weigert A^4^, Günther S^2^, Seeger W^1^, Rieger M^3^, Savai R^1^, Savai Pullamsetti S^1^

^1^Universities of Giessen and Marburg Lung Center (UGMLC), Institute for Lung Health (ILH), Cardio‐Pulmonary Institute (CPI), Member of the German Center for Lung Research (DZL), Giessen, Germany, ^2^Max Planck Institute for Heart and Lung Research, Bad Nauheim, Germany, ^3^Department of Medicine, Hematology/Oncology Goethe University Hospital, Frankfurt, Germany, ^4^Institute of Biochemistry I, Goethe‐University Frankfurt, Frankfurt, Germany

Pulmonary hypertension (PH) is a progressive and lethal cardiopulmonary disorder characterized by pulmonary vascular remodeling and right ventricular hypertrophy. Bone marrow (BM)‐derived hematopoietic cells, particularly myeloid cells contribute to the process of pulmonary vascular remodeling. Immune‐Responsive Gene 1 (IRG1) encodes cis‐aconitate decarboxylase enzyme, responsible for producing itaconate in macrophages in inflammatory phase. However, the association of IRG1/itaconate axis with the cardiovascular system and PH progression remains largely unknown. Here, we reveal that Irg1 − /− mice exhibit baseline pulmonary vascular remodeling, cardiac hypertrophy and dysfunction which are exacerbated by hypoxic exposure. Immune profiling revealed the infiltration of macrophages and dendritic cells into the lung and heart of Irg1 − /− mice. Adoptive transfer of BM from Irg1‐deficient mice into naïve WT mice derives cardiac hypertrophy, emphasizing the strong contribution of BM to cardiac dysfunction under Irg1 deficiency. We assessed the multipotency of hematopoietic stem progenitor cells (HSPCs), serving as immune cells progenitor in Irg1 − /− mice. These mice exhibited a reduction in long‐term (LT) HSCs, alongside a pronounced shift towards lymphoid‐primed multipotent progenitors (MPP‐Ly). HSPCs from Irg1 − /− mice exhibited differential transcriptomic activation of immunometabolic pathways, with significant enrichment in NLRP3 inflammasome, complement system activation, and purine metabolism pathways, coupled with a dysregulated metabolomic profile. Notably, the immunometabolic modulatory effect of itaconate was investigated in vitro and in vivo, where 4‐octyl‐itaconate (4‐OI) treatment diminishes the proliferation of pulmonary vascular cells from human PH patients. Moreover, administration of 4‐OI in a therapeutic approach attenuates monocrotaline‐induced PH in rats. In conclusion, our findings indicate that IRG1/itaconate axis deficiency drives cardiopulmonary phenotype by dysregulating HSPC differentiation and mobilization through immunometabolic alterations, ultimately leading to pulmonary vascular remodeling and cardiac hypertrophy. PH phenotype could be mitigated through itaconate administration, highlighting its potential as a therapeutic strategy for PH and related cardiac complications.

## 79. Sulfatase‐1 Modulates Pulmonary Endothelial Cell Migration Through Podosome Rosettes Formation to Promote Pulmonary Arterial Hypertension

Samokhin A^1^, Saha P^1^, Daum J^1^, Maron B^1,2^

^1^University Of Maryland, Baltimore, Baltimore, USA, ^2^The University of Maryland‐Institute for Health Computing, Bethesda, USA

**Introduction:** Endothelial glycocalyx is a gel‐like structure covering luminal surface of endothelial cells and its disruption is associated with pulmonary arterial hypertension (PAH). Sulfation of glycocalyx‐forming heparan sulfate proteoglycans affects many properties of endothelial cells, including adhesion and cell migration. Sulfatase‐1 (SULF1) is an enzyme that removes 6‐O‐sulfate from heparan sulfate and we recently demonstrated that SULF1 is upregulated in endothelium of pulmonary arterioles from patients with PAH and two rodent PAH models. In turn, SULF1 inhibition in vivo improved cardiopulmonary hemodynamics and fibroproliferative vascular remodeling. We suggested that the effect of SULF1 on PAH development might be mediated through endothelial cell migration and the formation of podosomes, which are extracellular matrix degrading and adhesive structures.

**Methods:** Migration of human pulmonary artery endothelial cells (HPAECs) from PAH patients was compared to healthy controls in wound healing assay. The effect of SULF1 on the migration of HPAECs from healthy controls was analyzed in response to adenovirus‐mediated overexpression or inhibition of SULF1. Podosomes were detected by immunostaining for cortactin and phosphorylated focal adhesion kinase.

**Results:** HPAECs from PAH patients showed significantly lower rates of migration compared to healthy controls (wound size 499 ± 66 vs. 318 ± 45 μm, P < 0.001). Similarly, SULF1 overexpression resulted in slower migration, whereas SULF1 inhibition increased it compared to adenovirus control (wound size 689 ± 35 vs 336 ± 28 vs. 461 ± 39 μm, respectively, P < 0.001). The number of cells with podosome rosettes, circular structures formed by individual podosomes, was decreased by SULF1 overexpression and increased by SULF1 inhibition (2.7 ± 0.5 vs 16.7 ± 2.7 vs 5.5 ± 1.2%, respectively, P < 0.001).

**Conclusions:** SULF1 negatively regulates HPAECs migration by reducing the number of cells with high extracellular matrix‐degrading potential attributed to podosome rosettes. Identifying podosome regulation by SULF1 may have important therapeutic implications for PAH.

## 80. Immune Profiling of Peripheral Blood Mononuclear Cells of Schistosoma‐Induced Pulmonary Hypertension by Mass Cytometry

Mickael C^1^, Borges V^2^, de Paula Alves M^2^, Arroyo Rodrigues B^3^, K. F. Oliveira R^4^, M. C. Loureiro C^5^, F. Hilton J^6^, T. Zamanian R^3^, Stenmark K^1^, A. Correa R^2^, B. Graham B^6^

^1^University Of Colorado, Aurora, United States, ^2^Federal University of Minas Gerais, Belo Horizonte, Brazil, ^3^Stanford University, Stanford, United States, ^4^Federal University of Sao Paulo, Sao Paulo, Brazil, ^5^Santa Casa da Bahia, Salvador, Brazil, ^6^University of California San Francisco, San Francisco, United States

**Background:** Schistosoma‐induced pulmonary arterial hypertension (Sch‐PAH) is a prevalent but underexplored form of PAH, affecting approximately 5% of patients with chronic S. mansoni infections leading to Schistosoma hepatosplenic disease (Sch‐HSD). The progression to Sch‐PAH is likely due to portal hypertension and chronic inflammation, which create vascular shunts that transport Schistosoma eggs into the pulmonary circulation, causing inflammation. Sch‐PAH features remodeled vessels with inflammatory cell accumulation in the adventitia, critical to disease pathogenesis. This study aims to characterize the circulatory immune profile in patients with Sch‐HSD and Sch‐PAH to identify differences in peripheral inflammatory cells.

**Methods:** Peripheral blood mononuclear samples were collected from patients with Sch‐HSD, Sch‐PAH, and healthy controls (HCs) and analyzed using multi‐parameter mass cytometry time of flight (CyTOF) with a panel of 28 antibodies for cell identification and phenotyping.

**Results:** Four Sch‐PAH, five Sch‐HSD, and seven HCs were analyzed. CD4 + T cells, especially Tregs, were reduced in both Sch‐HSD and Sch‐PAH compared to HCs. Both groups showed fewer naïve and Th1 CD4+ and CD8 + T cells. Conversely, CD1c+ dendritic cells and intermediate monocytes were elevated in Sch‐HSD and Sch‐PAH. The Sch‐HSD group had an increased number of double‐positive CD4 CD8 T cells and activated T cells, along with two CD8+ subsets—effector CD8+ and Th1‐like CD8 + T cells that were elevated only in Sch‐HSD. Plasmacytoid DCs, known for type I interferon production, were elevated only in the Sch‐PAH group.

**Conclusions:** Despite both being chronic forms of Schistosomiasis, Sch‐HSD and Sch‐PAH exhibit distinct immune profiles. Key differences include increased plasmacytoid DCs in Sch‐PAH and higher double‐positive CD4‐CD8 T cells and effector CD8 T cells in Sch‐HSD, which may relate to disease pathogenesis.

## 81. Breeze Optional Extension Phase: Long‐Term Outcomes With Tyvaso DPI in Patients With Pulmonary Arterial Hypertension

Spikes L^1^, Burger C^2^, Desai S^3^, Eggert M^4^, El‐Kersh K^5^, Fisher M^6^, Johri S^7^, Joly J^8^, Mehta J^9^, Palevsky H^10^, Ramani G^11^, Restrepo‐Jaramillo R^12^, Sahay S^13^, Shah T^14^, Shapiro S^15^, Thrasher C^16^, Deng C^16^, Smith P^16^, Shen E^16^, Broderick M^16^, Bajwa A^2^

^1^University of Kansas, Lawrence, United States, ^2^Mayo Clinic, Jacksonville, United States, ^3^Ochsner Medical Center, New Orleans, United States, ^4^Sentara Health, Norfolk, United States, ^5^University of Arizon, Phoenix, United States, ^6^Emory University, Atlanta, United States, ^7^Pulmonary Associates of Richmond, Richmond, United States, ^8^University of Alabama, Birmingham, United States, ^9^Cleveland Clinic, Weston, United States, ^10^University of Pennsylvania, Philadelphia, United States, ^11^University of Maryland, Baltimore, United States, ^12^University of South Florida, Tampa, United States, ^13^Houston Methodist, Houston, United States, ^14^University of Texas Southwestern, Dallas, United States, ^15^University of California Los Angeles, Los Angeles, United States, ^16^United Therapeutics Corporation, Research Triangle Park, United States

**Purpose:** The BREEZE study demonstrated the safety and tolerability of treprostinil inhalation powder (Tyvaso DPI) in patients with pulmonary arterial hypertension (PAH). The optional extension phase (OEP) aimed to assess long‐term safety, tolerability, and clinical outcomes.

**Methods:** Patients on stable doses of nebulized treprostinil transitioned to corresponding doses of Tyvaso DPI four times daily (QID). After a 3‐week treatment phase, patients could enter the OEP, where Tyvaso DPI was titrated to a maximally tolerated dose. Safety, 6‐minute walk distance (6MWD), Preference Questionnaire for Inhaled Treprostinil Device (PQ‐ITD), and PAH Symptom Impact (PAH‐SYMPACT) were assessed at baseline and throughout the OEP. Here we present the results seen at week 107 and week 131, reported as mean ± SD.

**Results:** Of the 51 patients enrolled, 49 completed the treatment phase and entered the OEP (55% idiopathic PAH, 29% CTD‐PAH, 84% on dual‐therapy, 16% monotherapy). Mean time on Tyvaso DPI was 106.3 ± 48.9 weeks; the most common reasons for discontinuation were study conclusion by sponsor (63%), adverse events (14%), and disease progression (10%). Mean dose at study completion was 72 ± 28 mcg QID (13 ± 5 breaths nebulized treprostinil); the highest dose was 176 mcg QID (33 breaths). Mean change in 6MWD was 18 ± 50 meters at Week 51 (n = 38), 15 ± 55 meters at Week 107 (n = 19), and 14 ± 50 meters at Week 131 (n = 16). For the end‐of‐study PQ‐ITD, 87% strongly agreed with the statement, “Overall I'm satisfied with the device”; no patients disagreed with any of the preferred statements around Tyvaso DPI. At study completion, there were no significant changes in PAH‐SYMPACT scores. Drug‐related AEs were consistent with previous studies of inhaled treprostinil in PAH (14% cough, 14% headache, 12% dizziness, 12% dyspnea); no serious AEs occurred.

**Conclusions:** For patients with PAH, Tyvaso DPI is a safe, tolerable, and preferred treatment option that may provide long‐term clinical stability.

## 82. Safety and Tolerability of Treprostinil Palmitil Inhalation Powder (TPIP) in Pulmonary Hypertension Associated With Interstitial Lung Disease (PH‐ILD)

Molina‐Molina M^1^, Arias‐Guillén M^2^, Church C^3^, Makulova N^4^, Ismat F^4^, Mistry B^4^, Guo J^4^, Fahumy J^4^, Teper A^4^, Zhao P^4^, Mange K^4^, O'Brien G^4^

^1^Interstitial Lung Disease Unit, Respiratory Department, University Hospital of Bellvitge, Research Lab 4126, IDIBELL, UB, Barcelona, Spain, ^2^Interstitial Lung Disease Unit, Respiratory Department, Central University Hospital of Asturias, ISPA, UO.CIBERES (ISCIII), Oviedo, Spain, ^3^Golden Jubilee National University Hospital, Glasgow, UK, ^4^Insmed Incorporated, Bridgewater, USA

**Objective:** This phase 2, double‐blind, placebo‐controlled trial (NCT05176951) evaluated safety and tolerability of TPIP in patients with PH‐ILD over 16 weeks.

**Methods:** Patients were randomized 3:1 to TPIP or placebo dry powder inhalation once‐daily, titrated from 80 μg to the maximum tolerated dose, up to 640 μg. Primary endpoints were adverse events (AEs), serious AEs (SAEs), and change in oxygenation during 6‐min walk test (6MWT). Secondary endpoints (pharmacokinetics [PK]) and exploratory endpoints were assessed.

**Results:** 39 patients were randomized (TPIP n = 29; placebo n = 10); 30 (76.9%) completed treatment. Baseline characteristics were generally balanced except for gender. TPIP maximum dose (640 μg) was achieved by 79.3% of patients. AEs occurred in 27 (93.1%) vs 9 (90.0%) patients with TPIP vs placebo; most frequent were cough, diarrhea, and dyspnea and most AEs were mild/moderate. SAEs occurred in 6 (20.7%) vs 4 (40.0%) patients with TPIP vs placebo; there were 2 deaths in each group. No SAE/death was assessed as treatment‐related. AEs leading to treatment discontinuation were less frequent with TPIP (13.8%) vs placebo (30.0%). There were no meaningful changes from baseline in oxygenation at 6MWT or in supplemental oxygen use with TPIP or placebo. The TPIP PK profile supports once‐daily dosing. At week 10 following TPIP 480 μg and 640 μg, the treprostinil median Tmax, mean Cmax, AUCƬ, and t1/2 were 1.03 and 1.46 h, 1246 and 1594 pg/mL, 4675 and 7510 pg*h/mL and 7.28 and 7.10 h, respectively. Exploratory endpoints: Clinical worsening occurred in 3 (10.3%) vs 5 (50.0%) patients with TPIP vs placebo (nominal P = 0.0164). Placebo‐corrected median change from baseline in the 6MWT distance with TPIP was 30 m (95% CI –49 to 171; nominal P = 0.348).

**Conclusion:** TPIP was generally well tolerated, with the maximum dose (640 μg) achieved by most patients. AEs were consistent with inhaled prostacyclin therapy.

## 83. Novel Insights From Functional Respiratory Imaging (FRI) Analysis in Phase 2 INS1009‐211 Study: Treprostinil Palmitil Inhalation Powder (TPIP) Impact on Pulmonary Vasculature in Patients With Pulmonary Hypertension Associated With Interstitial Lung Disease (PH‐ILD)

Church C^1^, Muchmore P^2^, Lavon B^2^, De Backer J^2^, Makulova N, Ismat F^3^, Guo J^3^, Mange K^3^, O'Brien G^3^

^1^Golden Jubilee National University Hospital, Glasgow, UK, ^2^FLUIDDA, Inc., New York, USA, ^3^Insmed Incorporated, Bridgewater, USA

**Objective:** In a phase 2, double‐blind, placebo‐controlled trial in patients with PH‐ILD (NCT05176951), FRI was used to evaluate the effects of TPIP on pulmonary blood vessel volume and lung parenchyma over 16 weeks.

**Methods:** In this exploratory analysis, FRI parameters were calculated from high‐resolution computed tomography at screening and week 16. Volumes of the macroscopic pulmonary arteries ≤ 5 mm2 (BV5A) and ≥ 10 mm2 (BV10A) in cross‐sectional areas were analyzed and presented as a proportion of total vessel volume (‐PR) and proportion of total arterial volume (‐PRA). The specific image‐based volume of fibrosis (SIVFIB) score, based on deep‐learning algorithms, was defined as fibrotic volume divided by lung volume.

**Results:** 14 patients were included (TPIP n = 9; placebo n = 5). There was a significant fibrosis‐adjusted treatment effect for TPIP vs placebo of 3.8% (P = 0.02) onBV5A‐PR and a borderline significant fibrosis‐adjusted treatment effect for TPIP vs placebo of 6.4% (P = 0.05) onBV5A‐PRA. Unadjusted % change from baseline (CFB) between TPIP vs placebo in BV5A‐PR and BV5A‐PRA was +8.39% vs –5.46% and +8.04% vs –3.75%, respectively. A decrease in the proportion of total blood volume and total arterial volume contained in large arteries (BV10A‐PR and BV10A‐PRA, respectively) was observed with TPIP (–10.80% and –12.03%, respectively) compared to an increase observed with placebo (+16.43% and +18.69%, respectively). The %CFB in the BV5A:BV10A ratio was +31.43% with TPIP vs –10.54% with placebo. There was a numerical decrease from baseline in the SIVFIB score in the TPIP group vs the placebo group (effect size compared to placebo baseline, –10.49%).

**Conclusion:** Treatment with TPIP resulted in changes in blood volume parameters at FRI consistent with peripheral vasodilation and improved pulmonary arteriole recruitment, with an observed numerical decrease in fibrosis score. Further evaluation of FRI in a larger trial may provide additional insights.

## 84. Familial KDR Mutation Resulting in Heritable Progressive Pulmonary Arterial Hypertension

Alba G^1^, Wertheim B^2^, Vargas S^3^, Kennedy J^2,4^, Arons E^2^, Rahaghi F^2^, Estépar R^2^, Garcia‐de‐Alba C^4^, Carmichael N^5^, Hariri L^6^, Rodriguez‐Lopez J^1^, Channick R^7^, Maron B^8^, Raby B^2,4^

^1^Division of Pulmonary and Critical Care Medicine, Massachusetts General Hospital, Boston, USA, ^2^Division of Pulmonary and Critical Care Medicine, Brigham and Women's Hospital, Boston, USA, ^3^Department of Pathology, Boston Children's Hospital and Brigham and Women's Hospital, Boston, USA, ^4^Division of Pulmonary Medicine, Boston Children's Hospital, Boston, USA, ^5^Department of Medical Sciences & Education, Boston University Chobanian & Avedisian School of Medicine, Boston, USA, ^6^Department of Pathology, Massachusetts General Hospital, Boston, USA, ^7^Division of Pulmonary and Critical Care Medicine, University of California Los Angeles, Los Angeles, USA, ^8^Department of Medicine, University of Maryland School of Maryland, Baltimore, MD and the University of Maryland‐Institute for Health Computing, Bethesda, USA

Pulmonary arterial hypertension (PAH) is a progressive pulmonary vasculopathy that may be sporadic or inherited. Vascular endothelial growth factor receptor 2 (VEGFR2), encoded by Kinase insert domain receptor (KDR), plays an essential role in angiogenesis. KDR homozygous loss‐of‐function is embryonic lethal, but heterozygous KDR mutations have been suggested as a possible cause of PAH in genome‐wide association studies and one prior case report. Here, we report on three brothers with autosomal dominant familial PAH caused by heterozygous KDR mutation [c.718 G > T, p.(E240*)] resulting in a translation termination stop codon. The siblings, aged 41 years (median) at presentation without antecedent cardiopulmonary comorbidities, underwent longitudinal clinical phenotyping, including pulmonary function testing, cardiopulmonary exercise testing, chest computed tomography (CT), invasive hemodynamics, and lung biopsy in two of the brothers. In this cohort, KDR mutation was characterized clinically by low DLCO (median 33% predicted), mild centrilobular emphysema on chest CT, and mild precapillary PH with slow progression over 22 person‐years follow up: mean mPAP increase 4.8 ± 0.3 mmHg, PVR increase 0.9 ± 0.2 Wood units, VE/VCO2 slope increase 1.2 ± 1.1, and peak VO2 decrease 0.8 ± 0.7 mL/kg/min. In one brother with serial hemodynamics, there was a strong correlation between age and PVR (r = +0.99, p = 0.04). Lung biopsies from two brothers, sampled at ages 42 and 48, showed chronic small airway remodeling, patchy collections of intra‐alveolar macrophages resembling obstructive sequelae, emphysema (more prominent in the older brother), and pulmonary arteries without appreciable mural thickening. These data suggest heterozygous KDR mutations are associated with diffuse lung disease involving multiple anatomic compartments, including a progressive pulmonary vasculopathy, with a variable clinical phenotype modified by age. Understanding the pathobiology of KDR mutant PAH and its relationship to other forms of PH will broaden our understanding of the heterogenous landscape of pulmonary vascular disease.

## 85. Novel Potential Circulating Biomarkers can Help to Better Characterize Patients With Pulmonary Arterial Hypertension

Bacelar‐Jimenez A^1^, Cruz‐Utrilla A^2,3^, de la Bastida‐Casero L^1^, Navarro‐Llinares L^1^, Sierra‐Palomares Y^1,4^, Escribano P^2,3^, Oliver E^1,3,4^

^1^Centro de Investigaciones Biológicas Margarita Salas (CIB), CSIC, Madrid, Spain, ^2^Hospital Universitario 12 de Octubre, Madrid, Spain, ^3^Centro de Investigación Biomédica en Red Enfermedades Cardiovasculares (CIBERCV), Madrid, Spain, ^4^Centro Nacional de Investigaciones Cardiovasculares (CNIC), Madrid, Spain

Pulmonary Arterial Hypertension (PAH) is a rare and devastating disease characterized by aberrant endothelial dysfunction, inflammation and vascular remodeling leading to increase in pulmonary artery pressure, right ventricle hypertrophy and premature failure. Its origin can be very diverse making precise diagnosis complicated resulting in heterogeneity among patients with significant differences in survival and treatment response. Novel tools allowing better characterization would help to implement precision medicine strategies. Our aim is to study the use of circulating biomarkers mirroring the main hallmarks of the disease. For this purpose, 70 subjects were recruited by Hospital Universitario 12 de Octubre of Madrid. Among them, 52 were PAH patients (n = 19 males(M)/33 females(F)), 14 of them were classified as iPAH, 10 PVOD, 8 congenital, 6 Bmpr2, 5 toxic PAH, 9 with other subtypes and 18 were age‐matched healthy volunteers (n = 6 M/12 F). Plasma samples and demographic data were collected. PAH patients were subjected to deep phenotyping including hemodynamics, echocardiography and cardiac MRI. In total, we have investigated the levels of 24 circulating markers by using Luminex XMAP technology. Normal circulating levels were defined as for plasmatic concentrations in healthy donors. Our results show that type 1 PAH patients have altered plasmatic concentrations markers regarding endothelial dysfunction, inflammation, vascular remodeling and cardiac damage compared to controls. Among them, endothelial damage markers especially correlate with respiratory oxygen uptake (VO2), pulmonary arterial pressure and N‐terminal Brain Natriuretic Peptide levels, confirming their value as potential diagnostic and prognostic biomarkers. Additionally, our results indicate the existence of specific subtype biomarkers. Overall, we can conclude that differences in plasmatic levels of certain soluble proteins such as cytokines, adhesion molecules, angiogenic, extracellular matrix or TGF‐beta related proteins can help to better characterize and discriminate PAH patients. These results could open new avenues towards precision and personalize medicine in PAH.

## 86. Beyond the Trial: Post‐Study Outcomes and Latvian Center Results From the Phase 2b Elevate‐2 Dose‐Ranging, Randomised, Multicentre Trial in Pulmonary Arterial Hypertension

Kaulins R^1^, Rudzitis A^1^, Sablinskis M^1^, Gibietis V^1^, Kigitovica D^1^, Skride A^1^

^1^Rīga Stradiņš University, Riga, Latvia

**Background:** The ELEVATE‐2 clinical trial evaluated the safety and efficacy of rodatristat ethyl, a serotonin pathway modulator, in patients with pulmonary arterial hypertension (PAH). This analysis presents the results from the Latvian center, which enrolled 12 participants.

**Methods:** 12 participants were randomized into three groups: placebo (n = 2), rodatristat ethyl 300 mg twice daily (n = 6), and rodatristat ethyl 600 mg twice daily (n = 4). The majority (75%) were female. The trial consisted of a 24‐week main phase and an open‐label extension (OLE). Seven participants enrolled in the OLE, with five continuing on 300 mg and two on 600 mg of rodatristat ethyl. The primary outcome was the percent change in pulmonary vascular resistance (PVR) from baseline to week 24.

**Findings:** The median percent change in PVR from baseline to week 24 showed a reduction by 5.6% for the placebo group, increase of 86.4% for the rodatristat ethyl 300 mg group, and increase of 14.5% for the rodatristat ethyl 600 mg group. Five participants did not enroll in the OLE due to side effects or disease deterioration requiring treatment escalation. Among the seven participants in the OLE, six completed the 24‐week extension. Four of these seven participants experienced WHO Functional Class deterioration, with a median time to worsening of 47.22 weeks. The median percent change in PVR from baseline to OLE week 24 was 68%, with no significant change between week 24 of the main study and OLE week 24. Seven of 12 participants on rodatristat required transition to triple therapy, two after the main study and five after the OLE. Initiation of triple therapy reduced median PVR by 34%.

**Conclusions:** Although rodatristat has demonstrated negative effects on PAH patients, it is essential to pursue additional trials that focus on novel pathogenetic pathways, given that PAH is a progressive and currently incurable condition.

## 87. Role of Conventional Dendritic Cells Type 2 In Schistosoma‐Induced Pulmonary Hypertension

Fonseca Balladares D^1,2^, Kumar R^1,2^, Mickael C^3^, Nolan K^1,2^, Graham B^1,2^

^1^University of California San Francisco (UCSF), San Francisco, United States, ^2^Zuckerberg San Francisco General Hospital and Trauma Center, San Francisco, United States, ^3^University of Colorado, Aurora, United States

**Title:** Role of Conventional Dendritic Cells Type 2 in Schistosoma‐Induced Pulmonary Hypertension

**Authors:** Dara C. Fonseca Balladares, Rahul Kumar, Claudia Mickael, Kevin Nolan, Brian B. Graham

**Rationale:** Schistosomiasis is a parasitic infection and a major cause of pulmonary hypertension (PH), characterized by the thickening of lung blood vessels. Previous studies found adaptive immunity, particularly via Th2 CD4 T cells, is necessary for Schistosoma‐induced PH. This study investigates the role of dendritic cells (DCs), specifically conventional DCs type 2 (cDC2s) which present antigens to CD4 T cells, hypothesizing that cDC2s contribute to Th2‐driven Schistosoma‐induced PH.

**Methods:** Heterozygous CD301b‐DTR mice were sensitized with 240 S. mansoni eggs/gram via intraperitoneal (IP) injection, followed by an intravenous (IV) challenge of 175 eggs/gram 2 weeks later. Diphtheria toxin (DT, 0.5 µg/IP) or PBS was administered every three days starting the day before IV egg administration and continuing for 7 days. On day 7, the mice underwent right heart catheterization to measure right ventricular systolic pressure (RVSP) and the lungs were digested for flow cytometry to quantify DC depletion.

**Results:** DT treatment resulted in 80% depletion of cDC2s in CD301bBDTR mice. However, there was no protection against PH, as indicated by unchanged RVSP and right ventricular hypertrophy. No differences in peri‐egg granuloma volume or lung vessel remodeling was observed between the groups. IL‐4 levels decreased about 50%.

**Conclusions:** cDC2s appear to be dispensable in the Type 2 inflammation and Schistosoma‐induced PH that follows egg embolization to the lungs. The modest decrease in IL‐4 was not sufficient to confer protection. Other cells appear to be sufficient to activate Th2 cells. Further studies are needed to explore alternative immune mechanisms and antigen‐presenting cells.

## 88. Plasma Metabolomics in the Clinical Course of Patients With Pulmonary Hypertension

Harbaum L^1^, Dittrich A^1^, Matschl U^1^, Klose H^1^

^1^Universitätsklinikum Hamburg‐Eppendorf, Hamburg, Germany

**Background:** Pulmonary arterial hypertension (PAH) and inoperable or recurrent chronic thromboembolic pulmonary hypertension (CTEPH) are treated with drugs targeting nitric oxide, endothelin, prostacyclin, or activin pathways. However, individual responses to these therapies vary, and a better molecular understanding of short‐term treatment responses may highlight biological pathways involved.

**Objectives:** This longitudinal study aimed to identify metabolic pathways associated with changes in patient risk profiles through the analysis of plasma metabolites before and after medical treatment in patients with PAH and inoperable/recurrent CTEPH.

**Methods:** Untargeted metabolomics were performed on plasma samples from 44 patients with PAH (n = 35) or inoperable/recurrent CTEPH (n = 9) at two time points, before and after the initiation or escalation of medical therapy. The median interval between sampling was 7 months (interquartile range 4–12). Changes in metabolite levels were analysed in relation to risk profiles, as assessed using the four‐strata model outlined in the European guidelines for treatment of pulmonary hypertension.

**Results:** Partial least squares discriminant analysis revealed significant differentiation in metabolomic profiles before and after treatment initiation or escalation. Of the 745 metabolites analysed, 54 were significantly associated with improved or deteriorated risk profiles, as determined by receiver operating characteristic analyses (p < 0.05). Six metabolites, including four lipids and two amino acids, remained significant after multiple testing correction (area under the curve > 0.77, FDR q < 0.1). These metabolites were similarly predictive of risk profile changes following both treatment initiation and escalation. Pathway enrichment analysis highlighted the involvement of sphingomyelin, sphingolipid synthesis, and phosphatidylinositol pathways (p < 0.05).

**Conclusion:** Plasma metabolomic profiles are closely linked to treatment response in PAH and inoperable/recurrent CTEPH. Lipid metabolism shows promise as a biological marker of treatment response.

## 89. Early Phase Clinical Development of APL‐9796, A Monoclonal Antibody (AB) Targeting Zinc‐Regulated Transporter (ZRT)‐Like, Iron‐Regulated Transporter (IRT)‐Like Protein 12 (ZIP12), for The Treatment of Pulmonary Hypertension (PH)

Cardinal M^1^, Gurrell R^1^, Aggarwal S^1^, Wilkins M^2^, Zhao L^2^, Kiely D^3^, Wason J^4^, Church C^5^, Suntharalingam J^6^, McCabe C^7^, Toshner M^8^, Howard L^2^, Rothman A^3^

^1^Apollo Therapeutics, Cambridge, United Kingdom, ^2^Imperial College, London, United Kingdom, ^3^University of Sheffield, Sheffield, United Kingdom, ^4^Newcastle University, Newcastle, United Kingdom, ^5^Queen Elizabeth University Hospital, Glasgow, United Kingdom, ^6^University of Bath, Bath, United Kingdom, ^7^Royal Brompton Hospital, London, United Kingdom, ^8^University of Cambridge, Cambridge, United Kingdom

ZIP12 is aberrantly expressed in arterial smooth muscles cells in models of hypoxia and in patients with PH. APL‐9796, a high‐affinity monoclonal Ab binds to ZIP12, blocks its function and reduces pulmonary arterial pressure (PAP) and ventricular hypertrophy in rodent models of PH. These nonclinical findings supported the Phase 1 and planned Phase 2 clinical development of APL‐9796 in PH.

APL‐9796 was evaluated in a Phase 1 single ascending dose trial in healthy participants. Subcutaneous (SC) APL‐9796 at dose levels of 0.3, 1.0, 3.0, 6.0, and 12.0 mg/kg were safe and well tolerated. No SAEs, treatment‐related AEs ≥ Grade 3 or AEs that prevented dose escalation were reported. Exposures (Cmax and AUC) of APL‐9796 were dose‐proportional, linear and support monthly dosing. At the 1.0 mg/kg dose level, mean concentrations exceeded the modelled EC50 for change in mPAP following APL‐9796 treatment in the induced‐hypoxia rat model of PH.

APL‐9796 will be evaluated in a Phase 2, open‐label, multicentre, multiple ascending dose (SC Q4W dosing for 24 weeks) trial in WHO Group 1/3 PH patients previously implanted with the CardioMEMS™ PAP sensor. The starting dose of the trial is 1.0 mg/kg. The trial will employ a Bayesian Optimal Interval design to determine the safety of up to three APL‐9796 dose levels, with up to N = 12 participants at a dose level. Following the completion of treatment, participants will be monitored through a 12‐month washout period.

The primary endpoints of the trial are safety related; the secondary endpoints focus on efficacy, PK and PD. The effects of APL‐9796 on mPAP, total pulmonary resistance, cardiac output and stroke volume will be monitored via the CardioMEMS™ telemeter. Additional clinical endpoints include 6MWD, NT‐proBNP, QoL measures, clinical worsening, WHO functional class, ERS risk scores and cardiac MRI.

## 90. Cardiopulmonary Exercise Testing in Patients With Dyspnea After Pulmonary Embolism

Santos A^1,2^, Oliveira F^1,2^, Portella A^1^, Parcher S^1^, Santos J^1,2^, Menezes T^1,2^, Sperandio P^1^, Ota‐ Arakaki J^2^, Oliveira R^1,2^, Ferreira E^1,2^

^1^Exercise Physiology Unit (SEFICE), Respiratory Division, Unifesp/EPM, São Paulo, Brazil, ^2^Pulmonary Circulation Group, Respiratory Division, U, São Paulo, Brazil

Acute pulmonary thromboembolism (PE) is a severe condition with a risk of long‐term sequelae. Half of the cases persist with dyspnoea, making it essential to assess for chronic thromboembolic disease with or without pulmonary hypertension (CTEPH or CTED, respectively).

**Objectives:** The aim of the study was to investigate patients referred from the Pulmonary Circulation Clinic for the Exercise Physiology Laboratory with persistent Dyspnea after PE by using CPET as a tool for investigating exertional limitation.

**Methods:** A total of 99 patients, referred for investigation of dyspnoea 3‐6 months after acute PE, underwent incremental CPET between 2018 and 2023. All patients underwent chest CT angiography and echocardiography during the evaluation, and CTEPH was confirmed by RHC. The data was obtained from the cohort approved by CEP‐Unifesp.

**Results:** 67% were female, mean age 48±16years and BMI 30 ± 6 kg/m2. Most patients had NYHA II/III (65%). Patients were divided into CTEPH(n = 32), CTED(n = 28), and Dyspnea woCTEPH/CTED(n = 39) after a full investigation. CPET was compared among the 3 groups: VO2peak (64 ± 19, 81 ± 22 vs 86 ± 24%pred,p < 0,0001), peakWR (69 ± 26, 86 ± 38 vs 92 ± 37 W,p = 0.016), O2pulseAT (7 ± 2, 8 ± 3 vs 10 ± 4 ml/bat, p < 0.0001) and O2pulsepeak (8 ± 3, 10 ± 3 vs 11 ± 4 ml/bat,p < 0,0001 or 74 ± 19, 90 ± 21 vs 98 ± 27%pred, p < 0,0001), respectively. Regarding slopeVE/VCO2 and PETCO2 (rest, AT, and peak), CTEPH patients had higher and lower levels, respectively, compared to CTED and Dyspnea groups (p < 0.0001).33% of patients with Dyspnea had dysfunctional breathing or erratic ventilatory patterns compared to 17,9% in CTED and 21% in CTEPH (p > 0.05). Patients with persistent Dyspnea after PE had final diagnoses of asthma, COPD, obesity, AOS, dysautonomia, long COVID, and anxiety.

**Conclusions:** Long‐term outcomes after acute PE range from complete recovery to a diagnosis of CTEPH. The findings of this study underscore the potential of CPET as a valuable tool for patients with post‐PE dyspnoea to help differentiate between PH and non‐PH patients, thereby improving patient care and outcomes.

## 91. Inhaled Prostanoids in The Management of Pediatric Pulmonary Hypertension: A Scoping Review

Rogers H^1^, Stein M^1^, Mistry M^1^, Farias M^1^, MacLoughlin R^2^, Satler C^3^, Jenkins K^1^

^1^Boston Children's Hospital, Boston, USA, ^2^Aerogen, Galway, Ireland, ^3^Respira Therapeutics, Inc., Palo Alto, USA

**Introduction:** Pediatric pulmonary hypertension (PH) is a rare but serious condition characterized by elevated pulmonary artery pressure (PAP). The estimated incidence of pediatric PH is 4.8 to 8.1 per million children annually, with significant morbidity and mortality risks if untreated. Recent advancements in targeted therapies, especially inhaled prostacyclin analogs, have emerged as promising interventions.

**Methods:** This scoping review synthesizes the current literature on inhaled prostacyclin therapies for the management of pediatric PH. A comprehensive search strategy across multiple databases identified 21 studies investigating the use of inhaled prostanoids in pediatric patients (aged 0‐21). Data on study characteristics, participant demographics, treatment modalities, and hemodynamic outcomes were extracted and analyzed.

**Results:** A total of 598 pediatric participants across PH subtypes were included. Neonate (82; 30%), infant (62; 23%), child (89; 33%), and adolescent (37; 14%) age groups were represented, and most had associated PAH due to congenital heart disease (CHD) (375; 63%). Inhaled iloprost (556; 93%) and inhaled treprostinil (42; 7%) were utilized in the pediatric population. Across studies, 68% of patients were considered responsive to treatment. On average, mean pulmonary artery pressure (mPAP) decreased by 13.4 mmHg and pulmonary vascular resistance (PVR) decreased by 5.0 Indexed Woods Units. Jet nebulizer (436 participants; 73%), dosing by weight (355 participant; 59%), and a treatment duration of 1‐7 days (313 participants; 52%) were the most common treatment modalities.

**Discussion:** This review supports the use of inhaled prostanoids for the management of PH across the pediatric age spectrum. Inhaled iloprost treatments, in particular, showed positive hemodynamic impacts, on average. The high level of non‐responders could be due to variability in treatment protocols. While inhaled therapies have significant potential for the treatment of pediatric PH, further research is necessary to establish treatment protocols, improve safety and efficacy, and expand therapeutic options for this vulnerable population.

## 92. Preclinical Models Support the Synergistic Potential of Seralutinib and Sotatercept in Treating Pulmonary Arterial Hypertension (PAH)

Sitapara R^1^, Garcia E^1^, Osterhout R^1^, Ding Z^1^, Zisman L^1^, Duong‐Verlé T^1^, Roscigno R^1^, Aranda R^1^, Humbert M^2,3^, Tu L^2,3^, Guignabert C^2,3^, Bruey J^1^

^1^Gossamer Bio, Inc., San Diego, USA, ^2^Université Paris‐Saclay, Hypertension Pulmonaire: Physiopathology and Innovation Thérapeutique, HPPIT, Faculté de Médecine, Le Kremlin‐Bicêtre, France, ^3^INSERM UMR_S 999, HPPIT, Le Kremlin‐Bicêtre, France

**Background:** PAH is a progressive disease characterized by pathogenic remodeling of the pulmonary vasculature, driven by inflammation, proliferation, and fibrosis. Seralutinib, an investigational inhaled PDGFRα/ß, CSF1R, and c‐KIT kinase inhibitor, targets these pathways. Sotatercept, a new PAH therapy, targets TGF‐ß superfamily ligands to restore balance between pro‐ and anti‐proliferative pathways. While both compounds have distinct mechanisms, evidence for intersecting pathways suggests that combined inhibition of PDGF and TGF‐ß might offer complementary benefits. This study examined the combined efficacy of seralutinib and ACTRIIA‐Fc (sotatercept analog) in preclinical models.

**Methods:** The combination of seralutinib and ACTRIIA‐Fc was evaluated in the SuHx rat model of PAH. Proliferation was measured in normal and idiopathic (I) PAH patient‐derived pulmonary artery smooth muscle cells (hPASMCs). The release of fibrotic markers was assessed in human lung fibroblasts (HLF) and human cardiac fibroblasts (HCF).

**Results:** In the SuHx model, treatment with seralutinib and ACTRIIA‐Fc alone decreased mean pulmonary artery pressure (mPAP) by 23% and 28%, respectively. The combination produced a 78% reduction in mPAP at clinically relevant doses. Combined treatment also significantly reduced right ventricular (RV) systolic pressure, RV hypertrophy, pulmonary vascular resistance index, and muscularization of small pulmonary arteries compared to vehicle control and individual seralutinib or ACTRIIA‐Fc treatments. In normal and IPAH‐derived hPASMCs, seralutinib completely inhibited PDGFBB and human serum‐induced proliferation, while ACTRIIA‐Fc alone showed modest inhibition of proliferation at clinically relevant concentrations; combination did not show an additive anti‐proliferative effect. In HLFs and HCFs, combination treatment led to a greater‐than‐additive reduction in fibrotic markers fibronectin and pro‐collagen Iα1.

**Summary:** These results highlight the complementary mechanisms of action of seralutinib and sotatercept, suggesting the clinical potential of combination therapy. Preliminary combination experience is expected from the phase 2 open‐label extension (NCT04816604) and phase 3 PROSERA studies (NCT05934526), which allow for protocol‐specified use of sotatercept with seralutinib.

## 93. Macitentan and Phosphodiesterase‐5 Inhibitor As Monotherapy or in Combination in Newly‐Diagnosed Patients With Pulmonary Arterial Hypertension: A Pooled Analysis

McLaughlin V^1^, Sauvageot N^2^, Hennessy B^2^, Panjabi S^3^, Paoli C^3^, Linder J^2^, Bayer B^2^, Söderberg S^4^, Gaine S^5^, Lange T^6^, Kim N^7^

^1^Department of Cardiovascular Medicine, University of Michigan, Ann Arbor, USA, ^2^Actelion Pharmaceuticals Ltd, a Johnson & Johnson company, Allschwil, Switzerland, ^3^Actelion Pharmaceuticals US, Inc., a Johnson & Johnson company, Titusville, USA, ^4^Department of Public Health and Clinical Medicine, Cardiology and Heart Centre, Umeå University, Umeå, Sweden, ^5^Department of Respiratory Medicine and Pulmonary Hypertension, National Pulmonary Hypertension Unit, Mater Misericordiae University Hospital, Dublin, Ireland, ^6^Department of Pulmonology, Kreisklinik Bad Reichenhall & Faculty of Medicine, Regensburg University, Bad Reichenhall & Regensburg, Germany, ^7^Division of Pulmonary, Critical Care and Sleep Medicine, University of California, La Jolla, USA

**Introduction:** Initial combination therapy with an endothelin receptor antagonist (ERA) and a phosphodiesterase‐5 inhibitor (PDE5i) is guideline‐recommended as standard‐of‐care in pulmonary arterial hypertension (PAH) in patients with low or intermediate risk. Despite these recommendations, there is a lack of data on the effect of initial combination therapy on survival outcomes.

**Objective:** To compare time to all‐cause mortality between initial macitentan and PDE5i combination therapy versus as monotherapy.

**Methods:** This post‐hoc, retrospective analysis pooled long‐term patient‐level data (follow‐up time ≥ 1 year) from trials and observational registries where prospective all‐cause mortality data was available from PAH‐incident adults initiated with either macitentan 10 mg or PDE5i monotherapy, or their combination (macitentan+PDE5i). Propensity score (PS) methods were used to balance key demographic and disease characteristics in the monotherapy cohorts to match that of the combination cohort. Hazard ratios (HRs) were computed from a Cox regression model that included weights calculated from the PS. Weighted Kaplan‐Meier estimates and corresponding 95%CI were computed at 6‐monthly timepoints.

**Results:** In total, 2201 patients met inclusion criteria from four clinical studies1 and three registries2. Treatment cohorts were: macitentan+PDE5i (n = 754; 421 tadalafil/324 sildenafil/9 other), macitentan monotherapy (n = 654), and PDE5i monotherapy (n = 793; 301 tadalafil/490 sildenafil/2 other). After weighting, characteristics were broadly similar between cohorts. Initial macitentan+PDE5i was associated with a significant 39% risk reduction of all‐cause mortality versus PDE5i monotherapy (HR:0.61, 95%CI:0.46–0.82) and a 32% risk reduction versus macitentan monotherapy (HR = 0.68, 95% CI:0.48–0.95). Treatment effects were more pronounced when macitentan was combined with tadalafil (49–43%; HR:0.51, 95%CI:0.30–0.85 and HR:0.57, 95%CI:0.37–0.88, respectively) than sildenafil (31–26%; HR:0.69, 95%CI:0.45–1.04 and HR:0.74, 95%CI:0.46–1.17, respectively).

**Conclusions:** This pooled analysis of 2201 patients from clinical trials and observational registries may indicate a survival benefit of initial macitentan+PDE5i combination therapy compared with either monotherapy in patients newly diagnosed with PAH.

References:¹SERAPHIN:NCT00660179/GRIPHON:NCT01106014/TRITON:NCT02558231/REPAIR:NCT02310672;²REVEAL:NCT00370214/OPUS:NCT02126943/EXPOSURE:EUPAS19085.

## 94. Interim Results From PHINDER: Pulmonary Hypertension Detection in Patients With Interstitial Lung Disease for Earlier Detection

Kiely D^1^, DerSarkissian M^2^, Shlobin O^3^, Zisman D^4^, Beck E^5^, Kulkarni T^6^, Sahay S^7^, Chavarria M^7^, Broderick M^8^, Lee D^8^, Maher K^8^, Shen E^8^, Paxton K^9^, Scholand M^5^

^1^University of Sheffield, ^2^Analysis Group, ^3^Inova Fairfax Hospital, ^4^Cleveland Clinic Florida, ^5^University of Utah Health, ^6^The University of Alabama at Birmingham, ^7^Houston Methodist, ^8^United Therapeutics, ^9^University of North Carolina at Chapel Hill,

**Introduction:** Early detection of pulmonary hypertension (PH) in patients with interstitial lung disease (ILD) allows for timely intervention. There are no evidence‐based guidelines for PH screening in this population. The PHINDER (NCT05776225) study aims to identify clinical features associated with PH in patients with ILD to develop a risk tool or predictive algorithm.

**Methods:** PHINDER is a prospective, multicenter study enrolling approximately 200 ILD patients. Participants receive assessments (physical exam, PFT, HRCT, echocardiogram, etc.) followed by right heart catheterization (RHC). An interim analysis conducted July2024 used descriptive statistics to summarize baseline features. Correlations to PH‐ILD, defined as mPAP > 20 mmHg, PVR > 2 WU, and PAWP ≤ 15 mmHg, were assessed using point‐biserial correlation (r ≥ 0.7, strong correlation) and Cramer's V (V ≥ 0.3, strong correlation) for continuous and categorical variables, respectively.

**Results:** At the interim analysis, 58 patients were enrolled. 29 patients (50%) had RHC‐confirmed PH‐ILD. Participants were predominantly white (88%) males (57%) with a mean age of 71.3 ± 9.8 and a mean time since ILD diagnosis of 3.8 ± 3.8 years. Features strongly correlated to PH were enlarged pulmonary artery (Cramer's V = 0.47) and a right ventricle to left ventricle (RV/LV) ratio > 1 (Cramer's V = 0.44). Findings moderately correlated to PH included findings on physical exams, female sex, background antifibrotic therapy, use of supplemental oxygen, enlarged PAs in lung periphery, PA/aorta diameter ratio > 1, and ventricular septal flattening.

**Discussion/Conclusion:** Preliminary data from PHINDER suggest that PH is strongly correlated with HRCT findings of enlarged PA and RV/LV ratio > 1. It is worth noting that findings of RV dysfunction can be subtle even in severe PH‐ILD, with the only notable differences being TAPSE/sPAP and a lower SVI. Results from the completely enrolled PHINDER study will be used to develop a PH screening algorithm to aid in detection of PH in patients with ILD.

## 95. Chronic Thromboembolic Disease Causes Reduced Vascular Distensibility in Patients That Underwent Invasive Cardiopulmonary Exercise Testing

Deberaldini Marinho B^1^, Mitran R^2^, O. Al‐Qadi M^1^, Risbano M^1,2,3^

^1^Division of Pulmonary, Allergy, and Critical Care Medicine, Department of Medicine, University of Pittsburgh School of Medicine and UPMC, Pittsburgh, USA, ^2^Department of Internal Medicine, University of Pittsburgh School of Medicine and UPMC, Pittsburgh, USA, ^3^Center for Pulmonary Vascular Biology and Medicine, Pittsburgh Heart, Lung, Blood Vascular Medicine Institute, University of Pittsburgh School of Medicine and UPMC, Pittsburgh, USA

Patients with chronic thromboembolic disease (CTED) have persistent thrombi, despite anticoagulation, after an acute pulmonary embolism (PE) event and are at increased risk of developing complications like pulmonary hypertension. The pulmonary vascular distensibility coefficient (alpha) is a useful parameter in understanding pulmonary vascular disease. This study aims to investigate whether patients with CTED exhibit a decrease in pulmonary vascular distensibility in the absence of exercise pulmonary hypertension or overt pulmonary vascular disease.

70 patients underwent invasive cardiopulmonary exercise testing (iCPET) for evaluation of exercise intolerance. Cardiopulmonary and hemodynamic retrospective analysis was performed; subjects were split in four groups:

1‐CTED and normal peak VO₂ (n = 12).

2‐CTED and reduced peak VO₂ (n = 14).

3‐Patients with resolved PE (RPE), diagnosed with acute PE and with a normal V/Q scan in follow‐up (n = 16).

4‐Control group without history of PE with normal iCPET results (n = 28).

CTED patients with normal and reduced peak VO₂ had lower alpha (CTEDnVO₂: median= 0.86; IQR = 0.68 to 1.29) (CTEDrVO₂: median= 0.74; IQR = 0.32 to 1.39) compared to RPE (median= 1.28; IQR = 1.13 to 1.63) and controls (median= 1.39; IQR = 0.97 to 1.75).

In patients with RPE and CTED, alpha was inversely correlated with both pulmonary artery elastance (r = ‐0.57, p = 0.002) and slope of mPAP/CO (r = ‐0.58, p = 0.003).

At peak exercise, alpha exhibited a positive correlation with VD/VT (r = 0.52, p = 0.007) and an inverse correlation with total pulmonary resistance (TPR) (r = ‐0.44, p = 0.024) and pulmonary vascular resistance (PVR) (r = ‐0.53, p = 0.005). Notably, there were no correlations observed between alpha and stroke volume or cardiac output.

Patients with CTED have decreased pulmonary vascular distensibility compared to RPE and controls. This is likely indicative of chronic clot burden in CTED patients. Alpha appears to be a useful marker of vascular remodeling in patients with chronic thromboembolic disease without evidence of pulmonary hypertension.

## 96. Lung Transplant Waitlist Outcomes in Primary Pulmonary Hypertension Compared With Other Group B Diagnoses: A UNOS Analysis

Girgis R^1^, Manandhar Shrestha N, Loyaga‐Rendon R

^1^Corewell Health/Michigan State University College Of Human Medicine, Grand Rapids, United States

**Background:** Lung transplant is an important therapeutic option for advanced pulmonary vascular disease. Appropriate assessment of risk on the waitlist is important for the allocation of donor lungs. Prior studies of the Lung Allocation Score (LAS) utilized by the United Network for Organ Sharing (UNOS) have shown that both waitlist mortality and time to transplant are inferior for candidates with pulmonary vascular disease (PVD; Group B) relative to other diagnoses. The LAS does not distinguish between Primary Pulmonary Hypertension (PPH) and other types of PVD.

**Methods:** We sought to assess differences in waitlist outcomes between PPH and other Group B diagnoses. Adult candidates listed for lung transplant in the UNOS database between May 2005 and May 2021 were included. Time to death or transplant within one year of listing was compared between the two groups with a competing events analysis.

**Results:** A total of 1741 candidates were identified, 997 PPH and 744 other Gp B. The PPH group was younger (47 ± 14 vs. 53 ± 11 y) and had a lower proportion of males (26% vs. 38%). Hemodynamics were significantly worse in the PPH group with higher mean pulmonary artery pressure, lower cardiac index and higher pulmonary vascular resistance (10.4 ± 6 vs. 6.6 ± 5 WU; P < 0.001) and 6 MWD was lower (236 vs. 204 m), despite a similar LAS [38.8 (IQR: 34, 45) vs. 39.5 (35, 45)]. At one year after listing, the cumulative incidence function of death or removal from the list was 17% for PPH patients compared with 12% for other Gp B subjects (P = 0.004 by Gray's test) while that of transplant was 60% for PPH and 71% for other Gp B (P < 0.001).

**Conclusion:** Lung transplant candidates with PPH have waitlist outcomes inferior to other pulmonary vascular diseases. Allocation systems should appropriately classify pulmonary hypertension diagnoses to better gauge risk on the waitlist.

## 97. Iloprost Use in Children: Thirteen Years of Experience

Varghese N^1,2^, Spielberg D^1,2^, Bhatt N^2^, Ruiz F^1,2^, Villafranco N^1,2^, Morales‐Demori R^1,2^, Whalen E^2^, Saley T^1,2^, Coleman R^1,2^

^1^Baylor College of Medicine, Houston, USA, ^2^Texas Children's Hospital, Houston, USA

**Background:** Iloprost is a nebulized prostacyclin with rapid onset, short half‐life, and few systemic side effects. Reports of use in children with pulmonary hypertension (PH) are limited to cardiac surgical or PPHN case reports. Here we describe a single center experience of inpatient iloprost use for stabilization of the unstable PH patient across the pediatric population.

**Data:** By retrospective chart review, we identified 329 patients ≤ 18 years of age treated with iloprost from September 2011 – September 2024. Age range was 2 days ‐ 18 years (median 9.5 months) weight range was 0.65 kg – 94 kg (median 8.6 kg), and PH classification spanned all five WSPH groups. Among infants, iloprost was used in infants ranging 22 6/7 – 41 6/7 weeks estimated gestational age (median 35 2/7 weeks). Indication for therapy was often clinical worsening and hemodynamic instability precluding systemic prostacyclin therapy.

Iloprost was nebulized through an inline mesh nebulizer via advanced airway, a non‐invasive airway or a handheld ultrasonic nebulizer for the spontaneously breathing patient. Dosing was 0.25‐1 mcg/kg/dose for patients < 20 kg and 5 mcg/dose ‐ 20 mcg/dose for patients > 20 kg. Most (n = 320) received iloprost on a scheduled basis, typically every 3 hours but as frequently as every 1 hour, while nine received as‐needed dosing only. Duration of scheduled therapy was median of 7 days/41 doses (range 1 day/1 dose ‐ 2,792 doses/329 days). Most patients received only one course of scheduled treatment and 12% (41/329) required multiple courses of therapy. There were no serious adverse events.

**Conclusions:** Iloprost can be considered for acute stabilization in pediatric PH. Customized administration to meet respiratory needs and interfaces is important to ensure delivery. Iloprost is a safe therapeutic option for the broader pediatric PH population and can be used without adverse events across inpatient settings.

## 98. Treatment With Recombinant FGF10 Reverses Experimental COPD‐Associated PH

Hadzic S^1^, Loku E^1^, Wu C^1^, Gredic M^1^, Pak O^1^, Gierhardt M^2^, Ciancio del Giudice N^2^, Fagundez C^2^, Kojonazarov B^1^, Sommer N^1^, Schermuly R^1^, Seeger W^1^, El Agha E^1^, Bellusci S^1^, Weissmann N^1^

^1^Excellence Cluster Cardio‐Pulmonary Institute (CPI), Universities of Giessen and Marburg Lung Center (UGMLC), Member of the German Center for Lung Research (DZL), Institute for Lung Health (ILH), Justus Liebig University, Giessen, Germany, ^2^Max‐Planck Laboratory for Heart & Lung Research, Biomedicine Research Institute of Buenos Aires (IBioBA) ‐ CONICET ‐ Partner Institute of the Max Planck Society, Buenos Aires, Argentina

Alterations in the pulmonary vasculature accompany the development and progression of chronic obstructive pulmonary disease (COPD). The narrowing of arteriole diameter and the loss of small pulmonary vessels associated with severe emphysema contribute both to pulmonary hypertension (PH) severity in COPD. In experimental mouse models, boosting developmental pathways via fibroblast growth factor 10 (FGF10) reversed established PH and emphysema. However, the precise mechanisms of FGF10‐mediated lung repair and the relationship between alterations in pulmonary vascular and alveolar compartments during the reversion of emphysema and PH still remains elusive.

In this study, emphysema and PH in mice were induced by a single intratracheal application of elastase. Treatment started 4 weeks later after the disease has been fully established. Commercially available recombinant FGF10 was administered once per week, either intravenously or intratracheally, targeting primarily the pulmonary vasculature or alveolar compartment, respectively. The degree of PH and emphysema was assessed after 12 weeks of treatment.

Intravenous FGF10 treatment affected pulmonary vascular remodeling and ameliorated elastase‐induced PH, as determined by non‐invasive echocardiography, hemodynamic measurements, and histological analysis. However, it did not affect severe pulmonary emphysema, as concluded after in vivo lung function tests and histological examination. Intratracheal FGF10 treatment ameliorated elastase‐induced pulmonary emphysema and PH. Histological analysis confirmed the repair process occurring in the alveolar compartment of the intratracheally treated mouse lungs upon elastase injury.

Our data showcase distinct FGF10‐mediated reparative mechanisms depending on whether the pulmonary vasculature or alveolar compartment was primarily targeted. Intravenous treatment reversed pulmonary vascular remodeling without affecting elastase‐induced emphysema. The intratracheal FGF10 application induced repair of the distal respiratory surface and capillary network, affecting blood flow resistance and ameliorating elastase‐induced PH. Further studies are needed to address the distinct molecular and cellular mechanisms behind FGF10‐mediated repair in alveolar and vascular structures.

## 99. Identification of A Novel KCNA5 Variant Disrupting Kv1.5 Channel Function

Olschewski A^1^, Csaki R^2^, Foris V^3^, Eichstaedt C^4^, Doboly A^2^, Grünig E^4^, Meszaros F^2^, Nagaraj C^3^, Almássy J^2^, Halank M^6^, Hinterdorfer K^5^, Olsson K^7^, Olschewski H^3^, Olschewski A^1^, Enyedi P^2^

^1^Experimental Anaesthesiology, Department of Anaesthesiology and Intensive Care Medicine, Medical University of Graz, Graz, Austria, ^2^Department of Physiology, Semmelweis University, Budapest, Hungary, ^3^Division of Pulmonology, Department of Internal Medicine, Medical University of Graz, Graz, Austria, ^4^Center for Pulmonary Hypertension, Thoraxklinik Heidelberg gGmbH, University Hospital Heidelberg and Translational Lung Research Center Heidelberg (TLRC), German Center for Lung Research (DZL), Heidelberg, Germany, ^5^Laboratory for Molecular Diagnostics, Institute of Human Genetics, Heidelberg, Germany, ^6^University Hospital Carl Gustav Carus of the Technical University of Dresden, Medical Clinic and Polyclinic I, Dresden, Germany, ^7^Department of Respiratory Medicine and Infectious Diseases, Hannover Medical School, Hannover, Germany

Pulmonary arterial hypertension (PAH) is a life‐threatening condition characterized by increased pulmonary vascular resistance due to vasoconstriction and vascular remodeling. Potassium (K⁺) channels are critical in regulating the function of small pulmonary arteries, and dysregulation of these channels has been implicated in PAH pathogenesis. In our international PAH cohort (n = 546 patients), targeted genetic screening identified 11 previously uncharacterized variants of the KCNA5 gene, encoding for the Kv1.5 channel.

We investigated the functional characteristics of six of these KCNA5 variants. Employing in two model systems—Xenopus laevis oocytes and HEK 293 cells—we analyzed their electrophysiological properties through voltage‐clamp and patch‐clamp techniques. Among the six variants, only the missense variant c.1303 G > A (p.Gly435Arg) exhibited a marked 87 ± 1.3% reduction in whole‐cell current as compared to the wild‐type Kv1.5 channel. This variant, identified in a female patient with idiopathic PAH (73 yr, mPAP=53 mmHg), was absent in more than 60,000 control subjects from the GnomAD database (v.2.1.1), underscoring its potential pathogenic significance.

Further analysis revealed that the p.Gly435Arg variant displayed slower activation and inactivation kinetics, likely due to the repulsive effect of the substituted arginine on the voltage sensor, disrupting normal channel gating. To explore whether this variant exerts a dominant‐negative effect on channel function, we generated concatenated dimer constructs linking a native Kv1.5 α‐subunit and a variant. In these dimeric channels, current amplitude decreased by 53 ± 3%, indicating impaired channel function in a heterozygous setting.

This new functional evidence may allow for re‐classification of the Gly435 variant from a variant of uncertain significance (class III) to a likely pathogenic variant (class IV). This is the first study providing evidence that this KCNA5 gene variant significantly disrupts Kv1.5 function.

## 100. Positioning, Enzymatic Processing and Binding Partners of Versican in Vascular Lesions of Pulmonary Arterial Hypertension

Westöö C^1^, Mutgan A^2,3^, van der Have O^1,4^, Mead T^5,6,7^, Ahmed S^8,9,10^, Lampei E^1,4^, Koch C^5,11,12^, Norvik C^1^, Aspberg A^13^, Bech M^14^, Peruzzi N^1,14^, Brunnström H^15,16^, Kwapiszewska G^2,3,17^, Rådegran G^8,9^, Apte S^5^, Tran‐Lundmark K^1,4^

^1^Department of Experimental Medical Science, Lund University, Lund, Sweden and Wallenberg Centre for Molecular Medicine, Lund University, Lund, Sweden, ^2^Ludwig Boltzmann Institute for Lung Vascular Research, Graz, Austria, ^3^Medical University of Graz, Otto Loewi Research Center, Graz, Austria, ^4^Children´s Heart Center, Skåne University Hospital, Lund, Sweden, ^5^Department of Biomedical Engineering, Cleveland Clinic, Cleveland, United States, ^6^Department of Paediatrics, Case Western Reserve University School of Medicine, Cleveland, United States, ^7^University Hospitals Rainbow Babies and Children's Hospital, Cleveland, United States, ^8^Department of Clinical Sciences Lund, The Section for Cardiology, Lund University, Lund, Sweden, ^9^The Haemodynamic Lab, The Section for Heart Failure and Valvular Disease, VO Heart and Lund Medicine, Skåne University Hospital, Lund, Sweden, ^10^Department of Education and Research, Helsingborg Hospital, Helsingborg, Sweden, ^11^Department of Pathology, University of South Dakota, United States, ^12^Sanford Laboratories, Sanford Health, United States, ^13^Department of Clinical Sciences Lund, Lund University, Lund, Sweden, ^14^Medical Radiation Physics, Department of Clinical Sciences, Lund University, Lund, Sweden, ^15^Department of Clinical Sciences Lund, Division of Pathology, Lund University, Lund, Sweden, ^16^Department of Genetics and Pathology, Division of Laboratory Medicine, Region Skåne, Lund, Sweden, ^17^Institute of Lung Health, German Center for Lung Research (DZL), Giessen, Germany

**Rationale:** The extracellular matrix proteoglycan versican accumulates in remodeled vessels in pulmonary arterial hypertension (PAH). Five isoforms of versican (V0‐V4) are generated through alternative splicing, distinguished by differential arrangement of the chondroitin sulfate chain attachment regions GAGα and GAGβ. Hyaluronan and tenascin‐C, known to interact with the core protein of versican, are elevated in patients with PAH. We aimed to determine the presence of different isoforms and versican cleavage products in PAH, and to study tissue co‐localization of versican with tenascin‐C and hyaluronan, in PAH.

**Methods:** Paraffin‐embedded tissue from idiopathic PAH (n = 10) were imaged with synchrotron‐based phase‐contrast micro‐CT and subsequently sectioned and analyzed by histology, immunohistochemistry and in situ hybridization. Failed donor lungs (n = 3) were used as controls. ELISA was used to measure plasma levels of versican fragments in idiopathic PAH patients (n = 11) and healthy controls (n = 10) plasma.

**Results:** GAGα and GAGβ‐containing versican isoforms were identified in the tunica media and neointima of all patients while the versican G1‐ and G3 domains did not consistently co‐localize. Synchrotron‐based phase‐contrast micro‐CT enabled detailed virtual three‐dimensional histological examination of the vascular microanatomy. Hyaluronan was primarily found in the neointima of vascular lesions, co‐localizing with versican G3 and the versican neoepitope DPEAAE. Tenascin‐C was found in thin‐walled collateral vessels near PAH lesions. Idiopathic PAH patient plasma samples had significantly higher levels of versican G3 containing fragments compared with healthy controls.

**Conclusion:** The distribution and distinct localization of versican isoforms indicate its alternative exon splicing and post‐translational processing in idiopathic PAH, with proteolytic cleavage by members of the ADAMTS‐family suggested by the increased anti‐DPEAAE staining. Versican is further strengthened as a responder to intimal injury in the pulmonary circulation and suggest versican G3 as a promising PAH biomarker. The presence of tenascin‐C in pressure‐relieving collateral vessels warrants further investigation.

## 101. The Role of Duox1 in a Mouse Model of Cigarette Smoke‐Induced Pulmonary Hypertension and Emphysema

Loku E^1,2^, Sharma V^1,2^, Lakshmi V^1,2^, Yu‐Wu C^1,2^, Nuguri C^1,2^, Gredic M^1,2^, Kraut S^1,2^, Kojonazarov B^1,2^, Pak O^1,2^, Seeger W^1,2^, Sommer N^1,2^, Weissmann N^1,2^, Hadzic S^1,2^

^1^Excellence Cluster Cardio‐Pulmonary Institute (CPI), Universities of Giessen and Marburg Lung Center (UGMLC), Member of the German Center for Lung Research (DZL), Justus Liebig University, Giessen, Germany, ^2^Institute for Lung Health (ILH), Universities of Giessen and Marburg Lung Center (UGMLC), Member of the German Center for Lung Research (DZL), Justus Liebig, Giessen, Germany

**Rationale:** Chronic obstructive pulmonary disease (COPD) encompasses chronic bronchitis, emphysema, and often at least mild pulmonary hypertension (PH). Augmented oxidative/nitrosative stress plays an important role in the pathogenesis of COPD. NADPH oxidases are a major endogenous source of oxidative stress, primarily producing reactive oxygen species (ROS) and hydrogen peroxide (H₂O₂). DUOX1, a non‐phagocytic NADPH oxidase is a key producer of H₂O₂, and contributes to cell signaling and host defense. DUOX1 is significantly downregulated in COPD patients’ lungs compared to healthy donors. However, the precise role of DUOX1‐derived H₂O₂ stress and its signaling mechanisms in COPD‐related PH remains unclear.

**Methods:** To model the downregulation of DUOX1 expression seen in human COPD lungs, we utilized transgenic Duox1 knock‐out (Duox1‐/‐) mice. Wild‐type (Wt) and Duox1‐/‐ mice were exposed to cigarette smoke (CS) for 3 and 8 months to assess PH and emphysema development via non‐invasive echocardiography, hemodynamic measurements, lung function tests, histological analysis, and in vitro assays.

**Results:** Duox1‐/‐ mice developed emphysema and PH spontaneously, indicated by a decline in lung function and elevated right ventricular systolic pressure. Chronic CS exposure further exacerbated the PH severity. Echocardiographic analysis showed worsened functional and structural changes in the right ventricle of CS‐exposed Duox1‐/‐ mice compared to the Wt counterparts. Worsened PH phenotype in experimental Duox1‐/‐ mice was associated with increased ROS levels and altered inflammatory response. Furthermore, bronchoalveolar lavage fluid (BALF) from CS‐exposed Duox1‐/‐ mice increased the proliferation of primary mouse smooth muscle cells in vitro, which aligns with pronounced vessel wall remodeling observed in lung sections of experimental mice.

**Conclusions:** Our findings from the animal model indicate that DUOX1 downregulation results in the spontaneous development of PH and emphysema. DUOX1 deficiency and CS exposure worsen the disease. Future research will explore the mechanisms that emerge from the interaction between DUOX1 depletion and CS exposure.

## 102. Pathogenic PAH Gene Variants Linked to Pulmonary Vascular Pruning in Non‐Hispanic White COPDGene Participants

Foris V^1,2^, Kim K^1^, Martini L^1^, Tern C^1^, Estepar R^3^, Washko G^4^, Wade R^5^, Wells J^5^, Silverman E^1,4^, Cho M^1,4^, Boueiz A^1,4^

^1^Channing Division of Network Medicine, Department of Medicine, Brigham and Women's Hospital, Harvard Medical School, Boston, USA, ^2^Division of Pulmonology, Department of Internal Medicine, Medical University of Graz, Graz, Austria, ^3^Surgical Planning Laboratory, Laboratory of Mathematics in Imaging, Department of Radiology, Brigham and Women's Hospital, Harvard Medical School, Boston, USA, ^4^Division of Pulmonary and Critical Care Medicine, Department of Medicine, Brigham and Women's Hospital, Boston, USA, ^5^Division of Pulmonary, Allergy, and Critical Care Medicine, University of Alabama at Birmingham, Birmingham, USA

**Introduction:** Understanding the genetic basis of PH‐COPD and its phenotypes could improve disease classification and inform treatment strategies. In this study, we examined the association between predicted pathogenic variants in known pulmonary arterial hypertension (PAH)‐associated genes and pulmonary vascular phenotypes in a large cohort of smokers.

**Methods:** We analyzed whole genome sequencing and clinical data from COPDGene. We used WGS Annotator to integrate outputs from ANNOVAR, SnpEff, VEP, and ClinVar annotation tools, and identified missense variants in 26 PAH‐associated genes with putative evidence for PAH causality. We further evaluated variant deleteriousness using SIFT, LRT, MutationTaster and MetaRNN prediction algorithms, along with additional thresholds for CADD, REVEL, and minor allele frequency. We tested the association of carriers of predicted deleterious variants with pulmonary artery to aorta ratio, pulmonary blood vessel volume for vessels with a cross‐sectional area ≤ 5 mm² (bv5), total pulmonary blood vessel volume (tbv), and the bv5/tbv ratio adjusted for age, sex, and principal components of genetic ancestry as predictors.

**Results:** We analyzed 10,550 whole genomes and 133,060 intronic and exonic variants in PAH‐associated genes. Carriers of pathogenic variants (36 subjects, 0.3% of the studied COPDGene population) showed no significant differences in demographics, lung function, or pulmonary vascular imaging phenotypes compared to carriers of variants of uncertain significance (5,279 subjects) or benign variants (4,845 subjects) in both univariable and multivariable models. However, subgroup analyses based on evidence level for gene pathogenicity revealed that carrying predicted deleterious variants in PAH genes with definitive pathogenicity was associated with pulmonary vascular pruning in non‐Hispanic white subjects (43 cases, 6842 controls, p‐value = 0.04).

**Conclusion:** Pathogenic variants in known PAH‐associated genes are rare in the COPDGene cohort, but carriers of these variants may be associated with pulmonary vascular pruning. Further research is needed to determine the importance of genetic screening in identifying individuals at risk.

## 103. ATMOS, a Proof‐of‐Concept Trial of Inhaled Mosliciguat in Untreated PAH Or CTEPH Measuring Change in PVR

Ghofrani H^2,3,4,5^, Leuchte H^6,7^, Al‐Hiti H^8^, Halank M^9^, Jansa P^10^, Lange T^11,12^, Olschewski H^13^, Tello K^2,14^, Becker‐Pelster E^15^, Ligges S^15^, Nagelschmitz J^15^, Nikkho S^15^, Penugonda S^1^, Saleh S^15^, Vesterinen P^16^, Woischnik M^15^, Jung D^15^, Kopec G^17^

^1^Pulmovant, Inc, Waltham, USA, ^2^Department of Internal Medicine, Justus‐Liebig University Giessen and Marburg Lung Center, Giessen, Germany, ^3^Institute of Lung Health, Cardio‐Pulmonary Institute, Giessen, Germany, ^4^German Center for Lung Research (DZL), Giessen, Germany, ^5^Department of Medicine, Imperial College, London, UK, ^6^Neuwittelsbach Hospital, Academic Hospital of the Ludwig Maximilians University in Munich, Munich, Germany, ^7^German Center for Lung Research (DZL), Munich, Germany, ^8^Institute for Clinical and Experimental Medicine, Prague, Czech Republic, ^9^Division of Pulmonology, Medical Department I, University of Carl Gustav Carus of Technical University Dresden, Dresden, Germany, ^10^2nd Department of Medicine, General University Hospital, Prague, Czech Republic, ^11^Faculty of Medicine, Department of Internal Medicine II, University Hospital of Regensburg, Regensburg, Germany, ^12^Pulmonology and Intensive Care, Department of Internal Medicine, Kreisklinik Bad Reichenhall, Bad Reichenhall, Germany, ^13^Medical University of Graz, Graz, Austria, ^14^Department of Respiratory Medicine, Nordwest Krankenhaus, Frankfurt, Germany, ^15^Bayer AG, Germany, ^16^Bayer Pharmaceuticals, Finland, ^17^Pulmonary Circulation Centre, Department of Cardiac and Vascular Diseases, Jagiellonian University Medical College and John Paul II Hospital, Krakow, Poland

**Background:** Mosliciguat is a first‐in‐class, small‐molecule soluble guanylate cyclase (sGC) activator designed for lung‐targeted delivery via dry powder inhalation.

Under physiologic conditions, nitric oxide (NO) binds the prosthetic heme group of sGC, catalyzing cGMP production and leading to increased vasodilation, reduced inflammation and apoptosis, reverse vascular remodeling, and anti‐fibrotic effects. Oxidative stress seen in PH causes oxidation of sGC and formation of heme‐free sGC (apo‐sGC), abrogating NO‐induced activation of sGC. Mosliciguat has a novel ‘activator’ mechanism, directly binding to the heme‐binding pocket in sGC and mimicking the NO‐bound heme moiety resulting in cGMP production. By targeting NO‐insensitive apo‐sGC, it may provide effective vasodilation under conditions of NO depletion, including in patients with increased oxidative stress seen with PH.

**Objectives:** Assess peak percent change (PPC) from baseline in pulmonary vascular resistance (PVR, primary), safety, pharmacodynamics (PD), and pharmacokinetics.

**Methods:** Open‐label, single‐dose escalation (5 doses up to 4 mg) multicenter trial in patients (pts) with untreated PAH or CTEPH. Per Protocol Set (PPS) consisted of the NO‐nonresponsive pts with PVR ≥ 5 Wood units at baseline (N = 20, 4/dose).

**Results:** 38 pts received mosliciguat. For PPS, mean PPC of PVR from baseline [95%CI] was ‐21.0%[‐31.6%,‐10.4%], ‐16.1%[‐32.8%,0.7%], ‐25.9%[‐60.3%,8.4%], ‐38.1%[‐55.9%,‐20.3%] and ‐36.3%[‐48.3%,‐24.4%] for the 5 increasing doses, respectively. Reduced PVR persisted over the 3‐hour catheterization period at the highest doses. Pulmonary artery pressure decreased and at the highest dose mean cardiac output increased by ~25% from baseline. Levels of cGMP remained elevated through 24‐hours of observation. Effects were similar in NO‐responsive pts (PD Set N = 37). Most AEs were mild, with headache (n = 3;7.9%), decreased oxygen saturation and fatigue (n = 2;5.3% each) reported most frequently (N = 38). No safety relevant changes were seen in pulse rate, systolic blood pressure, or systemic vascular resistance.

**Conclusion:** Mosliciguat had favorable tolerability and resulted in a sustained, substantial reduction in PVR after a single inhaled dose.

## 104. CT‐Based Discrimination of CTEPH Using Deep Learning Clot Vessel Radiomics and Machine Learning

Nardelli P^1^, Cuttica M^3^, Mylvaganam R^3^, Rahaghi F^2^, San José Estépar R^1^

^1^Department of Radiology, Brigham and Women's Hospital, Harvard Medical School, Boston, USA, ^2^Division of Pulmonary and Critical Care, Brigham and Women's Hospital, Harvard Medical School, Boston, USA, ^3^Division of Pulmonary and Critical Care, Northwestern University Feinberg School of Medicine, Chicago, USA

**Introduction:** Chronic thromboembolic pulmonary hypertension (CTEPH) is a challenging condition to diagnose, requiring specialized imaging and interpretation. This study aimed to develop a CT‐derived machine learning‐based approach to discriminate CTEPH patients from controls and acute PEs.

**Methods:** We developed a machine learning model using radiomic features derived from intraparenchymal vessels in CT scans. Key features included histogram moments (mean, standard deviation, skewness, and kurtosis) of normalized vessel density, estimated clot area, and vessel radius at locations likely containing clots. Intraparenchymal vessels were extracted using a scale‐space particle approach. Clot detection and area quantification were conducted using a deep learning model trained on synthetic vessel cross‐sections. Five million synthetic vessel images with varying degree of central and wall occlusions were created as 32×32 pixel patches, simulating real vessels with clots. The model predicted the clot probability and area, while a separate neural network estimated vessel radius. Vessel density was normalized by the mean pulmonary artery density. An XGBoost classifier was trained on radiomic features from CTEPH and control cases, focusing on vessels with cross‐sectional areas between 5 and 30 mm² and clot probability ≥ 0.8. Model evaluation was performed using five‐fold cross‐validation, assessing AUC, sensitivity, specificity, and accuracy.

**Results:** CT scans from 176 healthy subjects and 87 CTEPH patients were analyzed. A stratified 30% test set maintained balanced disease prevalence. The XGBoost model achieved a mean AUC of 0.76 during cross‐validation and 0.74 on the test set, with sensitivity and specificity of 0.74 and 0.72, respectively. Validation of acute PE from CTEPH discrimination using 143 acute PE subject without CTEPH achieved an AUC of 0.92, with sensitivity and specificity of 0.80 and 0.95, respectively.

**Conclusions:** Our CT‐based machine‐learning model shows promise in automatically discriminating CTEPH from other conditions using vessel radiomics, with potential to improve diagnosis and management of CTEPH patients.

## 105. Sexual Dimporhism of Dexamethasone Protection From Hypoxic Pulmonary Hypertension

Nolan K^1^, Fonseca Balladares D^1^, Lee M^1^, Pasha Q^2^, Graham B^1^, Kumar R^1^

^1^Department of Medicine, University of California San Francisco, San Francisco, USA, ^2^Institute of Hypoxia Research, New Delhi, India

**Background:** Hypoxia‐induced pulmonary hypertension (PH) is a prevalent cardiovascular complication resulting from high‐altitude exposure and pulmonary disease‐associated hypoxia. The pathogenesis of hypoxic PH is primarily driven by inflammation and vasoconstriction. Recruitment of interstitial macrophages (IMs) leading to thrombospondin (TSP‐1) expression and activation of TGF‐β1 and Rho‐activated kinase (ROCK) contributes to hypoxic‐PH. Clinically, dexamethasone (DEX), a corticosteroid with anti‐inflammatory properties, is used to treat acute hypoxic pathology, although its precise mechanisms of action remain unclear. Females may be more susceptible to hypoxic‐PH, but also have greater clinical benefit from DEX prophylaxis. We hypothesized that DEX exerts its protective effects by inhibiting inflammation and ROCK activity, particularly in females, thereby preventing the development of hypoxic‐PH.

**Methods and Results:** Mice were exposed to normobaric 10% FiO2 hypoxia. DEX treatment reduced CCL2 production in the lungs measured by ELISA, leading to decreased recruitment of inflammatory CCR2+ monocytes quantified using flow cytometry. Notably, DEX was more effective in protecting females than males from hypoxia‐induced PH by lowering right ventricular systolic pressure (RVSP), as measured by right heart catheterization. Interestingly, acute intravenous administration of fasudil, a potent ROCK inhibitor, significantly reduced RVSP in hypoxia‐exposed wildtype mice, with a more pronounced effect in females than males. In DEX‐treated female mice, fasudil further lowered RVSP, while no such effect was observed in males, suggesting that DEX treatment significantly but not completely suppresses ROCK activation.

**Conclusions:** DEX blocks the recruitment of inflammatory IMs and decreases Rho‐kinase signaling and vasoconstriction induced by hypoxia with a greater effect in females than males.

**Funding Sources:** ATS, United Therapeutics, AHA, and NIH

*Correspondence: rahul.kumar2@ucsf.edu

## 106. Women With PAH: Alignment in Patient and Physician Perspectives on Quality of Care

Vaidya A^1^, McLaughlin V^2^, Thakur T^3^, Watzker A^3^, O'Hara M^4^, Yeh M^4^, Athavale A^4^, Delcroix M^5^, Lautsch D^3^, Preston I^6^

^1^Temple University, Philadelphia, United States, ^2^University of Michigan Ann‐Arbor, Ann Arbor, United States, ^3^Merck and Co., Inc, Rahway, United States, ^4^Trinity Life Sciences, Waltam, United States, ^5^University Hospitals of Leuven, Leuven, Belgium, ^6^Tufts School of Medicine, Boston, United States

**Objectives:** ESC/ERS guidelines recognize the need for specialized care for women with pulmonary arterial hypertension (PAH). The study aimed to understand perspectives of physicians and patients regarding delivery of care to women with PAH.

**Methods:** This is a multi‐country, qualitative interview study with independent physician (cardiologist and pulmonologist) and patient (women aged 21‐50 with PAH) cohorts. Respondents participated in interviews or a focus group with structured open‐ended questions on perceived burden and management of PAH, focusing on family planning, pregnancy, and childcare. Data were analyzed qualitatively for alignment (+) and lack thereof (‐) in patients and physician perceptions. This study was deemed exempt/approved in all countries, as applicable.

**Results:** Fifteen (15) physicians (United States [US] = 8, United Kingdom [UK] = 4, Canada=3; 7 Cardiologist, 8 Pulmonologist) and 24 women with PAH (Mean age=40.7, US = 10, Canada=3, UK = 3, Germany=4, Italy=4) were interviewed. Themes highlighted by both physicians and patients included:

• Misdiagnosis/delayed diagnosis (+): Physicians (8/15) and patients (9/24) reported delayed diagnosis or misdiagnosis due to early symptoms being attributed to other conditions.

• Patient support (+/‐): Resources for disease education and symptom management were deemed inadequate by physicians (8/15) and patients (12/24). While physicians reported awareness of sensitive topics such as family planning and survival, 11/24 patients perceived insensitivity in how physicians communicated.

• Clinical decision‐making (‐): All physicians advised against pregnancy, which aligns with treatment guidelines. However, women with PAH (10/24) expressed the need for greater patient involvement in decision‐making regarding family planning.

• Economic burden (+): Most physicians and patients reported high overall financial burden related to treatment [expensive medications (8/15 physicians; 8/24 patients) and high out‐of‐pocket costs (5/15 physicians; 16/24 patients).

**Conclusion:** Treatment guidelines and policy considerations should focus on providing improved diagnosis, patient support, family planning, and financial considerations for women with PAH to facilitate earlier diagnosis and optimal treatment.

## 107. Pediatric Pulmonary Hypertension Registry: Experience From Low Middle Income Country

Kulkarni S^1^, Wadile S^2^, Banpurkar A^2^

^1^Fortis Hospital Mumbai India, Mumbai, India, ^2^Sathya Sai Sanjeevani Hospital, Mumbai, India

There are very few pediatric Pulmonary Hypertension (PAH) registries with a scarcity of data from developing countries. Patients between 2 months to 18 years of age diagnosed with PAH were included in our study. Patients with transient PAH like hyperkinetic PH associated with congenital heart defects (CHD) were excluded. All patients were classified into Idiopathic PAH (IPAH) and Associated PAH (APAH) based upon their diagnosis. Our registry included 100 pediatric patients from 2019 to January 2024 with a nearly equal sex ratio (M: F = 1:1). Easy fatigability, syncope followed by features of right heart failure were most common presenting features. 15 patients had IPAH and the rest 85 belonged to APAH. In APAH group, 75 patients had CHD with either right to left shunt (Eisenmenger syndrome) or elevated pulmonary vascular resistance (PVR) prohibiting shunt closure, 6 had postoperative PAH, 2 with left heart disease, one secondary to portosystemic shunt. All patients (100%) in the IPAH group and 42 patients (56%) in APAH were on pulmonary vasodilators. The median follow‐up duration was 11 months (4‐56months). There were 3 deaths in IPAH (20%) and 2 in APAH (2.3%) groups. The mean survival time for patients with IPAH was 602.6 ± 90.8 days (95% C.I. 424.4 – 780.7) and it was significantly different when compared with both ES and post‐operative groups (1639.11 ± 47.1 days, 95% C.I. 1546.8 – 1731.3) (Log rank test ‐ p value ‐ 0.006). Children with shunt lesions had better functional class and no patient in this group died. As compared with registries from Western literature, our registry has more of Eisenmengers's population which reflects the regional practices especially late referrals for large shunts. The study highlights importance of careful evaluation in borderline operable patients with large shunts before subjecting them for surgery.

## 108. Prolidase Deficiency Causing Portal Hypertension Leading to Pulmonary Hypertension

Champion C^1^, Dryzer S^1^, Chartan C^1^

^1^ The University Of Texas At Austin ‐ Dell Medical School. Department of Pediatrics, Austin, United States of America

**Background:** Prolidase deficiency is a rare autosomal recessive disorder due to a biallelic pathogenic variant in the PEPD gene that causes a decrease in protein metabolism, collagen turnover, and matric remodeling particularly in connective tissue causing delayed wound healing, increased inflammation, and decreased cell proliferation. Clinical characteristics include severe skin lesions and telangiectasias, recurrent infections, organomegaly, chronic pulmonary disease, anemia and or thrombocytopenia.

**Case Report:** 13‐year‐old male presented with fifth digit osteomyelitis requiring surgical removal of his toenail. Patient with past history of renal tubular acidosis and severe transfusion dependent hemolytic anemia, both self‐resolved in infancy.

Patient ultimately diagnosed with prolidase deficiency due to pathognomonic skin ulcerations leading to several co‐morbidities: cirrhosis with esophageal varices and autoimmune hepatitis, thrombocytopenia, multiple skin lesions, progressive interstitial lung disease, and chronic systemic inflammation with inflammatory digital clubbing.

In 2022, patient diagnosed with portosystemic shunts with portal hypertension, but given normal echocardiogram, no other testing or diagnostics were performed.

Patient became hypoxemic after anesthesia from toenail removal, also with decreased exercise tolerance for the preceding months per additional report. Echocardiogram revealed right ventricular systolic pressure of 52 mmHg + right atrial pressure with systolic septal flattening and mild right ventricular dysfunction. Child also with positive bubble study.

After initiation of PH therapy, his functional status improved along with reduction in RVSP from 52 mmHg to 33 mmHg. Child also started on empiric CPAP for OSA and is being worked up for liver transplantation.

**Discussion:** Prolidase deficiency is a rare genetic disorder that has been linked to pulmonary and hepatic comorbidities. Early and routine screening for portosystemic shunts, portal hypertension and pulmonary hypertension should be standardized care for this population with goal to improve functional quality of life with PH targeted medications due to likelihood that liver and or lung transplantation is an unlikely option.

## 109. Unraveling Cellular Heterogeneity in the Lung: A Comprehensive Single‐Cell Transcriptomic Analysis

Kacar S^1^, James J^1^, Dekan A^1^, Rafikova O^1^, Rafikov R^1^

^1^Indiana University, Department of Medicine, Indianapolis, United States

Understanding cellular heterogeneity in the lung is crucial for elucidating pathobiology and developing targeted therapies for pulmonary vascular diseases. Recent advances in single‐cell transcriptomics (scRNAseq) have revealed that pulmonary capillary cells comprise two major phenotypes: aerocytes (aCaps) and general capillary cells (gCaps). To further explore cellular diversity, we employed two complementary techniques: (1) endothelial cell isolation from the lung followed by endothelial‐focused scRNAseq using 10x Genomics, and (2) whole‐lung scRNAseq at 100,000‐cell resolution using Parse Biosciences.

Our data demonstrate consistent identification of endothelial phenotypes across both techniques. We identified and characterized five novel subtypes of gCaps, each exhibiting distinct sub‐specialization based on gene expression profiles and functional analysis. Enriched endothelial cell analysis revealed that two gCap populations, expressing Scn7a+ and Clic4+ ion transporters, form the arterial‐to‐vein zonation and establish the primary capillary beds. We also identified mitotically active "root" cells (Flot1 + ) at the arterial‐venous zonation interface, potentially responsible for the regeneration and repair of adjacent endothelial subpopulations. The transition from gCaps to veins involves a venous‐capillary endothelium expressing Lingo2 + . Additionally, we discovered a distinct gCap population characterized by high Fabp4+ expression, metabolic activity, and tip‐cell markers, suggesting a role in angiogenesis regulation. We further studied Fabp4+ cells in a vessel‐on‐chip model and found that they support angiogenesis.

Whole lung scRNAseq using Parse Biosciences further resolved novel endothelial populations and revealed transitional states in other cell types, including T cells, stromal cells, and epithelial cells. This comprehensive approach offers a more complete view of cellular dynamics within the lung microenvironment. The identification and characterization of these novel subpopulations and transitional states significantly advance our understanding of cellular phenotypes, their interactions, and potential roles in lung disease pathogenesis. This knowledge may pave the way for more targeted therapeutic approaches in pulmonary medicine.

## 110. Unravelling Molecular Mechanisms of Nitrosative/Oxidative Stress‐Mediated Pulmonary Vascular Alterations in COPD

Wu C^1,2^, Doswada S^1,2^, Gredić M^1,2^, Čilić A^1,2^, Loku E^1,2^, Lakshmi V^1,2^, Sharma V^1,2^, Kleinhenn V^1,2^, Schaeffer J^1,2^, Wujak M^3^, Brandes R^4^, Seeger W^1,2^, Grimminger F^1,2^, Ghofrani H^1,2^, Schermuly R^1,2^, Kraut S^1,2^, Bellusci S^1,2^, Sommer N^1,2^, Weissmann N^1,2^, Hadzic S^1,2^

^1^Excellence Cluster Cardio‐Pulmonary Institute (CPI), Universities of Giessen and Marburg Lung Center (UGMLC), Member of the German Center for Lung Research (DZL), Justus Liebig University (JLU), Giessen, Germany, ^2^Institute for Lung Health (ILH), Universities of Giessen and Marburg Lung Center (UGMLC), Member of the German Center for Lung Research (DZL), Justus Liebig University (JLU), Giessen, Germany, ^3^Department of Medicinal Chemistry, Collegium Medicum in Bydgoszcz, Faculty of Pharmacy, Nicolaus Copernicus University in Toruń, Bydgoszcz, Poland, ^4^Institute for Cardiovascular Physiology, Goethe University, Frankfurt, Germany

Long‐term exposure to environmental hazards from cigarette smoke (CS) or air pollution can cause the development of chronic lung diseases, such as chronic obstructive pulmonary disease (COPD). Alterations in pulmonary vasculature, such as thickening of the intimal and medial vessel walls, often occur in COPD patients. One underlying pathomechanism is nitrosative/oxidative stress due to increased expression of inducible nitric oxide synthase (iNOS) and NADPH oxidase organiser 1 (NOXO1). Our preliminary results showed that CS induced iNOS and NOXO1 upregulation in pulmonary artery smooth muscle cells (PASMCs) and endothelial cells (ECs). However, the contributions of iNOS‐ and NOXO1‐expressing PASMCs and ECs to CS‐induced pulmonary vascular remodelling remain poorly investigated.

Against this background, we exposed transgenic mice to CS after deletion of iNOS or NOXO1 in Acta2+ or Tie2+ cells. Bronchoalveolar lavage fluid (BALF) was collected for Bio‐Plex Multiplex immunoassays to profile the cytokine landscape. Mice with iNos‐/Noxo1‐ablation in Acta2+ cells developed CS‐induced pulmonary hypertension (PH), whereas deletion of iNOS/NOXO1 in Tie2+ cells protected mice from PH. Interestingly, nitrosative/oxidative stress originating in Acta2+ cells was responsible for CS‐induced emphysema development. BALF from mice with iNos‐/Noxo1‐ablation in Tie2+ cells exerted a prominent proliferative effect on PASMCs, but only a tendency to increase chemotactic activities in monocytes. BALF profiling revealed that tumor necrosis factor alpha (TNF‐α)‐related cytokines were markedly decreased in Tie2+ cell‐specific iNos‐/Noxo1‐knockout mice, compared with wildtype mice and Acta2+ cell‐specific iNos/Noxo1‐knockout mice, in CS‐exposed condition. These results were further validated in vitro and using an ex vivo model of precision‐cut lung slices (PCLS).

Our study demonstrated that different phenotypes were developed in CS‐exposed mice depending on the cell‐specific ablation of iNOS/NOXO1. TNF‐α‐related cytokines were involved in PASMC proliferation associated with CS‐induced PH phenotype. Further investigations will elucidate the mechanisms underlying Acta2+ cell‐specific iNOS/NOXO1 contributions to emphysema development.

**Funding:** German Research Foundation‐268555672/SFB 1213/A07

## 111. Novel Potential Connection Between Lung Microbiome Dysbiosis and P2X7R/C‐IAP2‐Regulated Endothelial Survival in Schistosomiasis‐Associated Pulmonary Hypertension

Marinho Y^1^, Villarreal E^1^, Aboagye S^2^, Jun S^3^, Williams D^2^, Oliveira S^1^

^1^University of Illinois Chicago; College of Medicine; Vascular Immunobiology Lab; Dept. of Anesthesiology; Dept. of Physiology & Biophysics, Chicago, USA, ^2^University of Illinois Chicago; College of Medicine; Vascular Immunobiology Lab; Dept. of Medicine; Dept. of Microbiology and Immunology, Chicago, USA, ^3^Rush University; Department of Microbial Pathogens and Immunity, Chicago, USA

**Introduction:** Schistosomiasis‐associated Pulmonary Hypertension (Sch‐PH) is the most common form of group I PH worldwide. Recently, our data revealed that the preclinical Sch‐PH animal model exhibited gut/lung microbiome dysbiosis, microvascular apoptosis, and reduced Caveolin‐1 expression in lung endothelial cells (ECs). Although EC‐Caveolin‐1 function has been studied in PH, the role of endogenous anti/pro‐apoptosis sensors such as the inducible inhibitor of apoptosis protein 2 (c‐IAP2) and purinergic receptor P2X7 (P2X7R) remains widely unclear. Hypothesis: Lung microbiome dysbiosis contributes to lung EC apoptosis via c‐IAP2 suppression and P2X7R overactivation, leading to Sch‐PH.

**Material/Methods:** C57BL/6 J, Cdh5cre‐ERT2;cIAP1‐/‐;cIAP2fl/fl and End‐Scl.creERT2;Rosamt/mg mice exposed or not to S. mansoni eggs were used to quantify: right ventricular systolic pressure and hypertrophy (RVSP/RVH); protein expression by western blotting and immunohistochemistry; microbial metabolites by MassSpec; and Shotgun Metagenomics. Additionally, P2X7R function was inhibited by Brilliant Blue G (BBG; IP) prior to egg injections. Finally, human lung microvascular ECs (HMVECs‐L) and pulmonary artery ECs (HPAECs) +/‐ TNF‐α, INF‐γ, ATP, and A740003 (P2X7R inhibitor) for protein expression and apoptosis assay. Results/Discussion: The Sch‐PH model displayed lung inflammation and microbiome‐linked functional metabolic changes that can increase extracellular ATP levels, potentially over‐activating P2X7R. Indeed, data revealed higher P2X7R expression in the whole lung of IP/IV‐Egg exposed and EC‐c‐IAP2fl/fl mice compared to controls. Microvascular‐associated molecular differences were also observed at the functional level as c‐IAP2 overexpression and P2X7R inhibition selectively reduced apoptosis only in pre‐conditioned HMVEC‐L. Similarly, data indicated that P2X7R inhibition prevented the rise of RVSP in IP/IV‐Egg mice, whereas the specific novel EC‐c‐IAP2fl/fl mice showed a spontaneous increase in microvessel thickness/area. This effect could be due to inflammation and increased lung P2X7R expression in the pulmonary circulation of these animals.

**Conclusion:** Our data indicates that host‐microbiome dysbiosis may contribute to exacerbated lung microvascular P2X7R function and suppressed c‐IAP2 expression, leading to severe Sch‐PH.

## 112. Comprehensive Treatment Strategy for Pulmonary Arterial Hypertension Crisis in Gravida With Structural Heart Disease

Zhou C^1^, LUO H^1,2^

^1^ICU of Peking University of Shenzhen Hospital, Shenzhen, China, ^2^Vascular Health Research Center of Peking University Health Science Center, Beijing, China

Correspondent to Hua Luo.

E‐mail: guaiguaijiao152@163.com, luoxiaoer@hotmail.com

**Abstract:** Pulmonary arterial hypertension crises due to congenital heart disease are a relatively rare management challenge in the field of structural cardiology. The swift escalation of pulmonary arterial pressures can precipitate severe hypoxemia, acute right heart failure, MODS, and sudden death. With high misdiagnosis rates and low resuscitation success, the mortality rate is near 50%. Currently, there's a global absence of standardized and unified comprehensive treatment. We attempted to employ the "Bundle (Basic, Unique, Normal, Learning, Estimate) " treatment strategy to reduce pulmonary arterial hypertension and improve MODS, aiming to mitigate the adverse outcomes of the crisis. We report the case of a 30‐year‐old parturient at 35 + 3 weeks gestation with a history of atrial septal defect. She was admitted for hypoxemia and, post‐emergency cesarean, developed refractory hypoxemia and shock. Echocardiography revealed a pulmonary arterial systolic pressure of 109 mmHg and a right ventricular size of 84*56 mm. After excluding pulmonary thromboembolism via CTPA, we diagnosed a pulmonary arterial hypertension crisis. We immediately initiated the 'Bundle' strategy encompassing six key interventions: dynamic monitoring of blood gas analysis and BNP (Basic); continuous intravenous infusion of treprostinil as a unique targeted pharmacotherapy (Unique); standardized circulatory and respiratory support integrating ECMO, CRRT, and mechanical ventilation (Normal); rapid reduction of PVR under Swan‐Ganz catheter guidance (Direction); understanding and intervening in potential triggers such as stress, pain, and gestational complications (Learning); and dynamic echocardiographic assessment of right ventricular function for prognosis (Estimate). Following this comprehensive approach, the patient's pulmonary arterial systolic pressure decreased to 77 mmHg, with significant improvements in oxygenation and hemodynamic stability. ECMO was successfully discontinued on day 11. We consider that the "Bundle" strategy effectively stabilized the patient's vital signs, alleviating the pulmonary arterial hypertension crisis and enhancing outcomes for critically ill parturients with structural heart diseases.

## 113. Critical Care Medicine Progress in Diagnosis and Treatment of Pulmonary Hypertension Crisis

Luo H^1,2^, Wang X^1^, Lin Y^1^, Zhang S^1^, Tu Y^1^, Huang B^1^

^1^ICU of Peking University of Shenzhen Hospital, Shenzhen, China, ^2^Vascular Health Research Center of Peking University Health Science Center, Beijing, China

Correspondent to Hua Luo.

luoxiaoer@hotmail.com

There is not enough systematic epidemiological data on pulmonary hypertension crisis (PHC). PHC progresses rapidly and is easily misdiagnosed as acute pulmonary embolism, left heart failure or ARDS. Its treatment is extremely difficult and expensive, easy to cause many disputes. Even in regions with some knowledge of the disease, treatment methods are often limited to mechanical ventilation and poor efficacy traditional medication, such as nitroglycerin, prostaglandin, and calcium antagonists. The poor bedside testing methods and low diagnostic capabilities also severely limit the understanding and treatment of this disease. Our team is one of the earliest teams in China has initiated a series of research on comprehensive treatment strategies for this disease. Since 2008, what we have made the most significant change in recognition is to treat essentially pulmonary vascular diseases as circulatory diseases rather than respiratory diseases. We have been the first to achieve a transition from respiratory support to circulatory support, no longer relying solely on ventilators to treat hypoxemia, but actively using targeted drugs to improve PVR and right heart function. In 2012, we conducted a study to explore PHC risk factors in China and drew a preliminary conclusion that congenital heart disease, SLE, and PTE are the top three diseases prone to leading to PHC. Furthermore, we proposed pulmonary protective ventilation strategy for PHC. In 2014, the BUNDLE comprehensive treatment strategy was initially formulated. With the development of critical care ultrasound and PICCO monitoring technology, in 2019 we summed up protective measures for right heart dysfunction and volume management strategies for obstructive shock related to pulmonary hypertension. In 2020, targeted therapy drugs combined with ECMO technology to treat refractory hypoxemia. All the measures above gradually have improved the theoretical system of BUNDLE comprehensive management strategy.

## 114. Telomere Dynamics: Pharmacological Target for Hypobaric Hypoxia Pathophysiology

Vibhuti A^1^, Kumar R^2^, Paulose A^3^, Mishra A^4^, Qadar Pasha M^5^

^1^SRM University, Delhi NCR Sonepat, India, ^2^SRM University, Delhi NCR Sonepat, India, ^3^SRM University, Delhi NCR Sonepat, India, ^4^IGIB, CSIR, Delhi, India, ^5^Institute of Hypobaric Hypoxia, New Delhi, India

Extreme environments at High‐altitude (HA) lead to the expression of multiple cellular proteins that involve in pathophysiological manifestations. Hypobaric hypoxia at HA generates reactive oxygen species (ROS) that can damage telomeres and disturb normal physiological processes. Telomeres are a novel and promising target to explicate the phenomena of adaptation or maladaptation at HA. The endurance of an individual may be prognosed in advance by studying the telomeres of the people before their visit to HA. The telomere complex comprises multiple proteins, of which, tankyrase (TNKS) is actively involved in DNA damage repairs. Understanding the roles of telomeres, telomerase, and TNKS could ameliorate physiological issues experienced at HA. We investigated this system in three groups, the healthy sojourners named HAPE‐resistant (HAPR‐r), healthy natives (HLs) and the HA Pulmonary Edema patients (HAPE‐p). The telomere length was shorter in HAPE‐p compared to HAPE‐r (P = 0.03) and HLs (P = 4.25E‐4). The telomerase activity was significantly higher in HAPE‐p compared to both HAPE‐r (P = 0.01) and HLs (P = 0.001). HAPE‐p had the lowest TNKS levels (0.186 ± 0.031 ng/μl) and the highest telomerase activity (0.0268 amoles/μl). The expression was upregulated by 9.27 folds in HAPE‐p (P = 1.01E‐06) and downregulated in HLs by 3.3 folds (P = 0.02). The findings indicated the association of telomere, telomerase and TNKS with hypoxia‐induced sicknesses/acclimatization. To understand the roles of Telomeres, understanding their regulation, such as through epigenetics, is critical. The epigenetic modifications promote red blood cell synthesis and more effective oxygen utilization, which are essential for survival in HA settings. Thus, correlations between the telomere system and methylation could provide greater insight into HA sickness. Moreover, these correlations may help us better understand the intricate biological reactions to hypoxia and create preventative and therapeutic measures.

## 115. The Relationship Between TAPSE/sPAP Ratio And REVEAL Lite 2 Score as Predictors of Mortality in Precapillary Pulmonary Hypertension: A Single‐Center Experience

Varga A^1^, Balasa A^1^, Moldovan D^1^, Tilea I^1^

^1^“George Emil Palade” University Of Medicine, Pharmacy, Science And Technology Of Targu Mures, Targu Mures, Romania

**Background:** Pulmonary hypertension (PH) is a severe condition characterized by elevated pulmonary arterial pressure, often associated with poor outcomes. This study aimed to evaluate the relationship between echocardiographic parameters, specifically the tricuspid annular plane systolic excursion (TAPSE) to systolic pulmonary arterial pressure (sPAP) ratio, and the REVEAL Lite 2 score in predicting mortality risk in patients with precapillary PH, namely pulmonary arterial hypertension (PAH) and chronic thromboembolic pulmonary hypertension (CTEPH).

**Methods:** In this retrospective analysis, data from 65 adult patients were reviewed: 42 diagnosed with PAH (female‐to‐male ratio 1.6:1) and 23 with CTEPH. The study was conducted in a single Romanian center over eight years, following the 2015 and 2022 ESC/ERS Guidelines for PH diagnosis and treatment. All patients received standard care, including approved medical therapies available in Romania. PAH patients were younger (mean age 49.75 ± 15.65 years) than those with CTEPH (mean age 62.95 ± 6.5 years). Among PAH patients, 22 had PAH associated with congenital heart disease. Descriptive statistics and Pearson correlation analysis assessed the relationship between TAPSE/sPAP and REVEAL Lite 2 scores. Regression models were applied to the PAH and CTEPH subgroups.

**Results:** A weak but statistically significant negative correlation (r = ‐0.27, p < 0.0001) was identified between the TAPSE/sPAP ratio and REVEAL Lite 2 scores across the entire cohort. Subgroup analysis revealed a stronger correlation in CTEPH patients (r = ‐0.44, p < 0.0001) compared to PAH patients (r = ‐0.21, p = 0.0006). These results suggest that the TAPSE/sPAP ratio is a more robust predictor of increased REVEAL Lite 2 scores in CTEPH patients than in those with PAH.

**Conclusion:** The TAPSE/sPAP ratio is a valuable echocardiographic marker for predicting mortality risk in PH patients, particularly those with CTEPH. The findings support using combined echocardiographic measures and risk assessment tools, such as the REVEAL Lite 2 score, to enhance predictive accuracy in clinical practice.

## 116. Atrial Septostomy in Patients With Severe Pulmonary Arterial Hypertension

Nguyen L H^1,2^, Nguyen T M L^1,2^, Vuong A T^2^, Nguyen L V^1,2^

^1^Hanoi Medical University, Hanoi, Vietnam, ^2^Hanoi Medical University Hospital, Hanoi, Vietnam

Patients with severe pulmonary arterial hypertension (PAH) have a poor prognosis, especially those without intracardiac shunt. Atrial septostomy (AS), which creates communication between the right and left atrium, is considered a palliative approach for severe PAH patients with refractory right heart failure. Despite its benefits in improving patients' symptoms, this procedure is still underused due to the fear of a high complication rate related to the procedure. We presented a case series of five patients with primary PAH who received AS at the Cardiovascular Center of Hanoi Medical University Hospital.

Data was collected, including time from diagnosis, clinical symptoms, oximetry, functional class, echocardiogram features, biomarkers before and after AS, and hemodynamic measurements at the time of the procedure.

Procedures were successful in all patients. After the intervention, all patients showed improvement in signs and symptoms of right heart failure, decreasing the need for hospitalization. Patients were followed for a median of 7 months (interquartile range 1 ‐ 11 months). One patient needed a repeat AS after six months after the first attempt. Two patients died nine months and 11 months after AS due to peritoneal fluid infection and a pulmonary arterial hypertension crisis. The three patients are stable with medical treatment. This single‐center experience suggests that early AS, in addition to pharmacotherapy, is safe and warrants consideration for symptomatic patients with severe PAH who do not have an intracardiac shunt.

## 117. Creating Guidelines for the Safe Transport of Critically Ill Patients With Pulmonary Hypertension

Champion C^1^, Dryzer S^1^, Coleman R^2^, Chartan C^1^

^1^Dell Children's Center for Pulmonary Hypertension, Dell Children's Medical Center, The University of Texas at Austin, Dell Medical School, Austin, USA, ^2^Texas Children's Hospital, Baylor College of Medicine, Houston, USA

**Background:** The safe and effective transportation of critically ill patients, particularly those with pulmonary hypertension, pose significant challenges to healthcare providers and centers that manage these very complex patients. Children with pulmonary hypertension have elevated pulmonary pressures and may have associated right ventricular dysfunction which could increase their risk for cardiopulmonary decompensation during transport.

**Methods:** A multidisciplinary, multispecialty panel of pulmonary hypertension specialists, intensivists, and transport experts convened to determine all aspects of high‐risk transports of this critical and vulnerable patient cohort. Determination of appropriate transport modalities, assuring adequate oxygenation and hemodynamic stabilization and planning for a crisis event were all decided to be the most critical planning aspects.

**Results:** The team created a pre‐transport checklist to be utilized during a huddle that occurs before the event. This huddle was developed to determine current medical condition and stability, assure appropriate intravenous access points, acquire recent medication lists and dosing and construct plans for an emergency. An algorithmic guideline was also crafted to include pro‐active management and to allow for a shared mental model for the members involved in the actual transport.

**Conclusion:** Transport of the pediatric patient with pulmonary hypertension requires significant preparation by all the teams involved, anticipatory expectant management and rapid intervention when deterioration occurs during this very dynamic process. Clear planning, transparent communication with all members of the multidisciplinary team and utilization of a consensus expert guideline can optimize outcomes for this critical population.

## 118. Multiparametric Risk Predictors of Morbidity and Mortality in Pulmonary Hypertension: A Single‐Centre Data

Tokgoz H^1^, Tanyeri S^2^, Hakgor A^3^, Kultursay B^4^, Keskin B^2^, Sekban A^1^, Bulus C^1^, Sirma D^1^, Kulahcioglu S^1^, Karagoz A^1^, Tanboga İ^5^, Ozdemir N^1^, Kaymaz C^1^

^1^Kosuyolu Heart Research And Education Hospital, Istanbul, Turkey, ^2^Kocaeli City Hospital, Cardiology, Kocaeli‐Turkey, Koaceli, Turkey, ^3^Medipol University, Cardiology, Istanbul‐Turkey, Istanbul, Turkey, ^4^Tunceli State Hospital, Cardiology, Tunceli‐Turkey, Tunceli, Turkey, ^5^Hisar Intercontinental Hospital, Cardiology, Istanbul‐Turkey, Istanbul, Turkey

**Background:** In this retrospective analysis of a single‐centre study on PH, we aimed to evaluate the clinical, echocardiographic, haemodynamic and neurohumoral predictors of morbidity and mortality.

**Methods:** This study included 1071 PH patients enrolled in the Evaluation of Pulmonary Hypertension Risk factors Associated with Survival study (EUPHRATES).ESC/ERS 2022, French Registry, COMPERA 2.0 and REVEAL lite 2 models were used for risk predictions.

**Results:** Median age was 56(38‐64) years, 62% of patients were female. Idiopathic pulmonary arterial hypertension (IPAH), PAH‐associated with congenital heart diseases (CHD‐PAH), PAH‐associated with connective tissue diseases (CTD‐PAH), group II, III, IV and V PH were documented in 24%, 28%, 4.6%, 4.3%, 11%, 27% and 0.7% of overall patients, respectively. Background mono, dual and triple combinations were noted in 46%, 33% and 21% of patients, respectively.Macitentan was documented in 46% of dual, and 50.3% of triple combinations. Median follow‐up time was 599 (126‐1568) days. Mortality and CEP rates were 41% and 73% in overall PH, 41% and 79% in IPAH, 32% and 63% in CHD‐PAH, 67% and 88% in CTD‐PAH, 35% and 70% in group II, 64% and 73% in group III, and 39% and 79% in group IV, respectively. CHD‐PAH related to a higher overall survival and CEP‐free survival compared with other PH groups. Survival was associated with baseline FC and 6MWD in overall PH and all PAH subgroups, NT‐proBNP and TAPSE/PASP ratio in PH, IPAH and CHD‐PAH, RA area in PH and IPAH, RAP in PH, IPAH, CHD‐PAH and group IV PH, RA–PCWP difference and SV index in PH and IPAH, and mixed venous O2 saturation in PH, IPAH and group IV PH. PVR and its ratio to SVR also related with survival in IPAH.

**Conclusions:** Our single‐centre data revealed current status concerning the multiparametric risk predictions for morbidity and mortality in PH and its subsets.

## 119. Shared Decision‐Making in the Treatment of Pulmonary Arterial Hypertension

Giri P^1^, Merrill‐Henry J, Rajah T, Sullivan D, Oyoyo U, Elwing J, Balasubramanian V

^1^Renew Medical Group, Redlands, USA

**Introduction:** Selection of appropriate PAH therapy(ies) is a complex medical decision that may factor in disease severity, efficacy and side‐effects of therapy, as well as adherence, quality‐of‐life, values, and personal preferences of patients. Shared decision‐making (SDM) is critical in this process. Multiple medical societies endorse the salutary effects of SDM on patient‐centered care and health outcomes. Despite this, uptake of SDM in clinical practice remains low. SDM remains inadequately evaluated in the context of PAH. We surveyed SDM among patients and clinicians during treatment selection in PAH.

**Methods:** Using the Ottawa Decision‐Making Framework guidelines, we conducted a survey on patients and clinicians to assess shared decision‐making during selection of PAH therapy. This was conducted at the Pulmonary Hypertension Association's International PH Conference and Scientific Sessions in Atlanta, Georgia between June 9‐June 11, 2022. We used QualtricsXM to conduct the survey and R, version 4.2.3., for data analysis.

**Results:** A total of 89 participants (51 patients and 38 clinicians) responded to the survey. Most patients (95.6%) and clinicians (96.4%) selected shared decision‐making as their preferred role, p = ns. “Decisional Conflict” was found in 38.4% patients and 8.3% clinicians, p = 0.002. The top three factors considered by patients and clinicians varied significantly with more patients choosing “Quality of Life” (80% vs. 55%, patients vs. clinicians, p < 0.01), and “Side‐effect Profile” (45% vs. 30%, patients vs. clinicians, p < 0.01), and more clinicians choosing “Risk of Disease Progression” (70% vs. 30%, clinicians vs. patients, p < 0.01).

**Conclusions:** Although shared decision‐making is the preferred role by both patients and clinicians during PAH‐therapy selection, “Decisional Conflict” exists, and values and preferences between patients and clinicians differ widely, highlighting the need for further evaluation of SDM in this setting.

## 120. Pulmonary Arterial Hypertension and Pregnancy: A Series of Seven Cases

Yahiaoui R^1^

^1^Algiers University Of Medecine, University Hospital Of Béni‐messous,algeria, Algiers, Algeria

**Introduction:** Pregnancy in women with pulmonary hypertension (PH) is associated with high fetal‐maternal morbidity and mortality rates. This is due to the physiological changes that occur during pregnancy and the peripartum period.

**Objectives:** to describe the particularity of PH in pregnant women and the importance of multidisciplinary management

**Materials and Methods:** We report seven patients with pulmonary hypertension (PH) who presented with pregnancy and were followed in the PH unit of the pulmonology department between 2016 and 2024.

**Results:** All these patients were diagnosed by right heart catheterization: two cases of PAH associated with congenital heart disease, two cases of idiopathic pulmonary arterial hypertension (iPAH), one case of PAH associated with lupus, one case associated with Gaucher's disease, and finally one case of chronic thromboembolic PH. The mean maternal age was 26.4 ± 2.0 year. Mean pulmonary artery pressure was greater than 50 mm Hg in 100% of cases, right ventricular systolic pressure was < 50 mm Hg in 59.6% of patients, 50–70 mm Hg in 28.5%, and > 70 mm Hg in 11.9%. NT‐Pro BNP was greater than 900 pg/l. PH had been diagnosed before pregnancy in 80% of patients. Caesarean section under epidural anesthesia was performed in four patients who continued their pregnancies until the 33rd week, in collaboration with obstetricians and anesthesiologists. Complications included maternal death (0.33%): one during early abortion, and the other during delivery by postpartum hemorrhage. Fetal prematurity was present in all newborns, requiring a stay in the neonatal unit.

**Conclusion:** Pregnancy is contraindicated in patients with pulmonary hypertension. However, after providing clear information on maternal and fetal risks, and on the possibilities of medical termination of pregnancy, patients with PAH wishing to continue a pregnancy must be managed in an appropriate hospital center using a multidisciplinary approach with experts in pulmonary hypertension, obstetricians, and anesthesiologists.

## 121. Balloon Pulmonary Angioplasty for Inoperable or Residual/Recurrent Chronic Thromboembolic Pulmonary Hypertension: A Single‐Center Experience

Ataş H^1^, Mutlu B^1^, Akaslan D^1^, Kocakaya D^1^, Mustayev U^1^, Yildizeli B^1^

^1^Marmara University, Istanbul, Turkey

Patients with inoperable chronic thromboembolic pulmonary hypertension (CTEPH) are frequently managed with pulmonary arterial hypertension‐specific therapies. Nevertheless, the majority of these patients continue to experience symptoms despite medical treatment. Balloon pulmonary angioplasty (BPA) has emerged as a promising therapeutic option for patients with inoperable CTEPH. This study aims to present the initial experience with BPA at a tertiary referral center specializing in CTEPH.

A total of 70 consecutive patients, who underwent 314 BPA sessions, were included in the study. All patients underwent a detailed examination, including 6‐minute walking distance (6MWD), and right heart catheterisation at baseline and 3 months after the last BPA session.

The mean age of the patients was 54.7 ± 16 years. Forty‐three (43) patients had inoperable CTEPH and 27 patients had residual or recurrent CTEPH post‐pulmonary endarterectomy (PEA). The 6MWD increased from a mean of 314 ± 124 to 375 ± 99 m (p < 0.001), and NT pro‐BNP reduced from a mean of 1721 to 731 pg/mL (p = 0.001). The number of patients who required supplemental oxygen decreased from 12 (17%) to five (7%) (p < 0.001). The mean pulmonary artery pressure decreased from a mean of 43.8 ± 13.5 to 33 ± 10.3 mmHg (p < 0.001), the pulmonary vascular resistance decreased from a mean 7.7 ± 4.7 to 4.7 ± 2.9 Wood units (p < 0.001), and the cardiac index increased from a mean 2.5 ± 0.7 to 2.7 ± 0.6 L/min/m2 (p < 0.001).

Balloon pulmonary angioplasty improved hemodynamics, 6‐minute walk distance (6MWD), and reduced the need for supplemental oxygen. This demonstrated an acceptable risk‐benefit profile in patients with inoperable CTEPH as well as those with residual or recurrent CTEPH.

## 122. Thrombospondin‐1 as a Biomarker of Right Ventricular Remodeling and Dysfunction in Pulmonary Vascular Disease

Rosen D^1^, Naranjo M^2^, Tuhy T^1^, Balasubramanian A^1^, Kauffman M^1^, Kolb T^1^, Mathai S^1^, Simpson C^1^, Zimmerman S^3^, Tedford R^4^, Hsu S^5^, Damico R^6^, Hassoun P^1^

^1^Division of Pulmonary and Critical Care Medicine, Johns Hopkins University School of Medicine, Baltimore, USA, ^2^Division of Thoracic Medicine & Surgery, Lewis Katz School of Medicine at Temple University, Philadelphia, USA, ^3^Department of Radiology and Radiological Science, Johns Hopkins University School of Medicine, Baltimore, USA, ^4^Division of Cardiology, Medical University of South Carolina, Charleston, USA, ^5^Division of Cardiology, Johns Hopkins University School of Medicine, Baltimore, USA, ^6^Division of Pulmonary, Critical Care and Sleep Medicine, University of Miami, Miami, USA

**Background:** There is a need for non‐invasive biomarkers of right ventricular (RV) maladaptation, as this represents the primary determinant of outcomes in pulmonary arterial hypertension (PAH).

**Objectives:** We hypothesized that serum levels of thrombospondin‐1 (TSP1) would be associated with abnormal RV remodeling, reduced RV function, and clinical outcomes based on its proposed role in PAH pathogenesis.

**Methods:** In a prospective single‐center study (NIH/NHLBI‐sponsored study ‐ NCT04610788) with 73 suspected PAH patients who underwent multi‐beat RV pressure‐volume (PV) loop assessment and cardiac MRI (CMR), serum TSP1 was analyzed in association with PAH diagnosis, gold‐standard measurement of RV‐pulmonary arterial (PA) coupling using multi‐beat end‐systolic elastance over arterial elastance (Ees/Ea), diastolic function using end‐diastolic elastance (Eed), and RV remodeling using RV end‐diastolic volume (RVEDV), RV mass, and ventricular mass index (VMI). Cox proportional hazard models were performed to evaluate TSP1 predicting time to clinical worsening adjusted for age and sex.

**Results:** TSP1 levels highly discriminated PAH versus no PH with area under the curve (AUC) 0.94, significantly higher than NT‐proBNP which corresponded to AUC 0.61 (p‐value < 0.001). TSP1 was negatively associated with Ees/Ea (p < 0.01) and positively associated with RVEDV (p = 0.018), RV mass (p < 0.01), and VMI (p < 0.001) in linear regression models. In Cox proportional hazard models, an increase in TSP1 of 0.1 µg/ml corresponded to a hazard ratio (HR) of 1.07 (CI 1.02 – 1.14; p = 0.01) in predicting time to clinical worsening. Inclusion of TSP1 in Cox models improved model performance when added to REVEAL 2.0 score (p = 0.043, likelihood ratio test).

**Conclusion:** This study supports the role of TSP1 as a biomarker of RV dysfunction, pathologic RV remodeling, and PAH disease screening. Future research is needed to evaluate whether TSP1 levels are associated with survival in larger studies.

## 123. Spatial Profiling Identifies Localized Changes In Gene Expression in Pulmonary Vascular Remodeling in the Sugen Hypoxia Rat Model

Klinger J^1^, Bharati A^1^, Pereira M^1^, Yang D^1^, Liang O^1^, Bertone P^1^, Brodsky A^1^

^1^Rhode Island Hospital, Brown University, Providence, United States

**Rationale:** Numerous changes in the lung transcriptome have been identified in patients and animal models of pulmonary arterial hypertension (PAH), but it is unclear which changes are responsible for pulmonary vascular remodeling. This study used spatial transcriptomics to identify changes in gene expression localized to diseased vessels.

**Methods:** Male Sprague‐Dawley rats were treated with Sugen5416 (20 mg/kg) and exposed to 3 weeks of hypoxia (10% O2) followed by 2 weeks recovery in normoxia (SuHx‐PH). Control rats were treated with vehicle and kept in normoxia. Lungs from 6 SuHx‐PH and 6 controls were fixed for histological examination and spatial transcriptomics utilizing the 10X Visium sequencing platform. Downstream analysis was conducted in R using the Seurat package. Genes of interest were further investigated using previously published single‐cell RNA sequencing databases in SuHx‐PH rats from Hong et al.

**Results:** The 25 most differentially expressed genes were chosen based on log‐fold change value. Expression of Cyp1b, Cyp1a, Ahrr, Nqo1, Tm4sf1, C6, and Lcn2 were increased in SuHx‐PH rat lungs and were nearly undetectable in control lungs. Down regulated genes include Ca4, Gpihbp1, Ighm, Fmo1, Slc7a10, Gpx2, and Col6a. Interrogation of scRNAseq data bases from SuHx‐PH rat lungs revealed that upregulated genes were seen primarily in endothelial arterial type 1 and type 2 cells, and in fibroblasts. Increased expression of Ahrr and Cyp1 localized to areas of pulmonary vascular remodeling.

**Conclusions:** Spatial transcriptomics offers a unique approach to identifying gene expression associated with the pulmonary vascular remodeling in PH. Ahr and Cyp1b1 expression are increased in human pulmonary arteries in PAH and the Ahr/Cyp1 pathway has been shown to regulate estrogen metabolism and synthesis of prostacyclin and leukotrienes. Considering the important role of these mediators in pulmonary vascular remodeling, targeting the Ahr‐Cyp1 pathway may be a novel approach to attenuating pulmonary vascular remodeling in PAH.

## 124. Inhaled Treprostinil for the Treatment of Pulmonary Arterial Hypertension in Intermediate‐High Risk Patients: A Sub‐Group Analysis of the TRIUMPH Study

McLaughlin V^1^, Estrada R^2^, El‐Kersh K^3^, Hong T^4^, Thrasher C^5^, Broderick M^5^, Argula R^6^

^1^University of Michigan, Ann Arbor, United States, ^2^UT Health San Antonio, San Antonio, United States, ^3^University of Arizona, Phoenix, United States, ^4^North Carolina State University, Raleigh, United States, ^5^United Therapeutics Corporation, Research Triangle Park, United States, ^6^Medical University of South Carolina, Charleston, United States

**Background:** Proceedings from the 7th World Symposium on Pulmonary Hypertension recommend inhaled prostacyclins for intermediate‐low risk patients with PAH. The TRIUMPH study demonstrated the safety and efficacy of inhaled treprostinil (iTre) in patients with PAH; this post‐hoc analysis evaluated iTre's safety and efficacy in the intermediate‐high risk patients from TRIUMPH.

**Methods:** TRIUMPH was a 12‐week, double‐blind, placebo (PBO)‐controlled study in patients with PAH on background monotherapy. Patients were included in this analysis if they had all COMPERA 2.0 components (6MWD, NT‐proBNP, functional class) at baseline and week 12 (W12). Least‐squares mean change from baseline to W12 in 6MWD, COMPERA 2.0 and REVEAL Lite 2 scores were analyzed using Mixed Model Repeated Measurement; AEs were compared using Fisher's Exact Test.

**Results:** At baseline, 47% of study patients were intermediate‐high risk (n = 48 iTre, n = 52 PBO), most were functional class III (97%), receiving bosentan (64%), with idiopathic (59%) or connective tissue disease‐PAH (32%). The median (IQR) dose of iTre at W12 was 9 (9,9) breaths four times daily. Change in 6MWD at W12 was +32 m for iTre vs ‐3m for PBO (p = 0.009). Change in COMPERA 2.0 score was ‐0.5 for iTre vs ‐0.1 for PBO (p = 0.0195); more iTre patients improved (36% improved, 0% worsened) vs PBO (22% improved, 7% worsened). Change in REVEAL Lite 2 at W12 was ‐1.5 for iTre vs ‐0.2 for PBO (p = 0.0014); more iTre patients improved (61% improved, 9% worsened) vs PBO (35% improved, 35% worsened). There were no significant differences in AEs between iTre and PBO, except for flushing (17% vs 0%).

**Conclusions:** Considering the limitations of post‐hoc analyses, intermediate‐high risk patients from TRIUMPH who received iTre had significant improvements in 6MWD, COMPERA 2.0, and REVEAL Lite 2 scores, with similar AEs compared to PBO. These data demonstrate iTre's safety and efficacy in intermediate‐high risk patients with PAH.

## 125. Qualitative Assessment of Pulmonary Arterial Hypertension and Cancer: Are They Similar?

El‐Kersh K^1^, Coyle C^2^, Shafrin J^3^, Zawadzki N^3^, Burke T^2^, Watzker A^2^, Zhang S^3^, Dalal D^3^, Lautsch D^2^

^1^University of Arizona College of Medicine, Phoenix, USA, ^2^Merck And Co., Inc., Rahway, USA, ^3^FTI Consulting, Los Angeles, USA

**Background:** Analogue diseases, or conditions that share key similarities, can help underscore the unique burden of rare diseases, such as pulmonary arterial hypertension (PAH). This study identified cancer analogues to PAH using an empirical framework.

**Methods:** A multi‐step approach was used: (1) define cancer inclusion/exclusion criteria: solid, malignant tumors with ≥ 3 approved anti‐cancer therapeutics (not supportive, adjuvant, and radiotherapy) in the United States (US), of which ≥ 1 therapeutics was approved 2013‐2023, is not sex‐specific, exclusively pediatric, secondary to other conditions or treatment, has an annual US incidence 1,000‐95,000 patients; (2) identify relevant dimensions and disease‐level metrics (DLMs): epidemiology (e.g., demographics), clinical (e.g., mortality), therapeutic landscape (e.g., number of generics), healthcare resource utilization (HCRU; e.g., hospitalizations); (3) identify potential cancer analogues through screening of US Food and Drug Administration drug indications; and (4) conduct a targeted literature review to extract data according to DLMs for cancers meeting all selection criteria. Cancer analogues were ranked based on the absolute difference between each cancer's and PAH's DLMs, within each dimension.

**Results:** Fourteen cancers met criteria for inclusion, by site: lung (n = 3); endocrine (n = 2), gastrointestinal (n = 2) kidney & bladder (n = 2), skin (n = 2), brain (n = 1), head & neck (n = 1), liver (n = 1). Closest analogues across dimensions were well‐differentiated thyroid cancer (wDTC) (#1 in five DLMs), gastrointestinal stromal tumor (GIST) (#1 in four DLMs), and medullary thyroid cancer (MTC) (#1 in three DLMs). Within each dimension the ranking was: wDTC and MTC (epidemiology), GIST (clinical), non‐small cell carcinoma ALK+ and renal cell carcinoma (therapeutic landscape), and GIST (HCRU). Survival/mortality and annual hospitalization were DLMs with the highest number of similarities shared between cancers and PAH.

**Conclusion:** This study revealed that PAH shares numerous similarities with cancers, highlighting its multidimensionality and the potential usefulness of cancer analogues in increasing recognition and understanding of PAH among healthcare stakeholders.

## 126. Intraparenchymal Vascular Volumes Post Pulmonary PTE in Patients With CTEPH

Rahaghi F^1^, Mylvaganam R^2^, Nardelli P^3^, San Jose Estepar R^3^, Washko G^1^, Schimmel D^4^, Malaisrie C^5^, Chiu S^5^, Cuttica M^2^, San Jose Estepar R^3^

^1^Division of Pulmonary and Critical Care, Brigham and Women's Hospital, Harvard Medical School, Boston, United States, ^2^Division of Pulmonary and Critical Care, Northwestern University; Feinberg School of Medicine, Chicago, USA, ^3^Department of Radiology, Brigham and Women's Hospital, Harvard Medical School, Boston, United States, ^4^Division of Cardiovascular Medicine, Northwestern University; Feinberg School of Medicine, Chicago, United States, ^5^Division of Cardiac Surgery, Department of Surgery, Northwestern University Medical Center, Chicago, United States

**Introduction:** Pulmonary Thromboendarterectomy (PTE) is the treatment of choice for Chronic Thromboembolic Pulmonary Hypertension (CTEPH). CT angiography is an integral tool in both the diagnostic work‐up of patient with CTEPH as well as in assessing for residual disease post‐surgery. In this work we sought to examine the change in the intraparenchymal vascular volume post PTE.

**Methods:** Patients with pre and post CT imaging with adequate resolution were enrolled from consecutive patients undergoing PTE at Northwestern University Medical Center for the diagnosis of CTEPH. CT scans with thickness of 2 mm or less obtained prior to surgery were used as baseline. Follow‐up CT imaging was obtained usually at 6 month post PTE. Vascular reconstruction was performed using automated methods previously described. Vascular volume is represented as ml of vascular per liter of lung volume. Data is represented as median and upper/lower quartiles with comparisons made using the Wilcoxon Signed‐Rank Test. All analysis was performed using R version 4.1.

**Results:** There were 38 patients identified with both baseline and follow‐up CT imaging. The baseline total vascular volume (TBV) was 54.9[48.6‐65.3]. At follow up this has increased to 64.5 [56.9‐71.0], p = 0.0007.

**Conclusion:** Automated AI based methods were used to perform intraparenchymal vascular reconstructions, which demonstrated an increase in intraparenchymal vascular volume. This could serve as a potential marker of the vascular bed response to interventions treating CTEPH.

## 127. Burden and Unmet Needs Among Women With Pulmonary Arterial Hypertension (PAH): A Multinational Survey Study

Vaidya A^1^, Delcroix M^2^, Thakur T^3^, Watzker A^3^, O'Hara M^4^, Silber A^4^, Abilash V^4^, Preston I^5^, Lautsch D^3^, McLaughlin V^6^

^1^Temple University, Philadelphia, United States, ^2^University Hospitals of Leuven, Leuven, Belgium, ^3^Merck & Co., Inc, Rahway, United States, ^4^Trinity Life Sciences, Waltham, United States, ^5^Tufts University School of Medicine, Boston, United States, ^6^University of Michigan‐Ann Arbor, Ann Arbor, United States

**Background:** Pulmonary arterial hypertension (PAH) is a progressive disease that disproportionately affects women. This study evaluates the burden of PAH among women of childbearing and upbringing age via a multi‐country survey.

**Methods:** This was an interim descriptive analysis of data from online survey of women (age 21‐50) with PAH in United States (US) and United Kingdom (UK). De novo questions derived from findings from a previous qualitative phase and validated instruments (SF‐12, emPHasis‐10, WPAI) were included. Institutional Review Board/Ethics Committee approval/exemption was obtained in all countries.

**Results:** This interim analysis includes 120 US and 30 UK patients. Final results (expected n ~ 200 patients, 6 countries, includes regression model) will be presented at the conference.

**Humanistic burden:** Patients reported PAH impacting their social life (US = 57%; UK = 37%), physical activity/exercise (US = 82%; UK = 37%) and sleep cycle (US = 50%, UK = 43%) over past 12 months. Patients reported missing 4.5 and 8.2 mean hours of work due to PAH out of 27.7 and 26 hours in last seven days of actual work, in US and UK, respectively.

**Clinical burden:** Mean score on emPHasis‐10 was 31.5 in US and 32.9 in UK, with always needing rest during the day (41%) and breathlessness walking a flight of stairs (37%) being most burdensome symptoms in US and feeling very frustrated by breathlessness (17%) and breathlessness walking a flight of stairs (17%) in UK. On SF‐12, 12% US and 17% UK patients reported their general health as poor while no patients reported very good health.

Economic: High out of pocket costs (e.g., visits, medications) over 12‐months were reported by 50% patients in US and 97% in UK.

**Conclusions:** This study confirms that the burden women living with PAH experience is high and multifaceted. The data uncover a need for future interventions and guidelines that address this unique burden.

## 128. Exploring the Complexity and Heterogeneity in Pulmonary Arterial Hypertension Clinical Trials and the Impact on Clinical Outcome of Six‐Minute Walk Distance: A Systematic Literature Review

Ramani G^1^, Bali V^2^, Black H^2^, Redwood B^3^, Zile I^3^, Humphries A^3^, Lautsch D^2^

^1^Division of Cardiovascular Medicine, Department of Medicine, University of Maryland School of Medicine, Baltimore, United States, ^2^Merck & Co., Inc., Rahway, United States, ^3^Adelphi Values PROVE, Bollington, United Kingdom

Objective: Investigate changes in expected six‐minute walk distance (6MWD) improvement over time due to evolving care standards and diverse background therapies in trials.

**Methods:** A systematic review was conducted in Embase, MEDLINE, CENTRAL, Cochrane Database of Systematic Reviews, PsychInfo and Cochrane Library for articles published before 31‐May‐2024, as well as conference abstracts (01‐JAN‐2020 – 10‐JUN‐2024). Prespecified inclusion criteria were: PAH patients age ≥ 18; FDA approved PAH‐pharmacologic interventions; clinical outcomes; and prospective randomized, controlled clinical trial (RCT) study designs.

**Results:** The SLR identified 93 trials, phase 2 – 4 (56 RCTs, 8 open‐label extension phases of the captured RCTs and 29 either on‐going or without published results). Here we present data from the placebo‐controlled phase 3/3b trials that reported on regulatory‐approved doses (n = 17: 16 phase‐3, 1 phase‐3b): 6MWD was the most commonly reported endpoint (in 15 trials), though only 9 trials reported about statistical significance (4 trials having p < 0.05, 5 trials having p > 0.05). Trials during 2006‐2014 often enrolled treatment‐naïve or moderately treated patients whereas recent trials (2015–2024) enrolled heavily pre‐treated patients. Trial duration ranged from 3 days (IV Iloprost) to 3.7 years (Treprostinil), with 6MWD typically assessed after 12‐16 weeks in the majority of early trials (pre‐2015) versus 16‐48 weeks in later trials (from 2015 onwards). Control‐adjusted mean 6MWD change from baseline (CFB) data in these trials were as follows: inhaled Iloprost: ‐1.5 m (2009), Tadalafil: 14.0 m to 33.0 m (2009‐2024), Sildenafil: 13.1 m (2010), Ambrisentan: 31.0 m to 59.0 m (2008), Treprostinil: 8.0 m to 22.0 m (2013‐2020), Riociguat: 36 m (2013), Selexipag: ‐1.4 m to 12.0 m (median) (2015‐2021), Macitentan: 24.9 m to 25.4 m (2019‐2024), and Sotatercept: 40.8 m (2023).

**Conclusions:** Effects on 6MWD in clinical trials are influenced by both the interventions tested and background therapy. Earlier trials involved no background therapy, while contemporary trials enrolled patients on dual or triple background therapy.

## 129. Association of Pulmonary Artery Compliance and Adverse Cardiac Events

Mounsey L^1^, Nemeth S^1^, Kosyakovsky L^1^, McNeill J^2^, Picard M^3^, Pomerantsev E^3^, Ho J^1^

^1^Beth Israel Deaconess Medical Center, Boston, United States, ^2^Duke University, Durham, United States, ^3^Massachusetts General Hospital, Boston, United States

**Introduction:** Pulmonary artery compliance (PAC), the ratio of stroke volume to pulmonary artery pulse pressure, reflects the pulsatile component of right ventricular afterload. While worse PAC is associated with right heart failure and death in patients with pulmonary arterial hypertension, the association of PAC with clinical outcomes among individuals with a broader range of cardiopulmonary comorbidities is unclear.

**Methods:** We examined consecutive ambulatory and hospitalized patients undergoing clinically indicated right heart catheterization at a single center between 2005 and 2016. Multivariable Cox models were used to investigate the association of PAC with clinical outcomes including heart failure (HF) hospitalization and mortality. Analyses were stratified by presence and absence of pulmonary hypertension (PH) and by PH hemodynamic subtype.

**Results:** Among 7966 patients (mean age 63 years, 39% women), median PAC was 3.29 (IQR 2.19, 4.70) mL/mmHg. Over a median follow‐up of 7.4 years, there were 1,925 HF hospitalizations and 2,894 deaths. Across the whole sample, PAC was significantly inversely associated with mortality (HR 0.68 per 1‐SD higher PAC, 95% CI 0.65, 0.71, p < 0.001) and HF hospitalization (HR 0.60, 95% CI 0.57, 0.63, p < 0.001). In stratified analyses, PAC was significantly associated with HF hospitalization among those with and without PH and across all PH hemodynamic subtypes. PAC was associated with mortality in those with precapillary PH (HR 0.74, 95% CI 0.68, 0.81, p < 0.001), combined PH (HR 0.84, 95% CI 0.78, 0.9, p < 0.001) and without PH (HR 0.81, 95% CI 0.75, 0.89, p < 0.001).

**Conclusions:** Among patients undergoing clinically indicated right heart catheterization, lower PAC is associated with adverse outcomes irrespective of the presence or absence of PH and across all subtypes of PH. Our findings support potential clinical utility of PAC in risk stratification across a broad spectrum of cardiopulmonary disease.

## 130. Uncovering Sub‐Group Heterogeneity in Patients Coded for Group 3 Pulmonary Hypertension

Green R^1^, Arora S^1^, Randhawa T^1^, Dong A^1^, Sanmarco C^1^, Penugonda S^1^, Venker B^1^, Saggar R^2^

^1^Pulmovant, Waltham, USA, ^2^Division of Pulmonology, UCLA Health System, Los Angeles, USA

**Background:** WHO Group 3 pulmonary hypertension (PH) due to lung disease, identified by ICD‐10 code I27.23, comprises a diverse set of underlying lung conditions. New 2024 guidelines at the 7th World PH Symposium separate Group 3 into distinct clinical diagnosis subgroups associated with lung disease and/or hypoxia. The absence of specific ICD‐10 subgroup codes hampers patient identification and research efforts.

**Objective:** To quantify the composition of PH disease entities coded as I27.23 through manual review of electronic health records (EHR).

**Methods:** A retrospective observational study was conducted using data derived from the Loopback Platform, a multisource EHR‐based real‐world database of US patients containing de‐identified patient notes including labs and vitals. 118 patients of 516 were randomly selected from eight organizations, each with at least two I27.23 codes recorded 30 days apart. Two independent reviewers classified PH etiology by manually reviewing the available clinical notes. Inhaled treprostinil usage, the only approved medication for PH‐ILD, was also extracted.

**Results:** Among the 118 patients with the I27.23 code, 45 (38%) had PH due to chronic obstructive pulmonary disease (PH‐COPD), 28 (24%) had PH due to interstitial lung disease (PH‐ILD), 17 (14%) had PH due to connective tissue disease, 10 (9%) had PH due to obstructive sleep apnea (PH‐OSA), 4 (3%) had PH due to combined pulmonary fibrosis and emphysema (PH‐CPFE), 8 (7%) had an alternative type of PH, and 6 (5%) had no evidence of PH. Of the 45 non‐transplanted PH‐ILD patients, 33 (73%) were initiated on inhaled treprostinil, but only 22 (66%) continued treatment, the remaining 11 (34%) discontinued due to intolerance or worsened symptoms.

**Conclusion:** The heterogeneity within the I27.23 code underscores the need for more precise ICD‐10 coding to accurately identify and study subgroups of Group 3 PH. Improved classification may enhance patient care and facilitate targeted research efforts.

## 131. Circulating EC‐SOD Attenuates Inflammation and Hemodynamic Changes in Pulmonary Hypertension

Colon Hidalgo D^1^, Dee N^1^, Burciaga S^1^, Sul C^1^, Lewis C^1^, Jordan M^1^, Posey J^1^, Nozik E^1^

^1^University Of Colorado, Aurora, USA

**Introduction:** Pulmonary hypertension (PH) is a progressive condition that is characterized by inflammatory cell infiltration of the pulmonary vasculature and metabolic alteration of resident and circulating cells. Reactive oxygen species (ROS) play a key role in both processes and have been previously associated with PH pathogenesis. Extracellular superoxide dismutase (EC‐SOD), a major ROS scavenger, regulates oxidative stress, and its role in PH though reported remains not fully elucidates. In this study, we aimed to investigate the effect of EC‐SOD localization, specifically is re‐location from the vasculature to the circulation, on the development of pulmonary hypertension.

**Methods:** Wildtype mice and mice harboring the R213G EC‐SOD SNP were exposed to the Sugen hypoxia (SuHx) model. Hemodynamics were measured via the open chest method. Right ventricular weights were assessed via right ventricular weight (Fulton index). Additionally, vascular remodeling was evaluated via immunohistochemistry, while macrophage infiltration in lung tissues was quantified using flow cytometry.

**Results:** Mice carrying the R213G SNP were protected from the hemodynamic changes associated with PH as these mice had lower mean pulmonary artery pressures (mPAP) when compared to WT mice. Furthermore, protection was observed in terms of decreased vascular remodeling, as well as reduced cellular proliferation within the pulmonary vasculature. Macrophage infiltration and circulating levels of the pro‐inflammatory cytokine interleukin‐1 (IL‐1) were also diminished in R213G SNP mice, suggesting an attenuation of inflammation in this genetic model.

**Conclusion:** Our findings demonstrate that the R213G SNP in EC‐SOD confers protection against SuHx‐induced pulmonary hypertension. This protection may be mediated through reduced oxidative stress and inflammatory responses, highlighting a potential therapeutic target for modulating PH progression.

## 132. Atrial Flow Regulator (AFR®) ‐Device As “Bridge to Lung‐Transplant” in Patients With Severe Pulmonary Arterial Hypertension

Pattathu J^1^, Dold S^1^, Dalla Pozza R^1^, Jakob A^1^, Michel S^1^, Apitz C^2^, Griese M^1^, Haas N^1^

^1^LMU Universitiy Munich, Germany, Munich, Germany, ^2^University Hospital Ulm, Ulm, Germany

**Background:** Pulmonary arterial hypertension (PAH) is a rare, progressive disease that frequently leads to right heart failure, often necessitating lung transplantation. However, limited donor availability leads to patient deterioration or death while awaiting transplantation. Advanced interventional strategies are required to bridge this gap.

**Management Strategies:** In PAH, low cardiac output results from restricted transpulmonary blood flow rather than elevated pulmonary arterial pressure. Syncope, a severe symptom of PAH, is caused by acute reductions in transpulmonary blood flow. An intra‐atrial shunt, created with an AFR® device (Occlutech, Germany), decompresses the right atrium and sustains cardiac output while controlling desaturation with restricted blood flow.

**Cases:** Three patients with severe PAH (a 7‐year‐old girl, a 37‐year‐old male, and a 39‐year‐old female) were treated with AFR® devices. The girl and the male presented with recurrent syncope, while the female exhibited right ventricular decompensation and renal failure. Cardiac catheterisation revealed reduced cardiac output in all cases. Despite targeted pharmacological therapy, their conditions worsened, necessitating listing for lung transplantation. AFR® devices (8 mm, 10 mm, and 4 mm) were implanted, leading to improved cardiac output, renal function, and clinical status without further syncopal episodes. All patients underwent successful bilateral lung transplantation within three months.

**Discussion:** The AFR® device effectively maintained cardiac output in patients with right heart failure or syncope. Proper device sizing (4, 8, and 10 mm) was essential for favourable outcomes.

**Conclusion:** The AFR® device offers a viable interventional strategy to stabilise PAH patients, ensuring cardiac output and serving as a bridge to lung transplantation during right heart failure or pulmonary hypertension crises.

## 133. Comorbidity Burden Among Pulmonary Arterial Hypertension (PAH) Patients in the United States

Black H^1^, Chakinala M^2^, Watzker A^1^, Weiss T^1^, Martinez E^1^, Yang X^3^, Tate A^3^, Mandinov L^3^, Young S^3^, Were J^3^, Lautsch D^1^

^1^MSD, Rahway, United States, ^2^Washington University, St Louis, United States, ^3^Parexel International, Dublin, Ireland

Comorbidities are an important consideration when treating patients with PAH. Given existing ERS guidelines, emerging data, and clinical debates regarding use of combination treatment (tx) at diagnosis, we describe comorbidity burden and level of tx in the United States (US).

This retrospective cohort study used electronic medical record data between 2014‐2023 from TriNetX. Included patients had evidence of right heart catheterization and PAH diagnosis prior to tx. Patients were grouped by number of cardiopulmonary comorbidities (defined by ERS guidelines) during the 12‐month baseline and stratified by level of tx.

Overall comorbidity burden of the cohort was high (n = 874); 47% had a Charlson Comorbidity Index score ≥ 5, suggesting high likelihood of mortality. Within 30 days after index, 68% were on monotherapy compared to dual (25%) or triple (7%) tx. Most common cardiopulmonary comorbidities were hypertension (44%), diabetes (34%), obesity (29%) and coronary heart disease (27%). Other frequent comorbidities were COPD (49%), hyperlipidemia (38%), and sleep apnea (38%).

Grouped by number of cardiopulmonary comorbidities, 25% had 3‐4, 37% had 1‐2, and 38% had 0. Patients with ≥ 3 cardiopulmonary comorbidities compared to 1‐2, or none, were: older (mean age 63 vs 61 [1‐2] vs 53 [0]), fewer female (54% vs 64% vs 68%), higher BMI (mean (SD) 35 (8.69) vs 30 (8.05) vs 27 (6.47)), with history of tobacco use (62% vs 43% vs 33%). They had shorter time from PAH diagnosis to tx initiation (median of 15 vs 23 [1‐2] vs 49 [0] days; overall range of 5‐150) and were most frequently on monotherapy (72% vs 69% [1‐2] vs 63% [0]).

PAH patients in the US experience a high comorbidity burden. Those with higher burden are more likely to be prescribed monotherapy than patients with lower burden. This data can inform clinical discussions regarding use of combination tx in patients with comorbidities.

## 134. Immunometabolism of Macrophages in Pulmonary Arterial Hypertension (Pah): Relevance to Right Ventricular Failure

Jefferson L^1^, Lima P^1,2^, Martin A^1^, Bentley R^1^, Wilkinson M^1^, Dunham‐Snary K^1,3^, Dasgupta A^1^, Archer S^1,2^

^1^Department of Medicine, Queen's University, Kingston, Canada, ^2^Translational Institute of Medicine (TIME), Queen's University, Kingston, Canada, ^3^Department of Biomedical and Molecular Sciences, Queen's University, Kingston, Canada

**Background:** Pulmonary arterial hypertension (PAH) is an obliterative pulmonary vasculopathy, often resulting in RV failure. In males, inflammatory RV‐macrophages aid in mediating decompensated RV remodelling and failure. Preliminary data suggest that metabolic programming of RV‐macrophages, notably suppression of mitochondrial oxidative function and increased uncoupled glycolysis, is associated with inflammatory macrophages.

**Hypothesis:** RV dysfunction in male PAH rats is exacerbated by uncoupling of glycolysis from glucose oxidation in RV‐macrophages, associated with an inflammatory phenotype.

**Aim:** Determine the relationship between abnormal glucose metabolism and inflammatory macrophage phenotype in MCT‐PAH.

**Methods:** PAH was induced in male rats using monocrotaline (MCT; 60 mg/Kg) and confirmed via echocardiography (n = 6/group). To study macrophage physiology, we differentiated bone marrow‐derived macrophages (BMDM) in vitro, as isolating RV‐macrophages in rats is not currently possible. Flow cytometry confirmed cell identity (> 95% CD45 + CD68 + CD11b+cells) and phenotype (His48+ vs. His48‐). We used Seahorse micropolarimetry to compare oxygen consumption in BMDM, while measuring NLPR3, pyruvate dehydrogenase kinase 1 (PDK1; inhibitor of glucose oxidation) via western blot. Expression of PDK1 was measured in RV‐macrophages by immunofluorescence.

**Results:** Basal respiration, maximal respiration, spare respiratory capacity, and ATP production trended to be lower in MCT BMDM compared to controls, although sample size precluded achieving statistical significance. MCT BMDM trended towards increased expression of NLRP3 and PDK1, and a higher incidence of CD68+His48+ activated macrophages (p=NS). Quantification of PDK1 in RV sections confirmed higher expression in MCT rat RV‐macrophages vs. controls (p < 0.0001).

**Conclusion:** MCT BMDMs trend towards an inflammatory phenotype and impaired glucose metabolism, associated with increased PDK1 expression in RV‐macrophages. A larger sample size is required to confirm findings suggesting a link between uncoupled glycolysis and the inflammatory phenotype of macrophages in PAH.

## 135. Loxl2 Inhibition Ameliorates Pulmonary Artery Remodeling in Pulmonary Hypertension

Steppan J^1^, Wang H^2^, Nandakumar K^1^, Gadkari M^4^, Poe A^2^, Pak L^3^, Brady T^2^, Berkowitz D^1^, Shimoda L^5^, Santhanam L^1^

^1^Department of Anesthesiology and Critical Care Medicine, School of Medicine, Johns Hopkins University, Baltimore, United States, ^2^Department of Biomedical Engineering, School of Medicine, Johns Hopkins University, Baltimore, United States, ^3^Department of Molecular and Cellular Biology, Krieger School of Arts and Sciences, Johns Hopkins University, Baltimore, United States, ^4^Department of Chemical and Biomolecular Engineering, Whiting School of Engineering, Johns Hopkins University, Baltimore, United States, ^5^Division of Pulmonary and Critical Care Medicine, School of Medicine, Johns Hopkins University, Baltimore, United States

Conduit pulmonary arterial stiffening and the resultant increase in pulmonary vascular impedance have emerged as an important underlying driver of pulmonary arterial hypertension (PAH). Given that matrix deposition is central to vascular remodeling, we evaluated the role of the collagen cross‐linking enzyme lysyl oxidase like 2 (LOXL2) in this study. Human pulmonary artery smooth muscle cells (PASMCs) subjected to hypoxia showed increased LOXL2 secretion. LOXL2 activity and expression were markedly higher in primary PASMCs isolated from the pulmonary arteries of the rat Sugen 5416 + hypoxia (SuHx) model of severe pulmonary hypertension (PH). Similarly, LOXL2 protein and mRNA levels were increased in the pulmonary arteries (PA) and lungs of rats with PH (SuHx and monocrota line (MCT) models). Pulmonary arteries (PAs) isolated from the rats with PH exhibted hypercontractility to phenylephrine and attenuated vasorelaxation elicited by acetylcholine, indicating severe endothelial dysfunction. Tensile testing revealed a significant increase in PA stiffness in PH. Treatment with PAT‐1251, a novel small‐molecule LOXL2 inhibitor, improved active and passive properties of the PA ex vivo. There was an improvement in right heart function as measured by right ventricular pressure volume loops in vivo with PAT‐1251. Importantly, PAT‐1251 treatment ameliorated PH, resulting in improved pulmonary artery pressures, right ventricular remodeling, and survival. Hypoxia‐induced LOXL2 activation is a causal mechanism in pulmonary artery stiffening in PH and pulmonary artery mechanical and functional decline. LOXL2 inhibition with PAT‐1251 could be a promising approach to improve pulmonary artery pressures, right ventricular elastance, cardiac relaxation, and survival in PAH.

## 136. Evaluating the Relationship Between Ferritin, Transferrin, and Four‐Strata Risk Assessment in Pulmonary Arterial Hypertension Patients

Tilea I^1,2^, Cristescu L^1^, Iancu D^1^, Varga A^1,2^

^1^“George Emil Palade” University Of Medicine, Pharmacy, Science And Technology, Targu Mures, Romania, ^2^Department of Internal Medicine II‐Cardiology, Pulmonary Hypertension Center, Emergency Clinical County Hospital, Targu Mures, Romania

**Background:** Pulmonary arterial hypertension (PAH) is a progressive condition that requires precise risk stratification for optimal management. The Four‐Strata Risk Assessment (FSRA) model is frequently used to evaluate disease severity and predict patient outcomes. This study focuses on the relationship between iron metabolism biomarkers (ferritin, transferrin) and FSRA scores in PAH patients, aiming to identify potential biomarkers for risk stratification.

**Methods:** A retrospective analysis was conducted on a cohort of 27 PAH patients. The mean age was 48.78  ±  20.24 years, and the cohort was predominantly female (59.25%). Ferritin levels were measured (median:39.0 µg/L; IQR:15.9–58.2; normal range:20–250 µg/L), as well as transferrin levels (mean:2.98 ± 0.54 g/L; normal range:2–3.6 g/L). FSRA scores were assessed at two time points: T0 (one year of stable therapy) and T1 (follow‐up, 12 months later). Pearson correlation and regression analyses were utilized to evaluate the relationships between these biomarkers and FSRA scores.

**Results:** Ferritin exhibited a weak positive correlation with FSRA scores at both time intervals, with correlation coefficients of r = 0.03 at baseline and r = 0.06 at follow‐up. These results indicate that ferritin levels are not strongly associated with the severity of risk stratification in this PAH cohort. Similarly, transferrin showed no significant correlations with FSRA scores, suggesting its limited prognostic value in this population. Intra‐class correlation coefficient analysis revealed significant variability of FSRA scores between subjects (F(26,26) = 20.77, p < 0.001), while temporal variability was not significant (F(1,26) = 1, p > 0.33), confirming the stability of FSRA measurements between T0 and T1.

**Conclusion:** Neither ferritin nor transferrin demonstrated meaningful correlations with FSRA scores in PAH patients. Although ferritin is commonly associated with disease progression, its reliability as a biomarker for risk stratification in PAH is uncertain. Given the limited number of patients and the single‐center nature of this study, further research is needed to investigate alternative biomarkers that may provide more accurate risk assessment in this population.

## 137. Phvoicetrack – A Novel App For Voice Analysis in Pulmonary Hypertension

Igras‐Cybulska M^1^, Cybulski A^1^, Majdak M^2^, Doboszynska A^3^, Swietlik EM^3,4^

^1^Interactive Crafts sp. z o.o., Poland, ^2^Polish Academy of Sciences, Warsaw, Poland, ^3^University of Warmia and Mazury, Olsztyn, Poland, ^4^Cambridge University Hospitals NHS Foundation Trust, Cambridge, UK

Pulmonary hypertension (PH) is a progressive and life‐threatening condition characterised by elevated pulmonary arterial pressure, leading to right heart failure. Early diagnosis and ongoing monitoring are crucial for improving patient outcomes. PHVoiceTrack is a novel mobile application designed to collect, record, and analyse the voices of PH patients, offering a unique and complex approach to monitoring the disease by correlating voice data with clinical parameters.

The app captures voice samples, both within standardised protocol and spontaneous speech, alongside wearable device data, such as physical activity, heart rate, blood pressure, oxygen saturation, and other sensors (e.g., carbon dioxide or air pollution). Additionally, it allows patients to input information on symptoms, mood, medications, and hospital admissions. By integrating these diverse data streams, PHVoiceTrack aims to provide patients, their families, and healthcare professionals with comprehensive insights into patient health, supporting both diagnosis and ongoing disease management. The app's accessibility, encompassing usability and support for the most popular mobile systems and multilingual capability will ensure accessibility to diverse populations. It will be widely distributed through national PH patient associations, enabling global adoption.

The core objective of PHVoiceTrack is to build a robust corpus of anonymised voice samples, creating a valuable dataset for advanced analysis. This dataset will allow us to conduct voice, sentiment, emotional, linguistic, and semantic analyses to explore how changes in voice patterns may reflect disease progression, emotional well‐being, and treatment response. Ultimately, the goal is to develop reliable voice biomarkers that can be used for diagnosis, monitoring, and prognostication in PAH, offering a non‐invasive and continuous method for tracking patient health.

PHVoiceTrack represents a significant step forward in the use of digital health tools to support the management of pulmonary hypertension, with the potential to enhance both patient outcomes and clinical decision‐making through innovative, data‐driven insights.

## 138. Patient Voices: Voice Analysis in Patients With Pulmonary Arterial Hypertension

Swietlik EM^1,2^, Igras‐Cybulska M^3^, Majdak M^4^, Cybulski A^3^, Fay M^5^, Morrell N^6^

^1^Cambridge University Hospitals NHS Foundation Trust, Cambridge, UK, ^2^University of Warmia and Mazury, Olsztyn, Poland, ^3^Interactive Crafts sp. z o.o., Lublin, Poland, ^4^Polish Academy of Sciences, Warsaw, Poland, ^5^MF Research Consultancy, Newcastle, UK, ^6^University of Cambridge, Cambridge, UK

**Background:** Early diagnosis, timely treatment, and monitoring of Pulmonary Arterial Hypertension (PAH) are vital, but current methods are invasive and costly. This study investigates voice analysis as a non‐invasive alternative by identifying significant acoustic differences between PAH patients and healthy controls using speech recordings.

**Methods:** Thirty‐one PAH patients and 18 controls were interviewed about genetic research, disease experience, and family dynamics. One‐minute monologues of voice recordings were analysed using Audacity, and openSMILE, which extracted 6,373 voice biomarkers. Comparisons between groups were performed using appropriate statistical tests according to data distribution, and Holm‐Bonferroni correction was applied to control for false positives. A Random Forest classifier was employed for classification. Perceptual analysis using the GRBAS scale and sentiment/linguistic analysis of transcripts were conducted.

**Results:** After Holm‐Bonferroni correction, 1,002 features remained significant. Spectral parameters, such as the spectral centroid and MFCCs, showed significant differences. For example, mfcc_sma[7]_lpc2 and pcm_zcr_sma_stddev were higher in patients, suggesting altered spectral characteristics and increased variability in vocal cords vibration. Features related to the spectral envelope, like audSpec_Rfilt_sma[23]_percentile1.0, were also elevated, indicating changes in vocal timbre or articulation. Conversely, temporal dynamics features, like audSpec_Rfilt_sma_de[25]_risetime, were lower in patients, suggesting slower speech modulation. The Random Forest classifier achieved 96% accuracy and a 0.96 F1‐score, misclassifying only two patients, indicating strong performance. These results suggest the diagnostic potential of acoustic features.

Perceptual analysis showed normal articulation and vocal clarity, with minor issues like occasional breathiness or strain during fatigue or stress. Reduced vocal amplitude and slight tension in lower tones were noted, likely due to exhaustion. Linguistic analysis indicated positive attitudes toward genetic research and family dynamics.

**Conclusions:** These findings highlight the potential of acoustic features to distinguish patients from controls, offering valuable insights for diagnostic and future prognostic applications.

## 139. Severe Persistent Pulmonary Hypertension of the Newborn Associated With Incontinentia Pigmenti. Transient Course With Late Recurrence

Polak K^1^, Segura De la Cal T^2^, Labrandero de Lera C^3^, Álvarez M^1^, Garrido Lestache E^1^, Pantoja A^4^, Domínguez O^4^, Íñigo G^4^, Del Cerro M^1^

^1^Pediatric PH Unit, Cardiology Department, Ramón y Cajal, University Hospital., Madrid, Spain, ^2^Adult Pulmonary hypertension Unit. Hospital 12 de Octubre., Madrid, Spain, ^3^Pediatric Cardiology, La Paz University Hospital., Madrid, Spain, ^4^Pediatrics Department, Virgen de la Salud Hospital., Toledo, Spain

**Introduction:** Pulmonary arterial hypertension is an uncommon complication of incontinentia pigmenti (IP). In pediatrics, only a few cases had been reported, either with fatal outcome in infancy or with reversal of PH after months of aggressive targeted therapy. Nevertheless, this is the first pediatric case of life‐threatening PPHN, initially considered transient, but with late recurrence of PAH 9 years after the interruption of PH targeted therapy.

**Clinical Case:** A 29‐year‐old female diagnosed with incontinentia pigmenti (IP), with documented mutation in the NEMO/IKBG gene (deletion in exons 4‐10) gave birth to a full‐term baby girl. On the 4th postnatal day, the baby developed blister lesions with a linear distribution on the trunk, arms, legs, and fingers, which became hyperkeratotic in the following days. Viral cultures of the skin lesions were negative, and the skin biopsy confirmed the diagnosis of incontinentia pigmenti.

On postnatal day 8, she presented with severe PPHN, treated with inotropes, mechanical ventilatory support, nitric oxide, prostaglandins, sildenafil, and bosentan. She was finally discharged from the NICU after 26 days and home on the 45th postnatal day. Over the next months, the echocardiogram showed progressive normalization of pulmonary pressures, allowing withdrawal of PAH‐specific therapy: bosentan at 7 months and sildenafil 13 months later. The girl showed normal neurodevelopment and sporadic skin lesions. Follow‐up echocardiograms showed no signs of PH until age 9. CT scan revealed a diffuse mosaic pattern. Cardiac catheterization confirmed PAP/systemic PAP of 70%, mPAP 34‐41 mmHg, PVR 5.5 WU.m2, PVR/SVR 0.5, no response to vasoreactivity testing, and significant vascular remodeling in wedge angiographies. Asymptomatic and in functional class I, the patient was started on combined oral therapy (macitentan and tadalafil).

**Conclusion:** This case emphasizes the need for lifelong follow‐up of IP patients diagnosed with PH in infancy, despite apparent reversal with targeted therapies.

## 140. Hemodynamics Parameters and Oxygen Transport Changes in Patients With Idiopathic Pulmonary Arterial Hypertension Depending on Their Survival

Botsiuk Y^1^, Sirenko Y^2^

^1^Strazhesko Cardiology Institute, ^2^P.L. Shupyk National medical academy of postgraduate education,

Idiopathic pulmonary arterial hypertension (IPAH) ‐ is a serious and progressive disease that affects the vessels of the small blood circulation and leads to right ventricular failure and death. Indicators of hemodynamics and oxygen transport play central role in the progression and development of the disease. Materials and methods. 124 patients with IPAH were included in the study. Also, patients were divided into two groups: alive patients (group 1) and those who died (group 2). All patients underwent general clinical examinations, a 6‐minute walk test, transthoracic and speckle‐tracking echocardiography, right heart catheterization, blood gas analysis and calculation of hemodynamic parameters. Results. We calculated and compared in both groups of patients the right 19.93 ± 7.84 vs. 16.47 ± 4.67 g·m·m–2, p = 0.009 (RVSWI) and left 39.47 ± 10, 53 vs. 35.92 ± 11.19 g·m·m–2, p = 0.036 ventricular stroke work index (LVSWI) and their ratio 0.59 ± 0.16 vs. 0.46 ± 0.13, p = 0.008 (RVSWI/LVSWI), right 97 ± 2.16 vs. 1.82 ± 1.04 g·m, p = 0.001 (RVPC) and left ventricular pump coefficient 5.36 ± 4.84 vs. 4.41 ± 2.15 g·m, p = 0.04 (LVPC) and their ratio 0.54 ± 0.19 versus 0.44 ± 0.11, p = 0.05 (RVPC/LVPC). Also, we found differences in CO2 partial pressure indicators of 34.5 ± 5.1 vs. 40.2 ± 6.9 mm Hg, p = 0.02 (PaCO2) of venous blood, partial pressure of O2 90.2 ± 15.1 vs. 78.9 ± 11.2 mm Hg. p = 0.01 (PaO2) of arterial blood, arterial‐venous difference (A‐VO2) of oxygen content 4.5 ± 1.2 vs. 5.0 ± 0.8 ml/dL, p = 0.02 between both groups. Conclutions. RVSWI, LVSWI, RVSWI/LVSWI, RVPC, LVPC, RVPC/LVPC were significantly different between groups. The use of those hemodynamic parameters can help in a deeper understanding of the prognosis in IPAH patients. Performance of oxygen transport indicators (PaCO2, PaO2) and A‐VO2 in IPAH patients provides a broader understanding of the status and prognosis of this disease.

## 141. Patients With Group‐2 PH in a Low‐Resource Setting: Current Status

Kabwe J^1^, Kabwe L^1^, Mutema C^1^

^1^National Heart Hospital, Chongwe, Zambia

**Background:** Pulmonary hypertension (PH) due to left heart disease (LHD) is the most common type of PH. There are 2 subgroups of PH due to LHD, isolated postcapillary PH and combined post‐ and pre‐capillary PH. In low‐resource settings, diagnosing PH using the gold standard of right heart catheterisation with Swan Ganz catheter is a major challenge. National Heart Hospital is the only public cardiac centre for over 20 million people in Zambia.

**Methods:** RHC was performed in the Cath lab using the femoral approach guided by fluoroscopy. Fick's method was used to calculate cardiac output. We present an audit for 33 adult patients with PH‐LHD done at our facility in 2024.

**Results:** The average age was 39.33 with a gender distribution of males (15) and females (18). Differentiation into the two subgroups was IpcPH (20), CpcPH(11) and unclassified (2). The main pathologies were rheumatic valvular lesions (24), GUCH (5), and others (4). Of these patients, 14 have undergone successful open heart surgeries (OHS), while 7 are very high risk and thus inoperable in our settings and 10 are being medically managed in the cardiology unit.

**Discussion:** Due to the limited resources patients who undergo RHC are triaged only those with very high RVSP pressures on echo qualify. Some patients present to the hospital quite late posing a very high perioperative risk. As a new facility in low‐resource settings without access to ECMO or IABP or other LV assist devices, we implemented a local cut‐off point for OHS based on the outcome of some earlier patients we had operated on, to include only those with mPAP ≤ 40 mmHg and TAPSE ≥ 18 mm.

**Conclusion:** RHD remains the most common cause of PH‐LHD. Diagnostic and treatment challenges, late patient presentation and inadequate equipment for post‐operative care are the main drivers of PH‐LHD patient outcomes.

## 142. Decoding Right Ventricular Failure Through a Novel Preclinical PAH Model of Mitochondrial Dysfunction

Niihori M^1^, James J^1^, Kacar S^1^, Hinkle A^1^, Rafikov R^1^, Rafikova O^1^

^1^Indiana University, Indianapolis, United States

Pulmonary arterial hypertension (PAH) is a progressive and fatal disease with unmet clinical needs. Right ventricular (RV) dysfunction and failure are the major contributors to the mortality of PAH patients. However, current preclinical models inadequately replicate the pathobiology of RV dysfunction observed in patients, limiting our understanding of potentially targetable underlying mechanisms. Following the reports of the high prevalence of PAH in patients with a point mutation in the NFU1 protein, we developed a new rat model that reproduces the NFU1 mutation and severe mitochondrial dysfunction observed in human carriers. NFU1G206C rats spontaneously develop a PAH characterized by elevated RV systolic pressure, RV hypertrophy, and severe obliterative disease of small pulmonary arteries. The analysis of RV function revealed that RV preload in NFU1G206C rats remained unchanged, while afterload was significantly increased, replicating the Group 1 PAH phenotype. We also observed a substantial decrease in RV ejection fraction, stroke volume, cardiac output, and RV‐PA uncoupling. The single‐cell RNA sequencing of RV cardiomyocytes (CMs) isolated from NFU1G206C vs. WT rats discovered 35 differentially expressed genes (DEGs, fold change ≥ 5) that were identified as transcriptional factors or signaling pathways regulating angiogenesis or responsible for cytoskeletal reorganization, activation of proteasome degradation, metabolic adaptation, and antioxidant defense. While all these genes reflected compensatory mechanisms to pressure overload, the only factors identified as potentially maladaptive were negative cell cycle regulators. The enhanced CM arrest in G2 is a recognized contributor to cardiac hypertrophy, although the mechanisms that cause CM withdrawal from the cell cycle remain unknown. The emerging growth arrest factors highly expressed in dysfunctional RV can represent a novel mechanism involved in RV hypertrophy and maladaptation. We conclude that the NFU1G206C model of spontaneous PAH provides novel opportunities for investigating the mechanisms of RV dysfunction, such as molecular players regulating CM cell cycle arrest.

## 143. Automated Detection and Radiomic Characterization of Clots in Acute Pulmonary Embolism and CTEPH Using Nnunet

San Jose Estepar R^1^, San Jose Estepar R^1^, Nardelli P^1^, Mylvaganam R^2^, Schimmel D^2^, Chiu S^3^, Malaisrie S^3^, Cuttica M^2^, Rahaghi F^4^

^1^Dept. of Radiology, Brigham And Women's Hospital, Boston, USA, ^2^Dept. of Medicine, Northwestern University Feinberg School of Medicine, Chicago, USA, ^3^Dept. of Surgery, Northwestern University Feinberg School of Medicine, Chicago, USA, ^4^Dept. of Medicine, Brigham And Women's Hospital, Boston, USA

**Background:** Pulmonary embolism (PE) and chronic thromboembolic pulmonary hypertension (CTEPH) are conditions characterized by the presence of intraluminal clots, which may vary in size, density, and morphology. The detection and characterization of these clots can be challenging. We developed a nnUNet to automatically detect and segment potential clots in computed tomography pulmonary angiography (CTPA) images. Our objective was to evaluate this tool in a cohort of patients, including controls, acute PE, and CTEPH cases, and to explore radiomic differences in clot characteristics between these groups.

**Methods:** The nnUNet was trained using two public databases of 126 PE cases and was tested on a single‐institution cohort of 414 subjects: 177 controls, 88 CTEPH cases, and 149 acute PE cases. Clot detection and segmentation were performed, and radiomic features such as clot volume, mass, density, and density heterogeneity were extracted. Statistical comparisons between groups were performed using ANOVA, followed by post‐hoc Tukey's HSD tests.

**Results:** The nnUNet detected clots in 78% (323/414) of the cases: 111 controls (67%), 66 CTEPH (75%), and 146 acute PE (98%). Among the cases with detected clots, acute PE cases demonstrated significantly higher mean clot volume (11.0 mL vs. 0.7 mL in controls and 2.4 mL in CTEPH), higher clot mass (11.9 g vs. 0.8 g and 2.6 g), lower mean clot density (106.6 HU vs. 139.0 HU and 138.2 HU), and greater clot density heterogeneity (98.4 HU vs. 75.3 HU and 82.8 HU). All comparisons between acute PE and the other groups were statistically significant (p < 0.05) except for the clot heterogeneity comparison between acute PE and CTEPH.

**Conclusion:** Our method demonstrated high sensitivity for clot detection across different groups. Clot radiomics revealed distinct differences in detected clot characteristics between groups. These findings support the potential for advanced radiomic analysis to improve diagnostic stratification.

## 144. Mitochondrial Trifunctional Protein HADHA Is Critical in Regulating Mitochondrial Cardiolipin and Bioenergetics in Right Ventricle in Pulmonary Hypertension

Zhang P^1,2^, Zimmer A^1^, Tran K^1^, Wang E^1^, Clements R^1,3^, Song W^1,2,4^, Choudhary G^1,2,4^

^1^VA Providence Heathcare System, ^2^Department of Medicine, Alpert Medical School of Brown University, ^3^University of Rhode Island, ^4^Cardiovascular Research Center, Brown University Health,

**Background:** Pulmonary hypertension (PH) is prevalent in cardiopulmonary diseases. Unmet energy needs due to mitochondrial (mito) dysfunction results in myocardial contractile failure. Cardiolipin (CL) is critical for mito structure and cristae organization, which are essential for mito function and bioenergetics. Mito trifunctional protein, HADHA, regulates CL maturation. However, little is known about the changes in mature CL and its regulation by HADHA in RV in PH. We hypothesized that deregulated HADHA reduces CL maturation that leads to mito dysfunction and energy deficiency in RV in PH.

**Methods:** Both Sugen/hypoxia‐induced PH model (SuHx) and pulmonary artery banding mode (PAB) using adult Sprague‐Dawley rats were used. Hemodynamic and echocardiography were performed, followed by mito bioenergetics assessments using high‐resolution respirometry, mito morphology using electron microcopy, and HADHA and CL analysis. Mechanistic studies were also performed using in vitro cell culture models.

**Results:** Both SuHx and PAB models show RV systolic and diastolic dysfunction and significantly decreased mito oxidative phosphorylation. HADHA expression is reduced up to 50% in RV of both SuHx and PAB models in comparison to controls, which is associated with significant reduction in mature CL (CL[18:2]4) and less dense of cristae packing. Abundances of mito complex I, III, and IV in the supercomplex region are also decreased in RV of PH rats. Exposing H9c2 cells to hypoxia recapitulates the reduction of HADHA, oxidative phosphorylation, and mature CL observed in the dysfunctional RV which are attenuated by overexpression of HADHA. HADAH knockdown is sufficient to reduce oxidative phosphorylation, mature CL, mito cristae per area, assembly of supercomplexes, and increase inter‐cristae length in H9c2 cells.

**Conclusions:** HADHA determines CL maturation and regulates mito function. HADHA may be a novel therapeutic target to improve RV contractile function by enhancing mito function and bioenergetics in PH.

## 145. Cardiopulmonary Exercise Test for Risk Stratification in PAH and CTEPH Patients From Low‐Middle and High‐Income Countries

Santos‐Oliveira A^1^, Lichtblau M^2^, Paz‐Oliveira F^1^, Bastista‐Silva A^1^, Mesquita‐Silva L^1^, Cavolli J^1^, Ota‐Arakaki J^1^, Ulrich S^2^, Oliveira R^1^, Ferreira E^1^

^1^Federal University of Sao Paulo (Unifesp/EPM), São Paulo, Brazil, ^2^University of Zurich (UZ), Zurich, Switzerland

CPET variables are still undefined for risk stratification during follow‐up of PAH and CTEPH patients. Therefore, we investigated the prognostic relevance of CPET variables at baseline and during follow‐up for PAH and CTEPH patients from the PH Reference Center in low‐middle—and high‐income countries.

**Methods:** A derivation and validation cohort included retrospective PAH and CTEPH cases from Unifesp,Brazil(2007‐2023) and University of Zurich,Switzerland(2018‐2023). Enrolled patients: first CPET contemporary to RHC, in addition to NYHA, BNP/NTproBNP, and 6MWT, and a second test 4‐12 months after treatment. Variables were analyzed for event‐free‐survival (EFS: death or lung transplantation) and time to clinical worsening at one‐year (CW1y: all‐cause hospitalization, add‐on PH therapy, and EFS)—80% of the sample at random to create models and 20% to validate them.

**Results:** 345patients (73%Brazil) were evaluated, 65%PAH, 46±17years‐old and 74%female. 56% naïve‐treatment and 44%NYHA III/IV. Overall, 67%EFS and 12% had CW1y. Statistically significant variables in univariate analysis for EFS derivation cohort were: CPET variables at peak (V'O2, PETCO2, O2pulse,systolicBP) and submaximal (V'O2AT, ∆V'E/∆V'CO2, ∆V'O2/∆WR, ∆HR/∆V'O2), RHC (HR, CI, SV, SVi, SvO2), BNP/NTproBNP and 6MWT. In the multivarible model, VO2peak(%pred) [HR 0.976(0.961‐0.992),p = 0,002] and ∆V'E/∆V'CO2 [HR 1.020(1.007‐1.034),p = 0.003] explained E‐FS at follow up (p < 0.05). For CW1y, V'O2peak(p = 0.045) and peak systolicBP (p = 0.012) were in the final model. The additive model derived from the E‐FS final model showed a good result [AUC 0.74(0.60‐0.88)], while the logistic model to explain the CW1y was unable to provide a good prediction [AUC 0.697(0.38‐1.00)] in the validation sample.

**Conclusion:** Our results showed that CPET is relevant for PAH and CTEPH follow‐up to predict E‐FS for patients from low‐middle and high‐income countries. However, it did not add information for composite outcomes (CW1y); this may be explained for clinical decision‐making for hospitalization and add‐on therapy. Larger scale prospective studies for evaluating risk assessment strategies from different economies worldwide should be considered.

## 146. Pulmonary Vascular Pruning on CT Imaging Is Associated With Poorer Hemodynamics in Chronic Hypersensitivity Pneumonitis and Pulmonary Hypertension

Synn A^1^, Rahaghi F^2^, Nardelli P^2^, Pereira C^3^, Ota‐Arakaki J^3^, Verrastro C^3^, San José Estépar R^2^, Washko G^2^, San José Estépar R^2^, Oliveira R^3^

^1^Beth Israel Deaconess Medical Center, Boston, United States, ^2^Brigham and Women's Hospital, Boston, United States, ^3^Federal University of São Paulo, São Paulo, Brazil

**Background:** Chronic hypersensitivity pneumonitis (cHP) is a common ILD subtype that is associated with high morbidity and is increasing in prevalence. Like other ILDs, cHP may be complicated by pulmonary hypertension (PH), but this relationship remains poorly characterized. In this study, we sought to quantify the degree of vascular “pruning” (an image‐based metric of pulmonary vasculopathy) from chest CTs of patients with cHP‐PH and determine if pruning is associated with invasive hemodynamic measures.

**Methods:** We included 34 patients with cHP‐PH seen at the Federal University of São Paulo, Brazil, between 2011‐2013. From clinical CTs, quantitative image analysis algorithms were used to calculate the small vessel fraction (BV5/TBV), defined as small vessel (area< 5mm2) volume divided by total vessel volume. A lower BV5/TBV indicates more severe pruning. We examined BV5/TBV of the whole‐lung, and also for the lobe with lowest median density (i.e. least involved by ILD). We constructed linear regression models (unadjusted and adjusted for age/sex) to investigate associations of BV5/TBV and invasive hemodynamics on right heart catheterization, including mean pulmonary artery pressure (mPAP), pulmonary vascular resistance (PVR), PA compliance, and cardiac index.

**Results:** Whole‐lung BV5/TBV was not consistently associated with invasive hemodynamic measures. However, when examining lobe‐specific BV5/TBV, we found that CT‐based pruning was linked to poorer hemodynamics. For example, per standard deviation more severe pruning, mPAP was 2.1mmhg higher (95%CI: ‐0.1—4.3, p = 0.058) and PVR was 0.7WU higher (0.2—1.2, p = 0.008). Similarly, pruning was associated with lower PA compliance (p = 0.04) and lower cardiac index (p = 0.03). These relationships persisted after multivariable adjustment.

**Conclusion:** More severe vascular pruning on CT, especially in lobes least involved by ILD, is associated with more severe invasive pulmonary hemodynamics in patients with cHP‐PH. The degree of vascular pruning by CT may be a non‐invasive indicator of more severe pulmonary vascular disease in this vulnerable population.

## 147. Sustained Benefit With Seralutinib Treatment: A Post‐Hoc Analysis of The TORREY Open‐Label Extension

Benza R^1^, Hoffman S^2^, Sahay S^3^, Escribano Subías P^4^, Zolty R^5^, Kingrey J^6^, Penn B^7^, Sobol I^8^, Sood N^9^, Channick R^10^, Chin K^11^, Frantz R^12^, Ghofrani H^13^, Hemnes A^14^, Howard L^15^, McLaughlin V^16^, Vachiéry J^17^, Zamanian R^18^, Cravets M^2^, Osterhout R^2^, Roscigno R^2^, Parsley E^2^, Aranda R^2^, Zisman L^2^, Sitbon O^19^

^1^Mount Sinai Heart, Icahn School of Medicine at Mount Sinai, Mount Sinai Hospital, New York, USA, ^2^Gossamer Bio, Inc., San Diego, USA, ^3^Houston Methodist Hospital/Weill Cornell Medicine, Houston, USA, ^4^University Hospital 12 de Octubre, Complutense University, Madrid, Spain, ^5^University of Nebraska Medical Center, Omaha, USA, ^6^INTEGRIS Health Pulmonary Hypertension Center of Oklahoma, Oklahoma City, USA, ^7^University of Utah Health, Salt Lake City, USA, ^8^New York Presbyterian/Weill Cornell Medical Center, New York, USA, ^9^University of California Davis Medical Center, Sacramento, USA, ^10^University of California Los Angeles, UCLA Medical Center, Los Angeles, USA, ^11^UT Southwestern Medical Center, Dallas, USA, ^12^Mayo Clinic, Rochester, USA, ^13^Universities of Giessen and Marburg Lung Center (UGMLC), Institute for Lung Health (ILH); Cardio‐Pulmonary Institute (CPI); Member of the German Center for Lung Research (DZL), Giessen, Germany, ^14^Vanderbilt University, Vanderbilt University Medical Center, Nashville, USA, ^15^Imperial College Healthcare NHS Trust, Hammersmith Hospital, London, UK, ^16^University of Michigan, Ann Arbor, USA, ^17^Université Libre de Bruxelles, HUB – Hôpital Erasme, Brussels, Belgium, ^18^Stanford University School of Medicine, Stanford Medicine, Stanford, USA, ^19^Hôpital Bicêtre (AP‐HP), Université Paris‐Saclay, Le Kremlin‐Bicêtre, France

**Background:** A reduction in pulmonary vascular resistance (PVR) of 15‐50% in patients with pulmonary arterial hypertension (PAH) has been observed in studies of combination therapy. Seralutinib, a novel inhaled PDGFRα/ß, CSF1R, and c‐KIT kinase inhibitor, significantly reduced PVR at Week (W) 24 in the phase 2 TORREY study (NCT04456998). Most patients were WHO functional class (FC) II, receiving 3 PAH medications at randomization (baseline, BL), including prostacyclin. To characterize the longer‐term hemodynamic benefits of seralutinib, we identified patients in the OLE (NCT04816604) with a > 15% reduction in PVR from BL to W72.

**Methods:** 74 patients enrolled in the OLE. Hemodynamic data were available for 53 patients at W72; patients demonstrating a > 15% PVR reduction from BL to W72 were identified. Results are descriptive.

**Results:** Seralutinib treatment for up to 72 weeks resulted in a > 15% PVR decrease in 26 (49%) patients. The mean BL PVR was 643 dynes*s/cm⁵, six‐minute walk distance (6MWD) was 413 m, and 17 patients were WHO FC II. 13 patients were receiving 3 PAH medications, including prostacyclin. The mean PVR reduction was 234 dynes*s/cm⁵ (36%) at W72. Furthermore, mean pulmonary artery pressure decreased by 6.5 mmHg (13%), cardiac output increased by 0.9 L/min (20%), and 6MWD increased from BL by 40 m. Seralutinib was well tolerated.

**Conclusions:** In 49% of patients with W72 right heart catheterization data, longer‐term seralutinib treatment resulted in sustained hemodynamic and 6MWD improvement.

## 148. Sustained Effect of Seralutinib on Circulating Biomarkers in the TORREY Phase 2 Open‐Label Extension Study

Ghofrani H^1^, Osterhout R^2^, Hemnes A^3^, Sitbon O^4^, Benza R^5^, Channick R^6^, Chin K^7^, Frantz R^8^, Howard L^9^, McLaughlin V^10^, Vachiéry J^11^, Roscigno R^2^, Zisman L^2^, Aranda R^2^, Bruey J^2^, Zamanian R^12^

^1^Universities of Giessen and Marburg Lung Center (UGMLC), Institute for Lung Health (ILH); Cardio‐Pulmonary Institute (CPI); Member of the German Center for Lung Research (DZL), Giessen, Germany, ^2^Gossamer Bio, Inc., San Diego, USA, ^3^Vanderbilt University, Vanderbilt University Medical Center, Nashville, USA, ^4^Hôpital Bicêtre (AP‐HP), Université Paris‐Saclay, Le Kremlin‐Bicêtre, France, ^5^Mount Sinai Heart, Icahn School of Medicine at Mount Sinai, Mount Sinai Hospital, New York, USA, ^6^University of California Los Angeles, UCLA Medical Center, Los Angeles, USA, ^7^UT Southwestern Medical Center, Dallas, USA, ^8^Mayo Clinic, Rochester, USA, ^9^Imperial College Healthcare NHS Trust, Hammersmith Hospital, London, UK, ^10^University of Michigan, Ann Arbor, USA, ^11^Université Libre de Bruxelles, HUB – Hôpital Erasme, Brussels, Belgium, ^12^Stanford University School of Medicine, Stanford Medicine, Stanford, USA

**Background:** Seralutinib is an inhaled PDGFRα/β, CSF1R and c‐KIT kinase inhibitor targeting pathways driving vascular remodeling in pulmonary arterial hypertension (PAH). The TORREY phase 2 study met its primary endpoint, demonstrating a significant reduction in pulmonary vascular resistance in patients with PAH (NCT04456998). An exploratory analysis of TORREY identified 223 circulating biomarkers associated with seralutinib treatment relative to placebo at week 24. We present changes in these biomarkers following long‐term treatment in patients participating in an open‐label extension (NCT04816604).

**Methods:** Olink proteomics data were generated for 21 placebo‐to‐seralutinib and 22 continued seralutinib patients treated for 48 or 72 weeks, respectively. One‐sided Wilcoxon signed‐rank tests were used to assess biomarker changes over the treatment period.

**Results:** In the placebo‐to‐seralutinib arm, 87/223 (39%) of the previously identified protein changes were recapitulated at nominal significance (p < 0.05). In the continued seralutinib arm, 105/223 (47%) protein changes were maintained after long‐term treatment. Decreasing proteins include extracellular matrix (COL1A1, ITGB2, FAP), chemokines (CCL3,7) and RAS related proteins. Increasing proteins include anti‐inflammatory factors (IL10, SCGB3A2) and metalloproteinase regulators (TIMP4). The directionality of the observed changes, as in the TORREY study, was consistent with anti‐fibrotic, anti‐inflammatory, and anti‐proliferative effects.

**Conclusion:** The observed long‐term biomarker changes support a sustained effect of seralutinib on pathways relevant to PAH pathogenesis.

## 149. Pulmonary Hypertension in Pediatric Pulmonary Vein Stenosis

Mistry M^1^, Rogers H^1^, Callahan R^2^, Keochakian M^1^, Gauvreau K^1^, Jenkins K^1^

^1^Boston Children's Hospital, Boston, USA, ^2^Children's Hospital of Philadelphia, Philadelpia, USA

Pulmonary vein stenosis (PVS) is a rare, life‐threatening condition in infants and young children, characterized by neointimal proliferation leading to luminal obliteration. Despite interventions, patients often exhibit elevated mean pulmonary artery pressure (mPAP), which can impact clinical outcomes. This study assessed mPAP in patients undergoing multimodal treatment with surgery, catheterization and medical therapy for PVS, examining its relationship with survival.

Data from 60 patients with intraluminal PVS involving ≥ 2 vessels and normal biventricular circulation were analyzed. Patients were stratified by prematurity, lung disease, and congenital heart disease (CHD). The number of affected pulmonary veins (PVs), mPAP pre‐ and post‐intervention or prior to death or transplant, use of pulmonary vasodilators and survival were evaluated.

The median age at presentation was 10.8 months. Pre‐intervention mPAP averaged 37.5 mmHg ± 14.9 mmHg and 38.4 mmHg ± 12.8 mmHg post‐intervention. Among the cohort, 55‐percent were treated with imatinib, 38‐percent with sildenafil, 15‐percent with avastin and 2% with bosentan. Among CHD patients (n = 29), a median of three PVs were affected, pre‐intervention MPAP averaged 31.1 mmHg, and decreased by 2.3 mmHg. In those with lung disease and/or prematurity (n = 11), a median of four PVs were affected, pre‐mPAP averaged 63.7 mmHg, and decreased by 8.3 mmHg. In patients with both CHD and lung disease (n = 19), a median of three PVs were affected, pre‐mPAP averaged 41.5 mmHg, and decreased by 5.3 mmHg. Non‐survivors had higher pre‐intervention mPAP (41.1 mmHg) compared to survivors (32.2 mmHg) (p‐value = 0.051). Patients with lung disease had the most affected PVs, highest initial mPAP (63.7 mmHg), and highest mortality (64%).

This study highlights the importance of monitoring mPAPs as a prognostic marker and a therapeutic target in patients with severe PVS. Future research should focus on the biologic and physiologic determinants of mPAP elevation in patients with PVS to refine therapeutic intervention.

## 150. Sotatercept use in Pulmonary Arterial Hypertension: A Single Center Experience

Safdar Z^1^, Sahay S^1^, Chavarria M^1^, Villasmil Hernandez N^1^

^1^Houston Methodist Hospital Lung Center, Houston, United states

**Introduction:** Pulmonary arterial hypertension (PAH) is a progressive disease that requires escalation of therapies. Sotatercept, activin signaling inhibitor, was recently approved by FDA to treat PAH. We describe our experience in treating prevalent PAH patients with sotatercept that lead to improvement in hemodynamics allowing downtitration of PAH meds.

**Results:** Mean age was 56 ± 7 years, all were females. First patient with PAH associated with connective tissue disease (CTD) diagnosed at age 39 and receiving intravenous treprostinil (i.v Trep) had progressive worsening symptoms and i.v Trep was gradually uptitrated to 129 ng.kg.min. Sotatercept SQ injection (0.7 mg.kg) was started on April 2024, NT pro BNP levels decreased from 4046 to 935 pg.ml. Her i.v Trep dose was reduced to 109 ng.kg.min, pulmonary vascular resistance (PVR) improved from 14 WU to 5.7 WU. Second patient with CTD‐ILD on nintedanib, was diagnosed with PH‐ILD and started on inhaled Trep and sildenafil with subsequent worsening of symptoms. Repeat hemodynamics showed PVR of 27 WU and NT pro BNP of 19538 pg.ml. I.V. Trep was added and up‐titrated to 30 ng.kg.min with repeat PVR of 5 WU and NT pro BNP level of 1185 pg.ml. Sotatercept was added with further reduction in NT pro BNP levels to 324 pg/ml with WHO FC II symptoms. Third patient with PAH associated with methamphetamine abuse, had PVR of 16 WU and NT pro BNP level of 5939 pg.ml, started on macitentan and iv epoprostenol (i.v. Epo) that was titrated to 7.7 ng.kg.min. Sotatercept was added and repeat NT pro BNP level was 539 pg/ml and iv Epo dose is being down titrated. REVEAL risk score 2.0 at diagnosis was 10 and reduced to 2 after sotatercept.

**Conclusion:** Sotatercept can be used in advance PAH patients with improvement in hemodynamics and reduction in the need for PAH medications.

## 151. Loss of WWOX Triggers Proliferation in Human Pulmonary Arterial Smooth Muscle Cells in Response to PI3K/AKT/mTOR Activation

Gomes M^1^, Bai Y^1^, Smith P^1^, Saliba J^1^, Machado R^1^

^1^Indiana University, Indianapolis, United States

**Introduction:** Pulmonary arterial hypertension (PAH) is a severe pulmonary vascular disease that leads to structural changes in lung vessels, cardiac hypertrophy, and heart failure. Improvements in the prognosis of PAH patients require innovative therapeutic strategies.

Similar to cancer, the lungs vessels in PAH exhibit hyperproliferation cells, resistance to apoptosis, and metabolic reprogramming. These similarities led us to investigate the role of the tumor suppressor WW domain‐containing oxidoreductase (WWOX) in the development of PAH. WWOX is known not to be expressed in many types of cancer, affecting key cellular processes, such as proliferation, apoptosis, and metabolism. We hypothesize that the absence of WWOX contributes to cell hyperproliferation and resistance to apoptosis, which are characteristics of PAH.

**Methods and Results:** Our results showed that mRNA and protein levels of WWOX are decreased in pulmonary arterial smooth muscle cells (PASMC) from patients with PAH. Then, we reduced WWOX expression in human PASMC by using small interfering RNA. BrDU assay confirmed that hPASMC WWOX‐siRNA showed an increase in cell proliferation. Transwell migration assay illustrated an increase in PASMC WWOX‐siRNA migration. We also evaluated cell cycle phases (flow cytometry) that demonstrated an increase in the G2/M phase in hPASMC WWOX siRNA in comparison to scramble cells. Loss of WWOX promotes the upregulation of cyclin‐dependent kinases (CDKs), CDK2, CDK4, CDK6 causing dysfunction in mitotic checkpoints. We investigated the signal transduction that promotes cell growth, and cell cycle progression. Western blotting showed increases in phosphorylation of AKT, mTOR and S6 kinase in hPASMC WWOX siRNA, which suggests activation of PI3K pathway. The activation of AKT may be regulated by mTOR complex 1 (mTORC1), which initiates several cellular signals that promote cell proliferation.

**Conclusion:** Loss of WWOX promotes PI3K/AKT/mTOR activation which supports cell cycle progression and proliferation in hPASMCs. These effects may contribute to vascular dysfunction in PAH.

## 152. Correlation of Endothelial Progenitor Cells With Right Heart Failure Associated With Pulmonary Hypertension and Congenital Heart Disease in Paediatric Patients

Garcia H^1^

^1^Hospital Angeles Lomas, Mexico City, Mexico

Pulmonary arterial hypertension is a serious condition that causes right heart failure and short life expectancy. The pathophysiology of this pulmonary disease and right heart failure is associated with persistent inflammatory processes in vascular endothelial tissue, which may stimulate bone marrow (BM) responses to release endothelial progenitor or stem cells expressing CD34/CD133 immuno‐phenotype, possibly as a mechanism of response to vascular damage for vascular reconstruction.

**Methods:** We included patients with PAH‐CC seen in the Paediatric Cardiology service of the CMN 20 de Noviembre. A 2 ml blood sample was obtained. Using flow cytometry with MACSQuant Analyzer 10 equipment, CD34+/CD133+ cellularity was determined. Correlation analysis was performed with Spearman's Rho and multiple logistic regression. Differences between groups were determined with Student's t‐test. Statistical significance was determined with a p‐value of < 0.05.

**Results:** Our study demonstrated a significant correlation r 0.6 in relation to the presence of pulmonary hypertension and congenital heart disease and a stronger positive correlation between pulmonary hypertension in patients with congenital heart disease in the presence of heart failure r 0.7.

Based on our study, it is logical to think that the quantification of this type of cell subpopulations in patients with PAH‐CC could be used for the following clinical and research purposes: possible markers of the degree of evolution of pulmonary and cardiovascular vascular endothelial injury, thus stratifying cardiopulmonary disease. possible diagnostic marker of right heart failure even when the clinical and haemodynamic picture is incipient. and potential prognostic marker for the development of right heart failure.

## 153. Novel Biomarkers Identified by Proteomic Screening Improve Prognostic Risk Stratification in Human Pulmonary Arterial Hypertension

Kuwabara Y^1^, Yokokawa T^1,2^, Lemay S^1^, Sauvaget M^1^, Martineau S^1^, Breuils‐Bonnet S^1^, Potus F^1,3^, Bonnet S^1,3^, Provencher S^1,3^, Boucherat O^1,3^

^1^Pulmonary Hypertension Research Group, Quebec Heart And Lung Institute Research Centre, Quebec, Canada, ^2^Department of Cardiovascular Medicine, Fukushima Medical University, Fukushima, Japan, ^3^Department of Medicine, Université Laval, Quebec, Canada

**Introduction:** In recent years, significant progress has been made in the management of patients with pulmonary arterial hypertension (PAH). Nevertheless, the prognosis of PAH patients remains poor and difficult to predict. To date, BNP/NT‐proBNP are the only circulating biomarkers used as part of composite PAH risk assessment tools.

**Objective:** We investigated the possibility of identifying new biomarkers that could provide additional information on disease severity and prognosis using high‐throughput proteomics.

**Method:** More than 700 plasma proteins related to oncology and neurology were measured in 60 PAH patients and 28 age‐ and sex‐matched controls using Olink proximity extension assay. Cox proportional hazard analyses and C‐statistics were performed to assess the prognostic value and the incremental prognostic value of differentially expressed proteins. Expression levels of selected proteins were evaluated in the lung and right ventricle (RV) of PAH animal models using qPCR and Western blot. Human pulmonary artery smooth muscle cells (PASMC) and cardiac fibroblasts (CF) were stimulated with the selected protein, and immunofluorescence was performed to assess its effect on cell proliferation.

**Results:** One hundred and fourteen plasma proteins were up‐regulated in PAH patients compared to controls (fold change ≥ 1.5 & adjusted p‐value ≤ 0.05). Among them, 14 proteins, namely FGF23, KLK4, EDA2R, MDK, WFDC2, ATOX1, ADM, TNFRSF6B, THBS2, SFRP1, GGT1, CALCA, SPINK1 and TNFRSF10B remained independent prognostic factors after adjustment for the 2015 ESC/ERS, the REVEAL 2.0 risk scores and the refined 4‐strata risk assessment tool. EDA2R, TNFRSF10B, WFDC2 and CALCA added prognostic value on top of these predictive models. WFDC2 was significantly upregulated in the lung and RV of monocrotaline and Sugen/Hypoxia rats. Treatment with recombinant WFDC2 promoted proliferation of PASMCs and CFs (EdU incorporation).

**Conclusion:** Our proteomic screening identified promising circulating biomarkers to improve mortality risk stratification in PAH. WFDC2 may contribute to pathological remodeling in PAH.

## 154. Expanding Heart Transplant Eligibility in High‐Risk Cardiogenic Shock Patients: The Transformative Role of Impella 5.5 in Patients With Pulmonary Hypertension

Klingbeil R^1^, Desai A^1^, Shapiro A^1^, Ruiz J^1^, Martin A^1^, Goswami R^1^

^1^Mayo Clinic, Jacksonville, USA

**Background:** Elevated pulmonary vascular resistance (PVR) is a barrier to Heart Transplant (HT) candidacy in patients with advanced heart failure (HF) and pulmonary hypertension (PH). Traditionally, durable mechanical circulatory support (MCS) is required for months to achieve significant reductions in PVR. The Impella 5.5 offers a novel approach by providing rapid and sustained reductions in PVR. This study assessed PVR within 72 hours of Impella 5.5 placement and its sustained impact over the support period.

**Methods:** This retrospective cohort analysis included 74 patients who underwent Impella 5.5 implantation for advanced HF. Hemodynamic data, including baseline and 72‐hour post‐implantation PVR, were collected. The cohort was stratified based on baseline PVR: ≥ 4 WU and < 4 WU (Woods Units). The primary outcomes were reduction in PVR at 72 hours and sustained PVR improvement throughout Impella supported period. We also evaluated transplant outcomes, particularly the need for combined heart‐lung transplantation.

**Results:** The median age at Impella placement was 61 (52 – 66), with baseline LVEF 17% (15‐22). At 72 hours post‐Impella 5.5 support, all patients exhibited a significant reduction in mean PVR, from 3.16 WU to 1.77 WU (p < 0.001). In the PVR ≥ 4 WU cohort, PVR mean decreased from 5.5 WU to 2.45 WU (p < 0.01), reaching levels that enabled successful heart transplantation without lung transplantation. Importantly, these reductions were sustained throughout the duration of Impella support (median 24, 14‐39 days), ensuring transplant eligibility and reducing post‐transplant complications. All patients survived heart transplantation. No patients required post‐transplant right ventricular MCS.

**Conclusion:** Early and sustained PVR reduction after Impella 5.5 placement may shift clinical practice in managing transplant candidates with PH. Achieving hemodynamic stability within 72 hours could expedite heart transplantation, reducing the need for prolonged MCS, or lung transplants. These findings suggest Impella 5.5 may expand transplant eligibility for high‐risk patients with pulmonary hypertension.

## 155. Pulmonary Vascular Resistance and Differential Renal Recovery: Insights From Impella 5.5 Support

Klingbeil R^1^, Desai A^1^, Shapiro A^1^, Ruiz J^1^, Martin A^1^, Goswami R^1^

^1^Mayo Clinic, Jacksonville, United States

**Background:** Pulmonary vascular resistance (PVR) and renal function, as measured by glomerular filtration rate (GFR), are key hemodynamic parameters in patients with cardiogenic shock undergoing mechanical circulatory support. Reducing PVR not only improves cardiac outcomes but may also enhance renal perfusion. This study evaluates the relationship between changes in PVR and GFR following Impella 5.5 implantation, with a focus on differences between patients with low (< 4 Wood units) and elevated PVR.

**Methods:** This retrospective cohort analyzed 74 patients with advanced heart failure who underwent Impella 5.5 implantation. Hemodynamic data, including baseline and 72‐hour post‐implantation PVR, were collected and cohort was stratified based on baseline PVR: ≥ 4 WU and < 4 WU (Wood Units). We assessed renal function post Impella‐supported PVR reduction at 72 hours.

**Results:** The data demonstrated a significant reduction in PVR following Impella 5.5 support in both groups (P < 0.05). However, patients with baseline PVR < 4 Wood units showed a greater improvement in GFR compared to those with higher baseline PVR. On average, the GFR increased by 15.5 ml/min/1.73m2 in the lower PVR group and by 6 ml/min/1.73m2 in the higher PVR group (p < 0.001). The sustained hemodynamic improvements in PVR were accompanied by notable renal recovery, suggesting a relationship between PVR reduction and enhanced renal perfusion.

**Conclusion:** This study highlights the importance of PVR management in optimizing cardiac and renal outcomes in cardiogenic shock. The greater renal benefit in patients with lower initial PVR suggests that early intervention with Impella 5.5 could be particularly advantageous in these patients.

## 156. The Importance of Pulmonary Hypertension Associations: Results From the Pulmonary Hypertension Global Patient Survey

Granato M^1^, Newman J^2^, Ghani H^2^, Fischer G^8^, Howard L^3^, Kurzyna M^4^, MacDonald L^5^, Meszaros G^6^, Otter E^8^, Stone M^7^, Toshner M^2^, Tschida M^8^, Pepke‐Zaba J^2^

^1^Pulmonary Hypertension Association, Washington, United States, ^2^University of Cambridge & Royal Papworth Hospital, Cambridge, UK, ^3^Imperial College Healthcare NHS Trust, London, UK, ^4^Europejskie Centrum Zdrowia Otwock, Otwock, Poland, ^5^Great Ormond Street Hospital, London, UK, ^6^ERN‐LUNG, Frankfurt, Germany, ^7^iOWNA Ltd., London, UK, ^8^Pulmonary Hypertension Association Europe, Vienna, Austria

**Background:** Pulmonary hypertension (PH) guidelines advise that patients should be encouraged to join patient associations providing education, emotional support and empowerment. The number of patients who join one of the numerous national PH associations (PHAs) available worldwide is unknown.

**Aim:** To understand the proportion of PH patients part of a PHA and their perspective on the most important areas of support.

**Methods:** Questions regarding PHAs were embedded into the PH Global Patient Survey, the first worldwide online survey for all PH groups adult and paediatric patients and carers. Methods have been extensively reported elsewhere, including the PVRI 2024 Congress.

**Results:** 54.8% (1445/2638) of adult responders reported affiliation to a PHA. Likely to be overestimated, however, as PHAs’ media channels were the primary route of dissemination. The most useful reported areas of support were:

• Information/education: 84.8%

• Patient meetings/support groups: 51.9%

• Informative website: 46.4%

• Awareness activities: 30.7%

• Regular magazine: 27.1%

• Treatment access assistance: 22.6%

• Social support applications: 16.4%

• Access to helpful devices: 14.5%

• Help communicate with healthcare provider: 14.0%

• 24/7 phone helpline: 12.0%

• Travel arrangement assistance: 7.3%

• Financial support in emergencies: 7.1%

• Other: 7.6%

Qualitative analysis of the free‐text responses showed themes of PHAs providing a sense of community, being a gateway to accessing clinical trials, providing practical support (financial benefits/grants, legal aid, disabled status) and a sense of purpose in providing support to newly diagnosed members. Patients report that help accessing and financing medication is a key expectation of patient organisations, but in countries where healthcare is free, patients focused on social support and education.

**Conclusions:** PHAs are a vital source of education, information and support for patients and their carers. They are under‐utilised organisations which all PH patients should be directed to by their healthcare teams.

## 157. Upregulated Hipk2 Supports Pro‐Survival Phenotype of Pulmonary Smooth Muscle Cells in Pulmonary Arterial Hypertension

Pena A^1^, Jiang L^2^, Goncharov D^2^, Avolio T^1^, Baust J^1^, Sebastiani A^1^, Scott I^1^, Shen Y^2^, Teos L^2^, Lin D^2^, Saiyed A^2^, Zhyvylo I^2^, Goncharova E^2^, Kudryashova T^1^

^1^University Of Pittsburgh, Pittsburgh, United States, ^2^University of California Davis, Davis, United States

**Introduction:** Pulmonary arterial hypertension (PAH) still remains a deadly disease and new therapeutic strategies to stop disease progression need to be developed. Among the key pathological processes in PAH which need to be targeted are increased proliferation and survival of PA smooth muscle cells (PASMC). We recently discovered that upregulation of homeodomain‐interacting protein kinase‐2 (HIPK2) in PAH PASMC supports PASMC increased proliferation, survival and metabolic reprogramming in PAH. However, the mechanisms of HIPK2 regulation and function in PAH require further investigation.

**Methods/Results:** Pharmacological inhibition of HIPK2 by tBID significantly decreased cell growth, proliferation, and induced apoptosis in human PAH PASMC. RNAseq analysis of tBID‐treated PAH PASMC identified deregulation of multiple genes associated with cell cycle, apoptosis, vascular smooth muscle contraction, mitotic spindle, and cyclin‐dependent kinase activity, as revealed by KEGG/GO enrichment analysis, and specifically downregulation of regulator of cytokinesis CEP55 and mitotic cell cycle regulator PKMYT1, also confirmed by immunoblot analysis. Immunocytochemical/immunoblot analyses demonstrated over‐accumulation of CEP55, PKMYT1 and pro‐proliferative transcriptional co‐activator YAP in human PAH PASMC. YAP and/or CEP55 depletion inhibited pro‐proliferative/pro‐survival protein kinase AKT, reduced cell proliferation, and induced apoptosis, suggesting that HIPK2 promotes increased proliferation and survival of PAH PASMC through YAP and CEP55, and potentially through PKMYT1. Pharmacological (verteporfin) or shRNA‐induced YAP downregulation decreased HIPK2/CEP55/PKMYT1 axis in PAH PASMC, suggesting that bi‐directional YAP‐HIPK2 crosstalk exists to support HIPK2/YAP/CEP55/PKMYT1 upregulation and hyperproliferation in PAH. Importantly, pharmacological inhibition of HIPK2 in severe PH Sugen‐Hypoxia rat model demonstrated improvement of systolic right ventricular pressure in tBid‐treated rats compared to the vehicle‐treated group.

**Conclusion:** HIPK2 supports pro‐proliferative/pro‐survival PASMC phenotype in PAH via up‐regulating CEP55, YAP and its pharmacological inhibition attenuates severe experimental PH in vivo. More studies will be performed to further evaluate benefits of HIPK2 targeting as a novel anti‐remodeling therapy for PAH. Funded by NIH/NHLBI R01HL166932 (TVK).

## 158. Use of Sotatercept in Patient With Severe Scleroderma‐Associated PAH With PCH/PVOD Phenotype

George M^1^, Rurak K, Rice J, Randall A, Pillitteri V, Malave A, Lahm T, Kim D

^1^National Jewish Health, Denver, United States

Our patient is a 62‐year‐old woman diagnosed with scleroderma PAH in 2015, who achieved low risk status on tadalafil, ambrisentan, and selexipag and was stable and active until early 2022 when she developed worsening hypoxemia, fatigue, and shortness of breath. Due to clinical worsening, she was transitioned from selexipag to intravenous (IV) treprostinil and after conversion developed profound hypoxemia with bilateral pleural effusions, increased lymph node enlargement, interstitial thickening on HRCT chest, concerning for development of PCH/PVOD. She was admitted to the ICU, weaned off IV treprostinil, diuresed, initiated on immunosuppressive therapy and referred for lung transplant evaluation, but declined to pursue transplant.

Over the next several months, she developed worsening hypoxemia and shortness of breath and right heart catheterization confirmed severe PAH with a severely reduced cardiac index (mean RAP 24 mmHg, PAP 69/32/46, mean PAOP 10, TD CO 2.0 L/min, CI 1.1 L/min/m2, TD PVR 18 WU). She was diuresed and IV epoprostenol was cautiously initiated and uptitrated to 17.5 ng/kg/min with improvement and normalization of cardiac index (mean RAP 7, PA 90/32/44, mean PAO 11, TD CO 4.3, CI 2.5, indirect Fick CO 3.9, CI 2.3, TD PVR 10.2 WU). At this time, she was initiated on sotatercept.

Due to sotatercept‐induced thrombocytopenia, she was dosed at the 0.3 mg/kg dosing every 4 weeks. Just after discharge, her BNP was 270.3 pg/mL and 6MWD on 10 L was 197.7 m with SpO2 nadir 81%. Over the next 3 months her 6MWD steadily increased with less hypoxemia and BNP also steadily decreased to 39.1 pg/mL. Clinically her fatigue has improved, and she is able to climb a flight of stairs having achieved a NYHA functional class 2 status.

To our knowledge this is the first report of use of sotatercept in a patient with PAH and a PCH/PVOD phenotype.

## 159. Elevated Vascular Hyaluronan as a Therapeutic Target in Pulmonary Hypertension Associated With Lung Fibrosis

Karmouty Quintana H^1,2^, Collum S^1^, Girard R^1^, W Mills T^1^, Akkanti B^2^

^1^Department of Biochemistry and Molecular Biology, McGovern Medical School at The University of Texas Health Science Center at Houston, Houston, United States, ^2^Department of Internal Medicine, Division of Pulmonary, Critical Care, and Sleep Medicine, McGovern Medical School at The University of Texas Health Science Center at Houston, Houston, United States

**Background:** Pulmonary hypertension (PH) is a highly lethal and widespread pulmonary disorder that is a common complication in patients with interstitial lung disease (ILD), where it is the single most significant predictor of mortality. A central hallmark of PH is vascular remodeling, which is characterized by the expansion of the smooth muscle layer of the vessel, leading to the obliteration of the lumen. At present, there are limited treatment options outside lung transplantation. Treprostinil, a prostacyclin analog, is the only approved treatment for PH associated with lung fibrosis. However, its primary mechanism of action is vasodilation, with a limited capacity to alter the remodeled vasculature. Thus, there is a need to understand the mechanisms that lead to vascular remodeling in fibrotic lung disease. A central feature of lung fibrosis is the deposition of hyaluronan, a glycosaminoglycan and extracellular matrix (ECM) constituent, yet whether hyaluronan is elevated in fibrotic lung disease and its pathological role is not fully understood.

**Results:** Utilizing our human lung explant biorepository, we demonstrate increased perivascular deposition of hyaluronan in patients with a diagnosis of ILD, including idiopathic pulmonary fibrosis (IPF), combined pulmonary fibrosis, and emphysema (CPFE) and post‐COVID‐19 lung fibrosis requiring transplantation. The studies demonstrate the prevalence of elevated vascular hyaluronan in fibrotic lung diseases. Next, utilizing distinct experimental models of lung fibrosis and PH, we demonstrate that genetic or pharmacological deletion of hyaluronan attenuates experimental PH. In vitro, experiments utilizing pulmonary artery smooth muscle cells in culture demonstrate that hyaluronan activated RhoA to induce cell proliferation and uncovered two unique and distinct mechanisms that lead to increased expression of hyaluronan synthase 2 (HAS2), a key enzyme in the hyper‐synthesis of hyaluronan.

**Conclusion:** Our data points to elevated vascular hyaluronan as a therapeutic target for PH associated with lung fibrosis.

